# Supplementary material for: Nuclear glycine decarboxylase suppresses STAT1-dependent MHC-I and promotes cancer immune evasion
Source: EMBO J. 2025 Sep 8;44(20):5712–33. doi: 10.1038/s44318-025-00557-3 (PMC12528744; doi:10.1038/s44318-025-00557-3)
Supplement: Supplementary file 1 — Appendix [file 44318_2025_557_MOESM1_ESM.pdf]

Appendix Information For

Nuclear GLDC inhibits MHC-I independently of its enzymatic activity to promote cancer immune evasion

Rui Liu, Hui-Fang Li, Qi Jiang, Jun-Ge Shi, Zi-Lun Ruan, Peng Ren, Yi-Nuo Li, Hong-Bing Shu & Shu Li*

*Correspondence should be addressed: Dr. Shu Li (shuli@whu.edu.cn)

This PDF file includes:

Appendix Figure S1 and Legend…………Page 2-3

Appendix Figure S2 and Legend…………Page 4-5

Appendix Figure S3 and Legend…………Page 6

Appendix Figure S4 and Legend…………Page 7-8

Appendix Figure S5 and Legend…………Page 9-11

Appendix Figure S6 and Legend…………Page 12-14

Appendix Figure S7 and Legend…………Page 15-16

Appendix Figure S8 and Legend…………Page 17-19

Appendix Figure S9 and Legend…………Page 20-21

Appendix Figure S10 and Legend………..Page 22-23

Appendix Figure S11 and Legend………..Page 24

Appendix Table S1………………………Page 25

Appendix Table S2………………………Page 26

Appendix Table S3………………………Page 27

Appendix Table S4………………………Page 28

Appendix Table S5………………………Page 29-30

Appendix Table S6………………………Page 31

Appendix Table S7………………………Page 32

Appendix Table S8………………………Page 33-34

Appendix Table S9………………………Page 35

Liu et al, Appendix Figure S1. Related to Figure 1

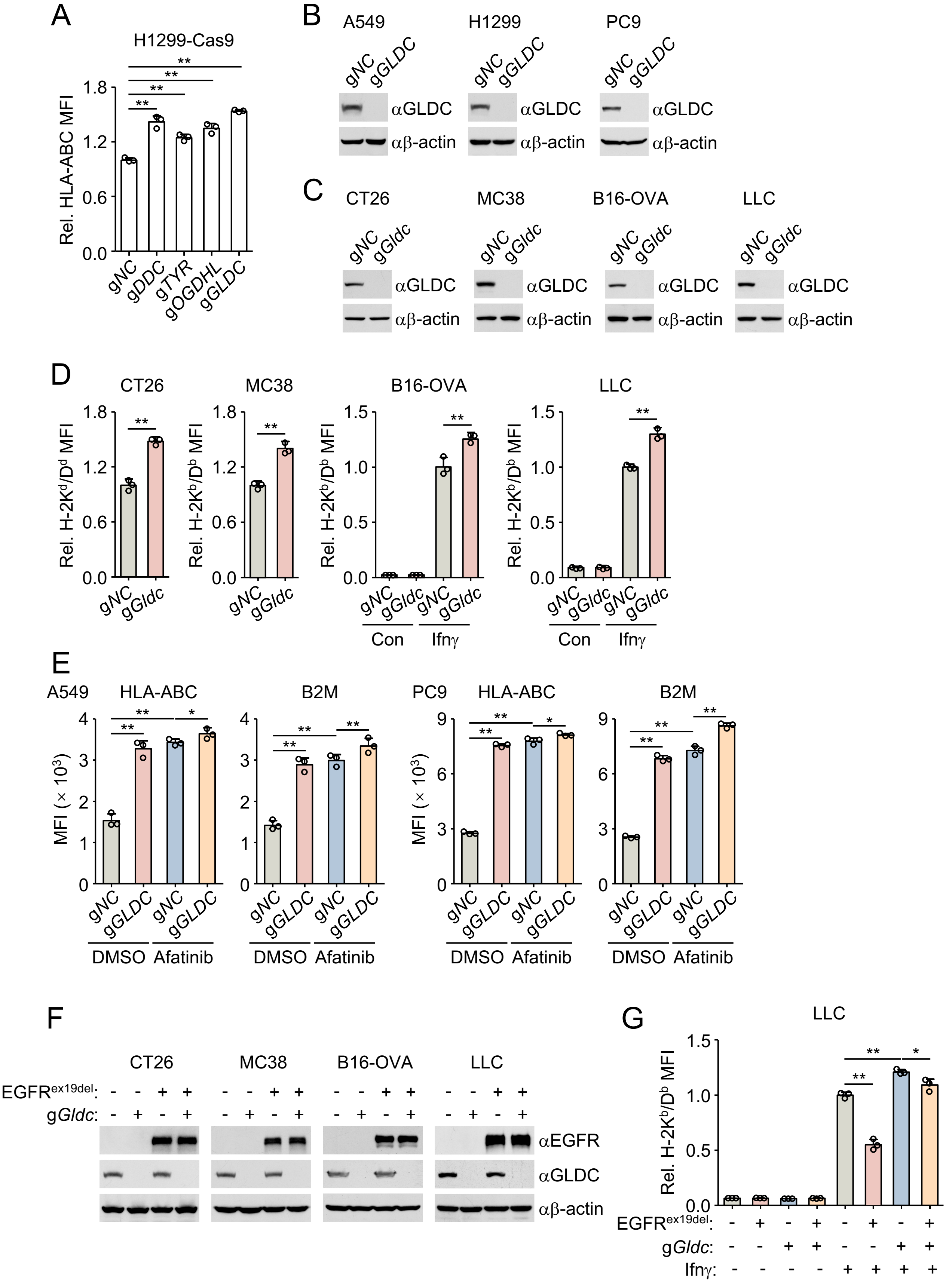

Appendix Figure S1. GLDC inhibits MHC-I surface expression independently of its enzymatic activity. Related to Figure 1.

(A) GLDC-deficiency enhances MHC-I surface expression. H1299-Cas9 cells were transduced with the indicated sgRNAs. The indicated cells were stained with the indicated antibodies and analyzed by flow cytometry. Graph shows mean ± SEM, n = 3 independent samples. Data were analyzed using a student's unpaired t-test with GraphPad Prism 8.

(B and C) GLDC protein levels in control and GLDC-deficient cells. Lysates of the indicated cells were analyzed by immunoblots with the indicated antibodies.

(D) GLDC-deficiency enhances MHC-I surface expression. CT26 or MC38 cells were stained with the indicated antibodies and analyzed by flow cytometry. B16-OVA or LLC cells were stimulated with Ifnγ (50 ng/mL) for 24 h before flow cytometry analysis with the indicated antibodies. Graph shows mean ± SEM, n = 3 independent samples. Data were analyzed using two-way ANOVA with GraphPad Prism 8.

(E) Afatinib treatment inhibits up-regulation of MHC-I induced by GLDC-deficiency. The indicated cells were treated with DMSO or Afatinib (2 μM) for 24 h before flow cytometry analysis with the indicated antibodies. Graph shows mean ± SEM, n = 3 independent samples. Data were analyzed using two-way ANOVA with GraphPad Prism 8.

(F) EGFR protein levels in $EGFR^{ex19del}$-transduced cells. Lysates of the indicated cells were analyzed by immunoblots with the indicated antibodies.

(G) Effects of EGFR activation on MHC-I surface expression. The indicated cells were stimulated with Ifnγ (50 ng/mL) for 24 h before flow cytometry analysis with the indicated antibodies. Graph shows mean ± SEM, n = 3 independent samples. Data were analyzed using two-way ANOVA with GraphPad Prism 8.

Liu et al, Appendix Figure S2. Related to Figure 1

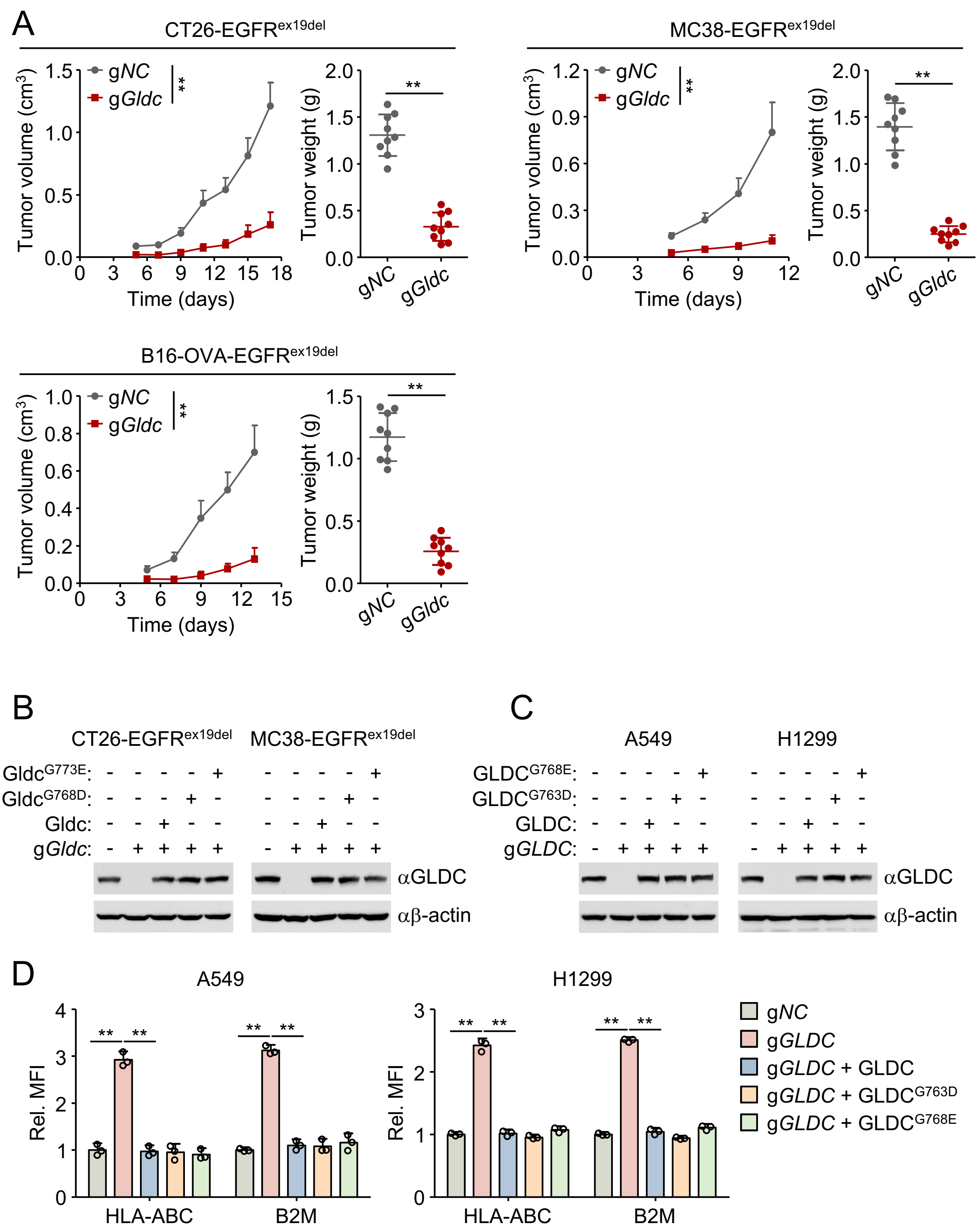

Appendix Figure S2. GLDC inhibits MHC-I surface expression independently of its enzymatic activity. Related to Figure 1.

(A) GLDC-deficiency suppresses tumor growth. The indicated cells were subcutaneously injected into mice. Tumor-bearing mice were euthanized on day 18 (CT26-EGFR^{ex19del} tumor), day 12 (MC38-EGFR^{ex19del} tumor) or day 14 (B16-OVA-EGFR^{ex19del} tumor), and then tumor tissues were separated from the mice. Tumor weights were measured by Analytical Balance. Graph shows mean ± SEM, n = 9 independent samples. Data were analyzed using a student's unpaired t-test with GraphPad Prism 8.

(B) Reconstitution of Gldc-deficient CT26-EGFR^{ex19del} or MC38-EGFR^{ex19del} cells with mouse wild-type Gldc, Gldc^{G768D} or Gldc^{G773E}. Lysates of the indicated cells were analyzed by immunoblots with the indicated antibodies.

(C) Reconstitution of GLDC-deficient A549 or H1299 cells with human wild-type GLDC, GLDC^{G763D} or GLDC^{G768E}. Lysates of the indicated cells were analyzed by immunoblots with the indicated antibodies.

(D) Effects of GLDC mutations on MHC-I surface expression. The indicated cells were cultured in the presence of EGF (20 ng/mL) for 24 h before flow cytometry analysis with the indicated antibodies. Graph shows mean ± SEM, n = 3 independent samples. Data were analyzed using two-way ANOVA with GraphPad Prism 8.

Liu et al, Appendix Figure S3. Related to Figure 2

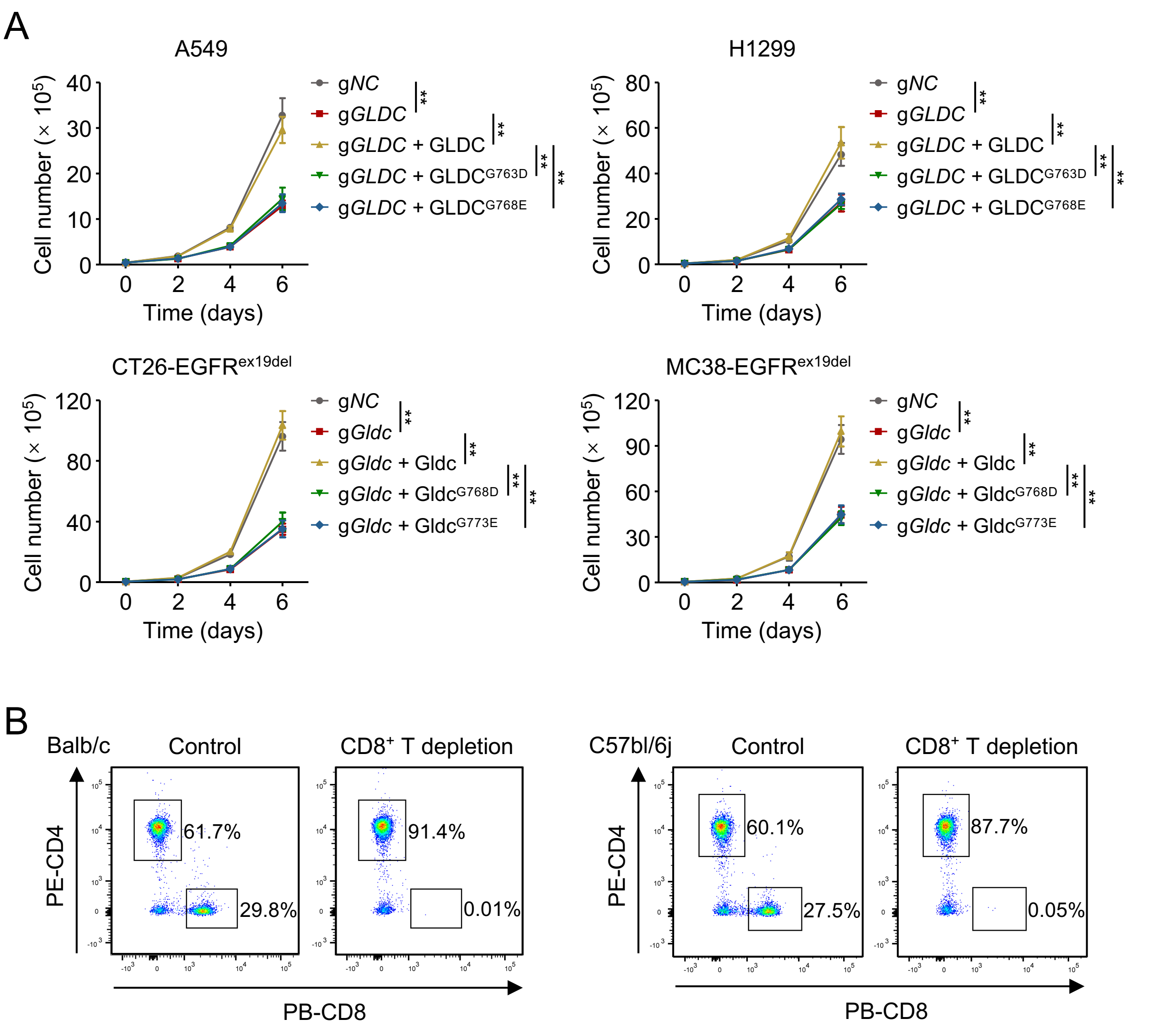

Appendix Figure S3. GLDC-deficiency inhibits cell proliferation. Related to Figure 2.

(A) Effects of GLDC mutations on the proliferation of tumor cells. Control (g*NC*) or GLDC-deficient (g*GLDC*) A549 or H1299 cells were reconstituted with human wild-type GLDC, $GLDC^{G763D}$ or $GLDC^{G768E}$. Control (g*NC*) or Gldc-deficient (g*Gldc*) $CT26\text{-}EGFR^{ex19del}$ or $MC38\text{-}EGFR^{ex19del}$ cells were reconstituted with mouse wild-type Gldc, $Gldc^{G768D}$ or $Gldc^{G773E}$. A549 or H1299 cells were cultured in the presence of EGF (20 ng/mL). The cells were subjected to proliferation analysis. Graph shows mean ± SEM, n = 3. Data were analyzed using two-way ANOVA with GraphPad Prism 8.

(B) The representative flow cytometric plots of splenocytes from Balb/c or C57bl/6j mice. Splenocytes from control or $CD8^+$ T cells depletion Balb/c or C57bl/6j mice were stained with the indicated antibodies and analyzed by flow cytometry.

Liu et al, Appendix Figure S4. Related to Figure 3

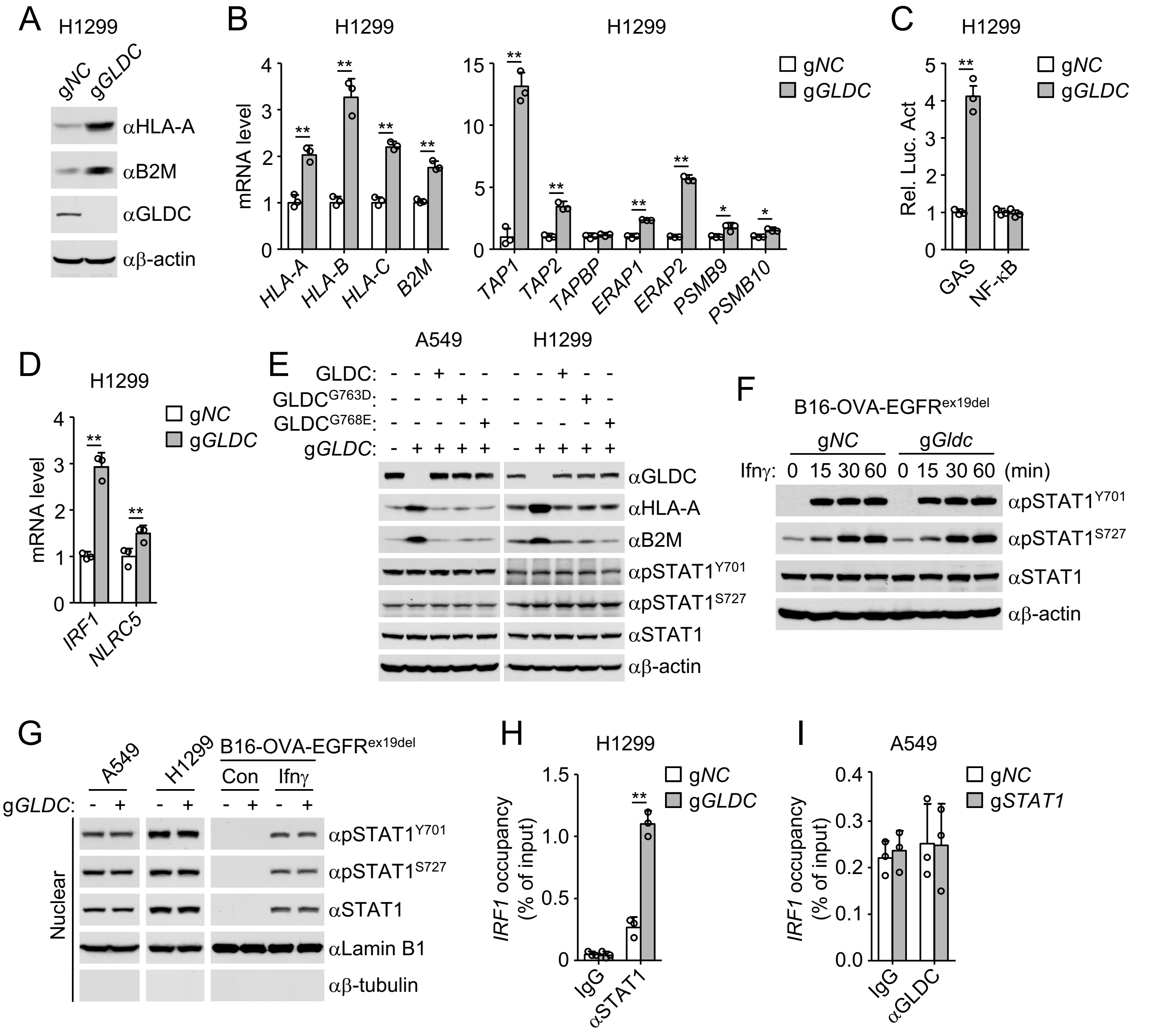

Appendix Figure S4. GLDC suppresses STAT1-triggered MHC-I antigen presentation. Related to Figure 3.

(A) GLDC-deficiency up-regulates the protein levels of HLA-A and B2M. The indicated H1299 cells were cultured in the presence of EGF (20 ng/mL) for 24 h before immunoblotting analysis with the indicated antibodies.

(B) Effects of GLDC-deficiency on transcription of major components involved in antigen presentation pathway and processing. The indicated H1299 cells were cultured in the presence of EGF (20 ng/mL) for 24 h before qPCR analysis of mRNA levels of the indicated genes. Graph shows mean ± SEM, n = 3 independent samples. Data were analyzed using a student's unpaired t-test with GraphPad Prism 8.

Appendix Figure S4. GLDC suppresses STAT1-triggered MHC-I antigen presentation. Related to Figure 3.

(C) GLDC-deficiency activates GAS. The indicated H1299 cells were cultured in the presence of EGF (20 ng/mL) and transfected with GAS or NF-κB reporter plasmids for 24 h before luciferase assays. Graph shows mean ± SEM, n = 3 independent samples. Data were analyzed using a student's unpaired t-test with GraphPad Prism 8.

(D) Effects of GLDC-deficiency on the transcription of *IRF1* and *NLRC5* genes. The indicated H1299 cells were cultured in the presence of EGF (20 ng/mL) for 24 h before qPCR analysis of mRNA levels of the indicated genes. Graph shows mean ± SEM, n = 3 independent samples. Data were analyzed using a student's unpaired t-test with GraphPad Prism 8.

(E) GLDC-deficiency up-regulates protein levels of HLA-A and B2M. The indicated A549 or H1299 cells were reconstituted with human wild-type GLDC, $GLDC^{G763D}$ or $GLDC^{G768E}$. The cells were cultured in the presence of EGF (20 ng/mL) for 24 h before immunoblotting analysis with the indicated antibodies.

(F) Effects of GLDC-deficiency on IFNγ-induced phosphorylation of STAT1. The indicated B16-OVA-$EGFR^{ex19del}$ cells were stimulated with Ifnγ (50 ng/mL) for the indicated time before immunoblotting analysis with the indicated antibodies.

(G) Effects of GLDC-deficiency on IFNγ-induced STAT1 nuclear translocation. The indicated A549 or H1299 cells were cultured in the presence of EGF (20 ng/mL) for 24 h. The indicated B16-OVA-$EGFR^{ex19del}$ cells were stimulated with Ifnγ (50 ng/mL) for 24 h. The cells were collected for subcellular fractionation experiments and immunoblotting analysis with the indicated antibodies.

(H) GLDC suppresses the binding ability of STAT1 to IRF1 promoter region. The indicated H1299 cells were cultured in the presence of EGF (20 ng/mL) for 24 h before ChIP analysis. The de-crosslinked DNA was subjected to qPCR analysis using specific primers. Graph shows mean ± SEM, n = 3 independent samples. Data were analyzed using two-way ANOVA with GraphPad Prism 8.

(I) Effects of STAT1-deficiency on the binding of GLDC to *IRF1* promoter region. The indicated A5499 cells were cultured in the presence of EGF (20 ng/mL) for 24 h before ChIP analysis. The de-crosslinked DNA was subjected to qPCR analysis using specific primers. Graph shows mean ± SEM, n = 3 independent samples. Data were analyzed using two-way ANOVA with GraphPad Prism 8.

Liu et al, Appendix Figure S5. Related to Figure 4

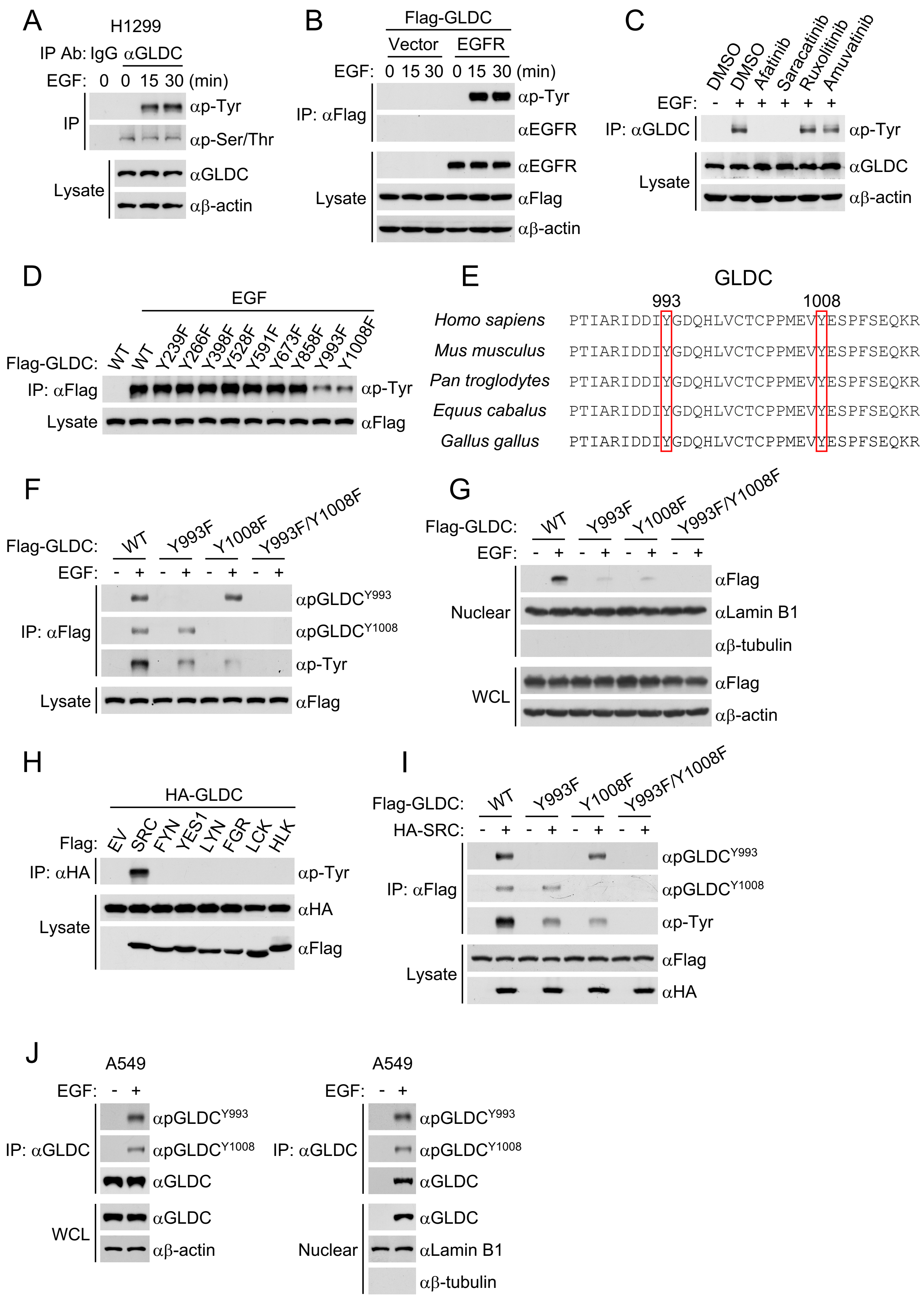

Appendix Figure S5. SRC catalyzes phosphorylation of GLDC at Y993 and Y1008. Related to Figure 4.

(A) EGF stimulation induces GLDC tyrosine phosphorylation. H1299 cells were serum-starved (12 h) and then treated with or without EGF (100 ng/ml) for the indicated times before co-immunoprecipitation and immunoblotting analysis with the indicated antibodies.

(B) GLDC is phosphorylated after EGF treatment in HEK293/EGFR cells. HEK293 cells were transduced with Vector or EGFR (HEK293/EGFR). The cells were transfected with the indicated plasmids for 24 h and then treated with or without EGF (100 ng/ml) for the indicated times before co-immunoprecipitation and immunoblotting analysis with the indicated antibodies.

(C) Effects of a panel of inhibitors on EGF-induced phosphorylation of GLDC. A549 cells were serum-starved (12 h) and then treated with Afatinib (2 μM), Saracatinib (1 μM), Ruxolitinib (5 μM) or Amuvatinib (5 μM) for 2 h before EGF (100 ng/ml) treatment for 30 min. The cells were collected for co-immunoprecipitation and immunoblotting analysis with the indicated antibodies.

(D) Effects of GLDC mutations on EGF-induced phosphorylation of GLDC. HEK293/EGFR cells were transfected with the indicated plasmids for 24 h and then treated with or without EGF (100 ng/ml) for 30 min before co-immunoprecipitation and immunoblotting analysis with the indicated antibodies.

(E) Sequence alignment of GLDC from the indicated species. The sequences are corresponding to aa984-1017 of human GLDC.

(F) EGF treatment fails to induce $GLDC^{Y993F/Y1008F}$ phosphorylation. HEK293/EGFR cells were transfected with the indicated plasmids for 24 h and then treated with or without EGF (100 ng/ml) for 30 min before co-immunoprecipitation and immunoblotting analysis with the indicated antibodies.

(G) $GLDC^{Y993/1008F}$ fails to be translocated into the nucleus following EGF treatment. HEK293/EGFR cells were transfected with the indicated plasmids for 24 h and then treated with or without EGF (100 ng/ml) for 6 h before subcellular fractionation experiments and immunoblotting analysis with the indicated antibodies.

(H) Effects of SRC family kinases on GLDC phosphorylation. HEK293 cells were transfected with the indicated plasmids for 24 h before co-immunoprecipitation and immunoblotting analysis with the indicated antibodies.

Appendix Figure S5. SRC catalyzes phosphorylation of GLDC at Y993 and Y1008. Related to Figure 4.

(I) $GLDC^{Y993F/Y1008F}$ fails to be phosphorylated by overexpression of SRC. HEK293 cells were transfected with the indicated plasmids for 24 h before co-immunoprecipitation and immunoblotting analysis with the indicated antibodies.

(J) GLDC remains phosphorylated upon nuclear translocation. A549 cells were serum-starved (12 h) and then treated with or without EGF (100 ng/ml) for 6 h. The cells were collected for subcellular fractionation experiments before co-immunoprecipitation and immunoblotting analysis with the indicated antibodies.

Liu et al, Appendix Figure S6. Related to Figure 4

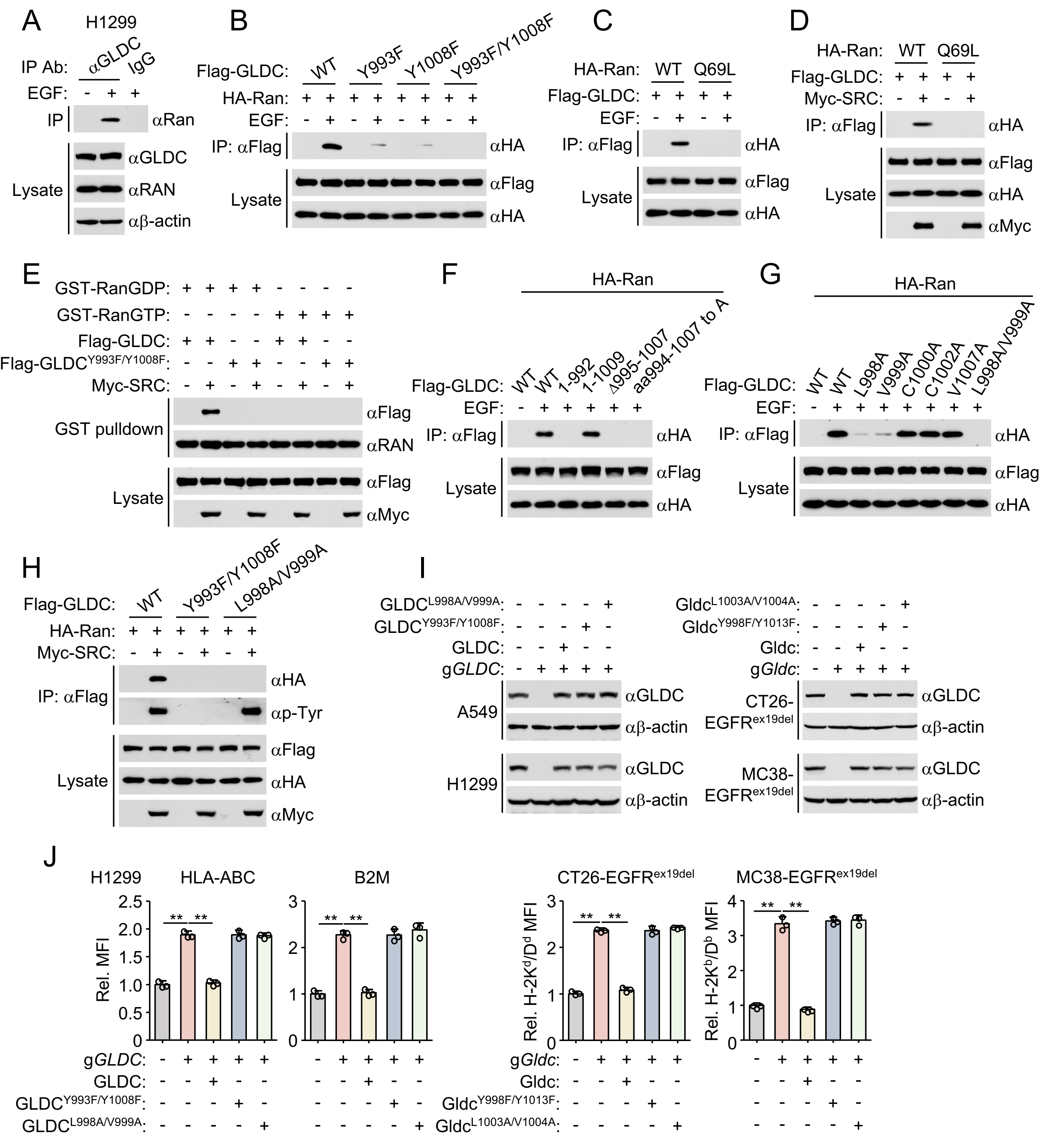

Appendix Figure S6. Ran mediates GLDC nuclear translocation. Related to Figure 4.

(A) EGF treatment induces the interaction between GLDC and Ran. H1299 cells were serum-starved (12 h) and then treated with or without EGF (100 ng/ml) for 30 min before co-immunoprecipitation and immunoblotting analysis with the indicated antibodies.

(B) Effects of GLDC mutations on the interaction of GLDC with Ran. HEK293/EGFR cells were transfected with the indicated plasmids for 24 h and then treated with or without EGF (100 ng/ml) for 30 min before co-immunoprecipitation and immunoblotting analysis with the indicated antibodies.

(C and D) Effects of Ran mutations on the interaction of GLDC with Ran. HEK293/EGFR cells were transfected with the indicated plasmids for 24 h and then treated with or without EGF (100 ng/ml) for 30 min (C). HEK293 cells were transfected with the indicated plasmids for 24 h (D). The cells were collected for co-immunoprecipitation and immunoblotting analysis with the indicated antibodies.

(E) GLDC is associated with RanGDP. HEK293 cells were transfected with the indicated plasmids for 24 h. The indicated recombinant GST-Ran proteins were incubated with the indicated cell lysates for GST pulldown assay before immunoblotting analysis with the indicated antibodies.

(F) Effects of GLDC mutations on the interaction of GLDC with Ran. HEK293/EGFR cells were transfected with the indicated plasmids for 24 h and then treated with or without EGF (100 ng/ml) for 30 min before co-immunoprecipitation and immunoblotting analysis with the indicated antibodies.

(G) $GLDC^{L998A/V999A}$ fails to interact with Ran. HEK293/EGFR cells were transfected with the indicated plasmids for 24 h and then treated with or without EGF (100 ng/ml) for 30 min before co-immunoprecipitation and immunoblotting analysis with the indicated antibodies.

(H) Effects of GLDC mutations on the phosphorylation of GLDC. HEK293 cells were transfected with the indicated plasmids for 24 h before co-immunoprecipitation and immunoblotting analysis with the indicated antibodies.

Appendix Figure S6. Ran mediates GLDC nuclear translocation. Related to Figure 4.

(I) Reconstitution of GLDC-deficient A549, H1299, CT26-EGFR^{ex19del} or MC38-EGFR^{ex19del} cells with human wild-type GLDC, GLDC$^{Y993F/Y1008F}$ (or mouse GLDC$^{Y998F/Y1013F}$) or GLDC$^{L998A/V999A}$ (or mouse GLDCL$^{1003A/V1004A}$). Lysates of the indicated cells were analyzed by immunoblots with the indicated antibodies.

(J) Effects of GLDC mutations on MHC-I surface expression. Control (g*NC*) or GLDC-deficient (g*GLDC*) H1299 cells were reconstituted with human wild-type GLDC, GLDC$^{Y993F/Y1008F}$ or GLDC$^{L998A/V999A}$. The indicated H1299 cells were cultured in the presence of EGF (20 ng/mL) for 24 h before cytometry analysis with the indicated antibodies. Control (g*NC*) or GLDC-deficient (g*Gldc*) CT26-EGFR^{ex19del} or MC38-EGFR^{ex19del} cells were reconstituted with mouse wild-type Gldc, Gldc$^{Y998F/Y1013F}$ or GLDCL$^{1003A/V1004F}$. The indicated cells CT26-EGFR^{ex19del} or MC38-EGFR^{ex19del} were stained with the indicated antibodies and analyzed by flow cytometry. Graph shows mean ± SEM, n = 3 independent samples. Data were analyzed using two-way ANOVA with GraphPad Prism 8.

Liu et al, Appendix Figure S7. Related to Figure 5

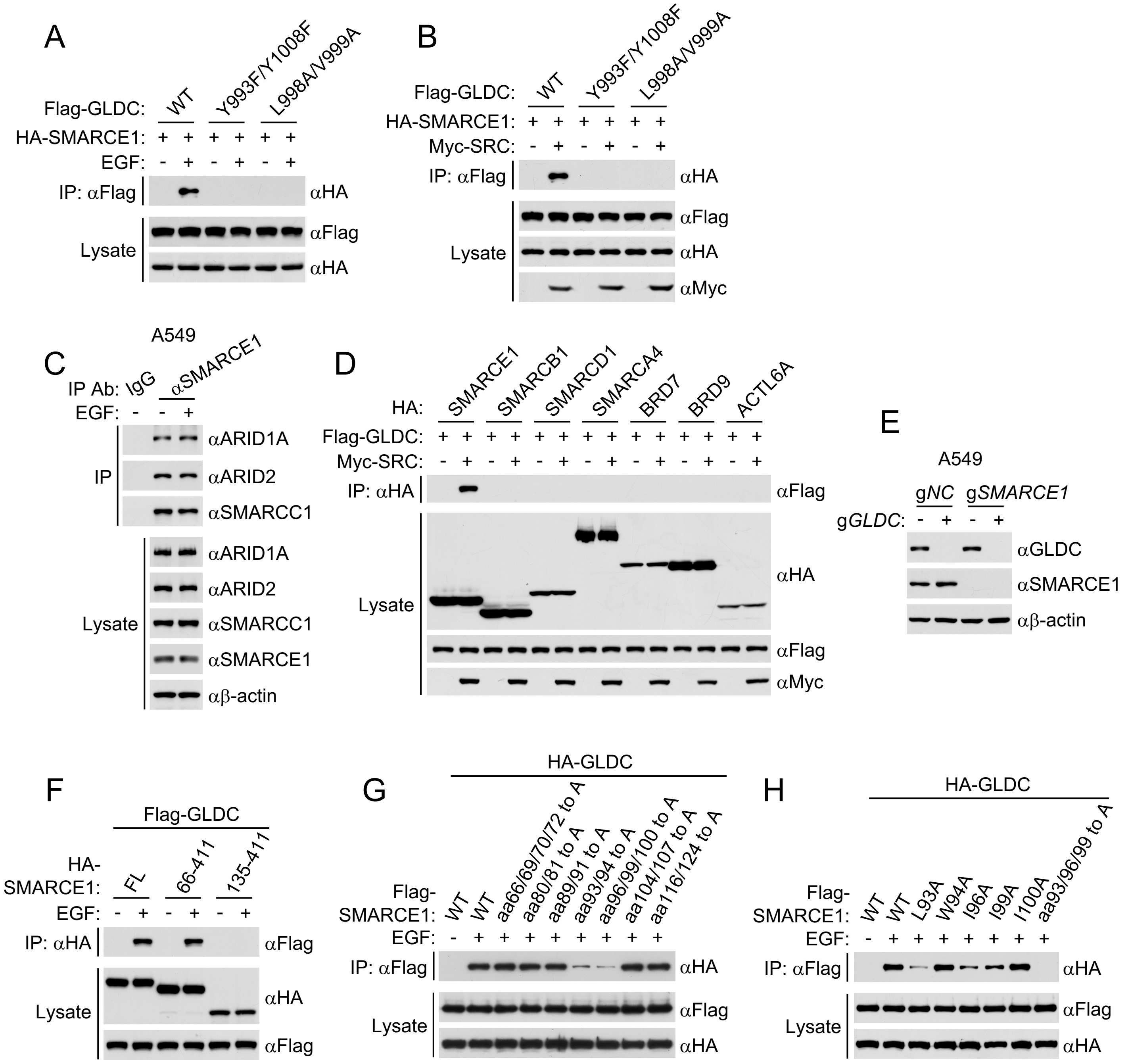

Appendix Figure S7. SMARCE1 is associated with GLDC. Related to Figure 5.

(A and B) Effects of GLDC mutations on the interaction of SMARCE1 with GLDC. HEK293/EGFR cells were transfected with the indicated plasmids for 24 h and then treated with or without EGF (100 ng/ml) for 6 h (A). HEK293 cells were transfected with the indicated plasmids for 24 h (B). The cells were collected for co-immunoprecipitation and immunoblotting analysis with the indicated antibodies.

(C) SMARCE1 was associated with the core components of SWI/SNF complex including ARID1A, ARID2 and SMARCC1. A549 cells were serum-starved (12 h) and then treated with or without EGF (100 ng/ml) for 6 h before co-immunoprecipitated and analyzed by immunoblotting with the indicated antibodies.

(D) SMARCE1 is associated with GLDC. HEK293 cells were transfected with the indicated plasmids for 24 before co-immunoprecipitation and immunoblotting analysis with the indicated antibodies.

(E) GLDC and SMARCE1 protein levels in control, GLDC-deficiency, SMARCE1-deficiency and GLDC/SMARCE1-deficiency cells. Lysates of the indicated cells were analyzed by immunoblots with the indicated antibodies.

(F) Effects of SMARCE1 truncation mutants on the interaction of GLDC with SMARCE1. HEK293/EGFR cells were transfected with the indicated plasmids for 24 h and then treated with or without EGF (100 ng/ml) for 6 h before co-immunoprecipitation and immunoblotting analysis with the indicated antibodies.

(G and H) Effects of SMARCE1 mutations on the interaction of GLDC with SMARCE1. HEK293/EGFR cells were transfected with the indicated plasmids for 24 h and then treated with or without EGF (100 ng/ml) for 6 h before co-immunoprecipitation and immunoblotting analysis with the indicated antibodies.

Liu et al, Appendix Figure S8. Related to Figure 5

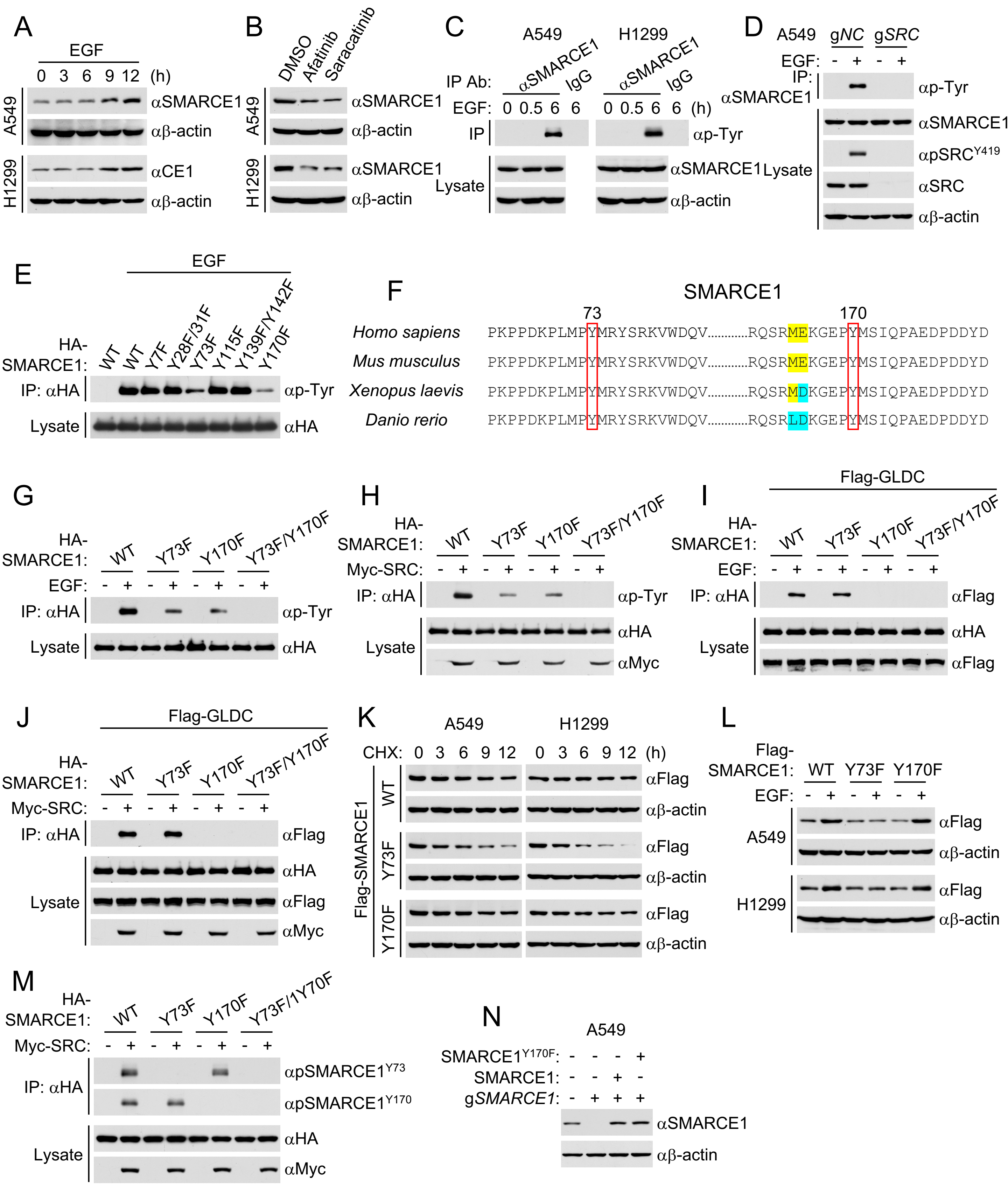

Appendix Figure S8. SRC mediates phosphorylation of SMARCE1. Related to Figure 5.

(A) EGF stimulation increases SMARCE1 protein level. A549 or H1299 cells were serum-starved (12 h) and then treated with or without EGF (100 ng/ml) for the indicated times before immunoblotting with the indicated antibodies.

(B) Inhibition of SRC down-regulates SMARCE1 protein levels. A549 or H1299 cells were cultured in the presence of EGF (50 ng/mL) for 24 h and then treated with DMAO, Afatinib (2 μM) or Saracatinib (1 μM) for 12 h before immunoblotting with the indicated antibodies.

(C) EGF stimulation induces SMARCE1 tyrosine phosphorylation. A549 or H1299 cells were serum-starved (12 h) and then treated with or without EGF (100 ng/ml) for the indicated times before co-immunoprecipitation and immunoblotting analysis with the indicated antibodies.

(D) Knockout of SRC inhibits EGF-induced phosphorylation of SMARCE1. Control (g*NC*) or SRC-deficient (g*SRC*) A549 cells were serum-starved (12 h) and then treated with or without EGF (100 ng/ml) for 6 h before co-immunoprecipitation and immunoblotting analysis with the indicated antibodies.

(E) Effects of SMARCE1 mutations on EGF-induced phosphorylation of SMARCE1. HEK293/EGFR cells were transfected with the indicated plasmids for 24 h and then treated with or without EGF (100 ng/ml) for 6 h before co-immunoprecipitation and immunoblotting analysis with the indicated antibodies.

(F) Sequence alignment of SMARCE1 from the indicated species. The sequences are corresponding to aa60-84 and aa158-185 of human SMARCE1.

(G and H) SMARCE1$^{Y73/170F}$ fails to be phosphorylated by EGF treatment or overexpression of SRC. HEK293/EGFR cells were transfected with the indicated plasmids for 24 h and then treated with or without EGF (100 ng/ml) for 6 h (G). HEK293 cells were transfected with the indicated plasmids for 24 h (H). The cells were collected for co-immunoprecipitation and immunoblotting analysis with the indicated antibodies.

Appendix Figure S8. SRC mediates phosphorylation of SMARCE1. Related to Figure 5.

(I and J) Effects of SMARCE1 mutations on the interaction of GLDC with SMARCE1. HEK293/EGFR cells were transfected with the indicated plasmids for 24 h and then treated with or without EGF (100 ng/ml) for 6 h (I). HEK293 cells were transfected with the indicated plasmids for 24 h (J). The cells were collected for co-immunoprecipitation and immunoblotting analysis with the indicated antibodies.

(K) Phosphorylation of SMARCE1^{Y73} inhibits its degradation. A549 and H1299 cells were transduced with the indicated plasmids. The cells were cultured in the presence of EGF (20 ng/mL) for 24 h and then treated with CHX (0.1 mM) for the indicated times before immunoblotting analysis with the indicated antibodies.

(L) Phosphorylation of SMARCE1^{Y73} enhances its stability. A549 and H1299 cells were transduced with the indicated plasmids. The cells were serum-starved (12 h) and then treated with or without EGF (100 ng/ml) for 12 h before immunoblotting analysis with the indicated antibodies.

(M) Effects of SMARCE1 mutations on SRC-medicated phosphorylation of SMARCE1. HEK293 cells were transfected with the indicated plasmids for 24 h before co-immunoprecipitation and immunoblotting analysis with the indicated antibodies.

(N) Reconstitution of SMARCE1-deficient A549 cells with wild-type SMARCE1 or SMARCE1^{Y170F}. Lysates of the indicated cells were analyzed by immunoblots with the indicated antibodies.

Liu et al, Appendix Figure S9. Related to Figure 5

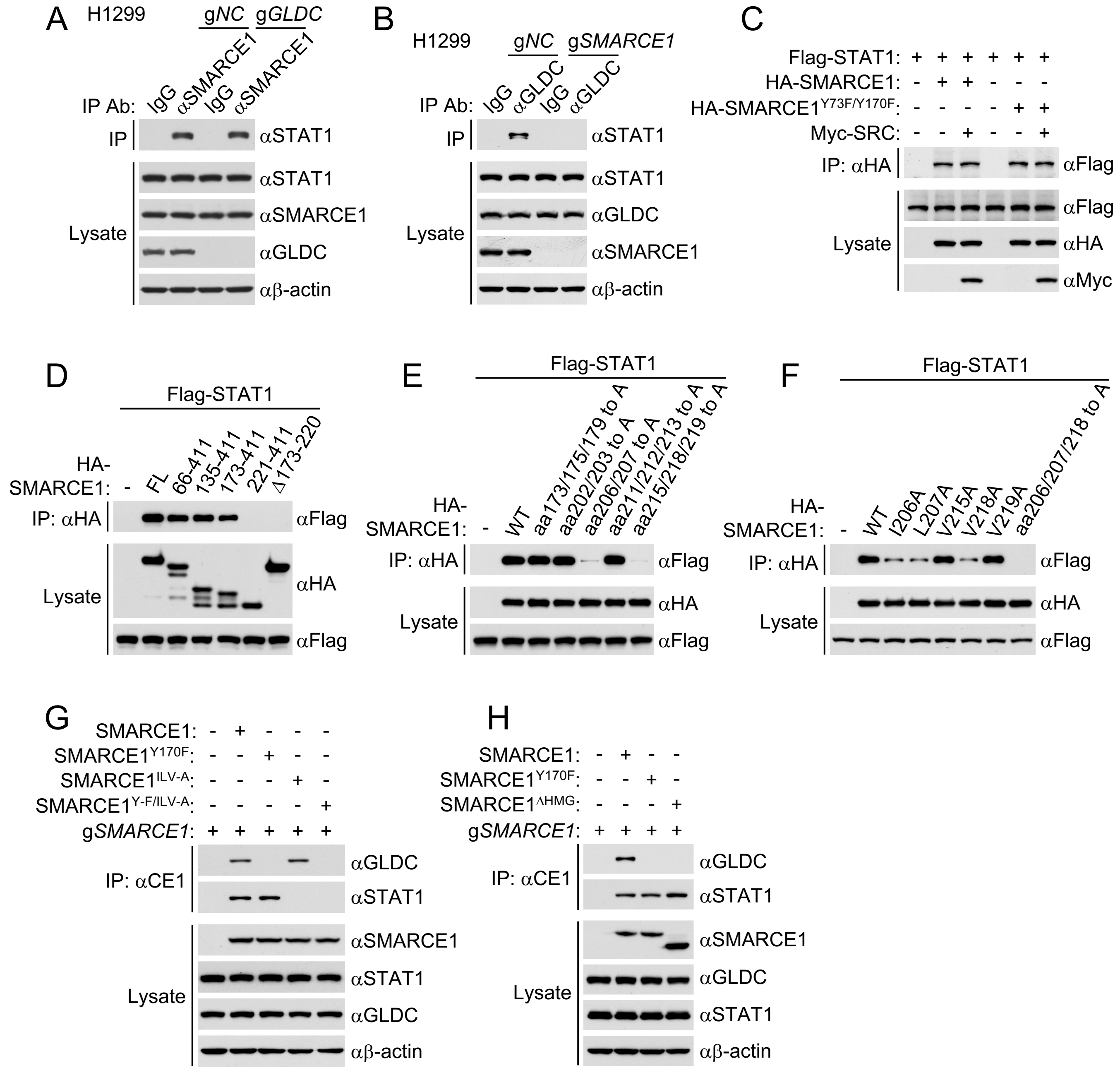

Appendix Figure S9. SMARCE1 is associated with STAT1. Related to Figure 5.

(A) Effects of GLDC-deficiency on the interaction of SMARCE1 with STAT1. Control (g*NC*) or GLDC-deficient (g*GLDC*) H1299 cells were cultured in the presence of EGF (20 ng/mL) for 24 h before co-immunoprecipitation and immunoblotting analysis with the indicated antibodies.

(B) Effects of SMARCE1-deficiency on the interaction of GLDC with STAT1. Control (g*NC*) or SMARCE1-deficient (g*SMARCE1*) H1299 cells were cultured in the presence of EGF (20 ng/mL) for 24 h before co-immunoprecipitation and immunoblotting analysis with the indicated antibodies.

(C) Effects of SMARCE1 mutations on the interaction of STAT1 with SMARCE1. HEK293 cells were transfected with the indicated plasmids for 24 h before co-immunoprecipitation and immunoblotting analysis with the indicated antibodies.

(D) Effects of SMARCE1 truncation mutants on the interaction of STAT1 with SMARCE1. HEK293 cells were transfected with the indicated plasmids for 24 h before co-immunoprecipitation and immunoblotting analysis with the indicated antibodies.

(E and F) Effects of SMARCE1 mutations on the interaction of STAT1 with SMARCE1. HEK293 cells were transfected with the indicated plasmids for 24 h before co-immunoprecipitation and immunoblotting analysis with the indicated antibodies.

(G) Reconstitution of SMARCE1-deficient (g*SMARCE1*) A549 cells with wild-type SMARCE1, SMARCE1^{Y170F}, SMARCE1$^{I206A/L207A/V218A}$ (SMARCE1$^{ILV\text{-}A}$) or SMARCE1$^{Y170F\ \&\ I206A/L207A/V218A}$ (SMARCE1$^{Y\text{-}F/ILV\text{-}A}$). . he cells were cultured in the presence of EGF (20 ng/mL) for 24 h before co-immunoprecipitation and immunoblotting analysis with the indicated antibodies.

(H) Reconstitution of SMARCE1-deficient (g*SMARCE1*) A549 cells with wild-type SMARCE1, SMARCE1^{Y170F} or SMARCE1$^{\Delta HMG}$. The cells were cultured in the presence of EGF (20 ng/mL) for 24 h before co-immunoprecipitation and immunoblotting analysis with the indicated antibodies.

Liu et al, Appendix Figure S10. Related to Figure 6

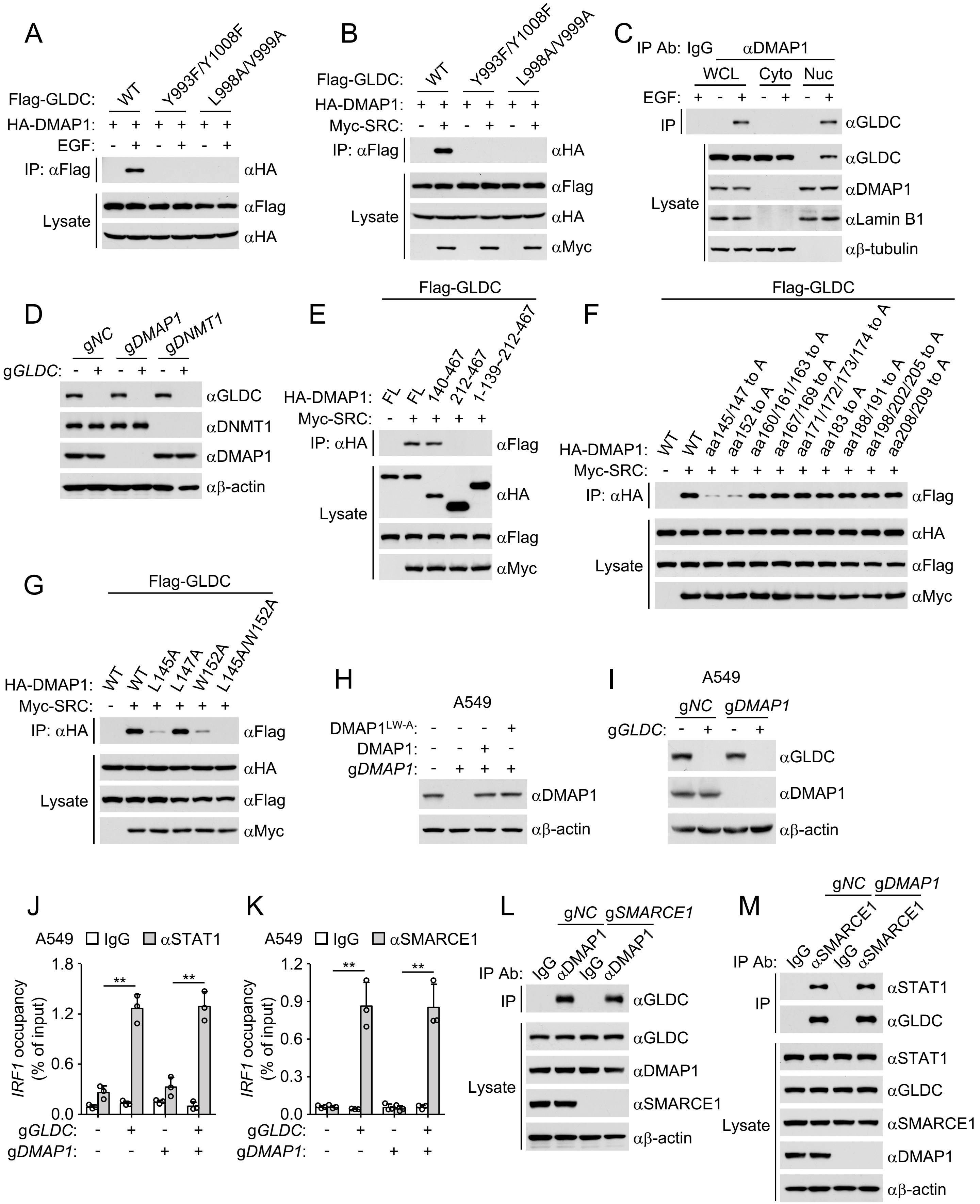

Appendix Figure S10. GLDC is associated with DMAP1. Related to Figure 6.

(A and B) Effects of GLDC mutations on the interaction of DMAP1 with GLDC. HEK293/EGFR cells were transfected with the indicated plasmids for 24 h and then treated with or without EGF (100 ng/ml) for 6 h (A). HEK293 cells were transfected with the indicated plasmids for 24 h (B). The cells were collected for co-immunoprecipitation and immunoblotting analysis with the indicated antibodies.

(C) EGF induces the interaction of GLDC with DMAP1 in nucleus. A549 cells were serum-starved (12 h) and then treated with or without EGF (100 ng/ml) for 6 h before subcellular fractionation experiments. Subcellular fractions were co-immunoprecipitated and analyzed by immunoblotting with the indicated antibodies.

(D) GLDC, DMAP1 and DNMT1 protein levels in control, GLDC-deficiency, DMAP1-deficiency, DNMT1-deficiency, GLDC/DMAP1-deficiency and GLDC/DNMT1 deficiency cells.

(E-G) Effects of DMAP1 mutations on the interaction of DMAP1 with GLDC. HEK293 cells were transfected with the indicated plasmids for 24 h before co-immunoprecipitation and immunoblotting analysis with the indicated antibodies.

(H and I) Reconstitution of DMAP1-deficient A549 cells with wild-type DMAP1 or DMAP1$^{L145A/W152A}$ (DMAP1^{LW-A}) (H). GLDC and DMAP1 protein levels in control, GLDC-deficiency, DMAP1-deficiency and GLDC/DMAP1-deficiency cells (I).

(J and K) Effects of DMAP1 on the binding of STAT1 or SMARCE1 to *IRF1* promoter region. The indicated A549 cells were cultured in the presence of EGF (20 ng/mL) for 24 h before ChIP analysis. The de-crosslinked DNA was subjected to qPCR analysis using specific primers. Graph shows mean ± SEM, n = 3 independent samples. Data were analyzed using two-way ANOVA with GraphPad Prism 8.

(L) Effects of SMARCE1-deficiency on the interaction of DMAP1 with GLDC. The indicated A549 cells were cultured in the presence of EGF (20 ng/mL) for 24 h before co-immunoprecipitation and immunoblotting analysis with the indicated antibodies.

(M) Effects of DMAP1-deficiency on the interaction of SMARCE1 with GLDC or STAT1. The indicated A549 cells were cultured in the presence of EGF (20 ng/mL) for 24 h before co-immunoprecipitation and immunoblotting analysis with the indicated antibodies.

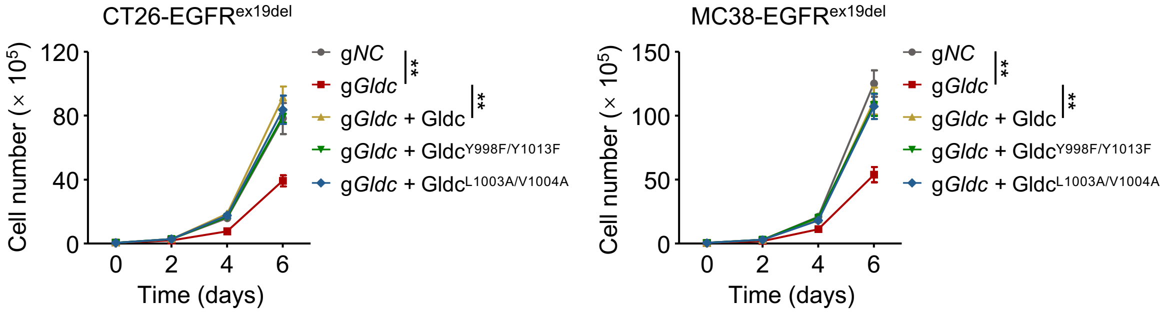

Appendix Figure S11. GLDC-deficiency inhibits cell proliferation. Related to Figure 7.

Control (g*NC*) or Gldc-deficient (g*Gldc*) CT26-EGFR^{ex19del} or MC38-EGFR^{ex19del} cells were reconstituted with mouse wild-type Gldc, Gldc$^{Y998F/Y1013F}$ or Gldc$^{L1003A/V1004F}$. The cells were subjected to proliferation analysis. Graph shows mean ± SEM, n = 3. Data were analyzed using two-way ANOVA with GraphPad Prism 8.

Appendix Table S1. A list of rate-limiting metabolic enzymes

ACADL	ALDH9A1	FBP2	HSD3B1	PKM	SOAT2
ACO1	ALOX5	G6PC	HSD3B2	PLAT	SPTLC1
ACO2	APRT	G2PC2	IMPDH1	PLAU	SPTLC2
ACSL1	ASS1	G6PD	IMPDH2	PTGS1	SQLE
ADH1A	BACE1	GAD1	LIPE	PTGS2	STS
ADH7	CHAT	GAD2	LPCAT2	PYGB	TAT
ADK	COX4I2	GLDC	LTA4H	PYGL	TK1
ALAD	COX5B	GNE	LTC4S	PYGM	TPH1
ALAS1	COX6A2	GPAM	MYLK	RDH5	TPH2
ALAS2	COX6B1	GPD2	MYLK2	REN	TYMP
ALDH1A1	DCK	HDC	MYLK3	RRM1	TYR
ALDH1A2	DDC	HK1	OGDH	RRM2	UCK1
ALDH1A3	DLD	HK2	OGDHL	RRM2B	UCK2
ALDH1B1	DLST	HK3	PAH	SAT1	UCKL1
ALDH2	DPYD	HMGCR	PCK1	SAT2	UGDH
ALDH3A1	DTYMK	HMGCS1	PCK2	SCD	UGT2B4
ALDH3A2	F2	HMGCS2	PIK3C3	SCD5	XDH
ALDH7A1	FBP1	HSD17B6	PKLR	SOAT1	

Appendix Table S2. A list of tyrosine phosphorylation residues of GLDC after EGF treatment in mass spectrometry analysis

Sites	Phospho (STY) Probabilities	Intensity	Ratio mod/base
Y239	AKY(0.885)T(0.115)GVLTELK	91324	0.065388
Y398	ATSNICTAQALLANMAAMFAIY(0.997)HGS(0.003)HGLEHIAR	351700	0.020587
Y266	DVSGVLFQY(0.915)PDT(0.084)EGKVEDFT(0.001)ELVER	83740	0.006223
Y591	EFANIHPFVPLDQAQGY(1)QQLFR	161540	0.013473
Y858	GY(1)VGHEFILDTRPFK	227260	0.005077
Y993	IDDIY(1)GDQHLVCTCPP	749980	0.12404
Y1008	HLVCT(0.003)CPPMEVY(0.909)ES(0.079)PFS(0.01)EQK	159410	0.026366
Y673	Y(1)GNIDAVHLK	604350	0.01446
Y528	TSPFLTHQVFNS(0.027)Y(0.972)HS(0.001)ETNIVR	306820	0.019921

Appendix Table S3. A list of GLDC high-confidence interactors after EGF stimulation in mass spectrometry (EGF/Mock log2 FC > 3)

Accession	Gene	Description	Mw(kDa)	Length	EGF Intensity	Mock Intensity	log2 FC
Q9NPF5	DMAP1	DNA methyltransferase 1-associated protein 1 OS=Homo sapiens OX=9606 GN=DMAP1 PE=1 SV=1	52.993	467	535100	2179.8	7.93947
P0CG39	POTEJ	POTE ankyrin domain family member J OS=Homo sapiens OX=9606 GN=POTEJ PE=3 SV=1	117.39	1038	1592100	7738	7.68475
Q9H9A6	LRRC40	Leucine-rich repeat-containing protein 40 OS=Homo sapiens OX=9606 GN=LRRC40 PE=1 SV=1	68.25	602	116020	833.5	7.12098
O95613	PCNT	Pericentrin OS=Homo sapiens OX=9606 GN=PCNT PE=1 SV=4	378.037	3336	1174200	9453.9	6.95655
Q969G3	SMARCE1	SWI/SNF-related matrix-associated actin-dependent regulator of chromatin subfamily E member 1 OS=Homo sapiens OX=9606 GN=SMARCE1 PE=1	46.649	411	249030	2769.6	6.49050
Q9UHR5	SAP30BP	SAP30-binding protein OS=Homo sapiens OX=9606 GN=SAP30BP PE=1 SV=1	33.87	308	74994	1354.9	5.79052
O00161	SNAP23	Synaptosomal-associated protein 23 OS=Homo sapiens OX=9606 GN=SNAP23 PE=1 SV=1	23.354	211	136500	2900.8	5.55631
Q9NP79	VTA1	Vacuolar protein sorting-associated protein VTA1 homolog OS=Homo sapiens OX=9606 GN=VTA1 PE=1 SV=1	33.879	307	201040	4301.1	5.54663
Q9HAV4	XPO5	Exportin-5 OS=Homo sapiens OX=9606 GN=XPO5 PE=1 SV=1	136.311	1204	4188800	98703	5.40730
O75822	EIF3J	Eukaryotic translation initiation factor 3 subunit J OS=Homo sapiens OX=9606 GN=EIF3J PE=1 SV=2	29.062	258	630430	15303	5.36445
Q9ULR0	ISY1	Pre-mRNA-splicing factor ISY1 homolog OS=Homo sapiens OX=9606 GN=ISY1 PE=1 SV=3	32.992	285	38994	1054.2	5.20903
Q01130	SRSF2	Serine/arginine-rich splicing factor 2 OS=Homo sapiens OX=9606 GN=SRSF2 PE=1 SV=4	25.476	221	1787900	54243	5.04269
Q92522	H1-10	Histone H1.10 OS=Homo sapiens OX=9606 GN=H1-10 PE=1 SV=1	22.487	213	279430	8523.2	5.03495
P18615	NELFE	Negative elongation factor E OS=Homo sapiens OX=9606 GN=NELFE PE=1 SV=3	43.24	380	194760	6327	4.94403
P12004	PCNA	Proliferating cell nuclear antigen OS=Homo sapiens OX=9606 GN=PCNA PE=1 SV=1	28.769	261	84644	2814.3	4.91056
Q9HBI1	PARVB	Beta-parvin OS=Homo sapiens OX=9606 GN=PARVB PE=1 SV=1	41.714	364	28211	966.99	4.86661
Q92576	PHF3	PHD finger protein 3 OS=Homo sapiens OX=9606 GN=PHF3 PE=1 SV=3	229.481	2039	101430	3629	4.80477
Q9Y5S9	RBM8A	RNA-binding protein 8A OS=Homo sapiens OX=9606 GN=RBM8A PE=1 SV=1	19.889	174	468950	17991	4.70409
Q9NXV6	CDKN2AIP	CDKN2A-interacting protein OS=Homo sapiens OX=9606 GN=CDKN2AIP PE=1 SV=3	61.125	580	55845	2308.9	4.59615
Q99590	SCAF11	Protein SCAF11 OS=Homo sapiens OX=9606 GN=SCAF11 PE=1 SV=2	164.652	1463	99856	4213.2	4.56686
P50914	RPL14	60S ribosomal protein L14 OS=Homo sapiens OX=9606 GN=RPL14 PE=1 SV=4	23.432	215	867280	37651	4.52574
Q9Y605	MRFAP1	MORF4 family-associated protein 1 OS=Homo sapiens OX=9606 GN=MRFAP1 PE=1 SV=1	14.65	127	51222	2225.5	4.52456
O43819	SCO2	Protein SCO2 homolog, mitochondrial OS=Homo sapiens OX=9606 GN=SCO2 PE=1 SV=3	29.81	266	45029	2033.7	4.46868
Q9UKB1	FBXW11	F-box/WD repeat-containing protein 11 OS=Homo sapiens OX=9606 GN=FBXW11 PE=1 SV=1	62.091	542	162190	7833.4	4.37190
Q52LJ0	FAM98B	Protein FAM98B OS=Homo sapiens OX=9606 GN=FAM98B PE=1 SV=2	45.547	433	584800	30276	4.27170
Q3ZCM7	TUBB8	Tubulin beta-8 chain OS=Homo sapiens OX=9606 GN=TUBB8 PE=1 SV=2	49.776	444	117960	6196.8	4.25063
Q9BVL2	NUP58	Nucleoporin p58/p45 OS=Homo sapiens OX=9606 GN=NUP58 PE=1 SV=1	60.897	599	1098200	58919	4.22026
P26640	VARS1	Valine--tRNA ligase OS=Homo sapiens OX=9606 GN=VARS1 PE=1 SV=4	140.476	1264	454010	24366	4.21978
P61244	MAX	Protein max OS=Homo sapiens OX=9606 GN=MAX PE=1 SV=1	18.275	160	74289	4412.8	4.07338
Q9P266	JCAD	Junctional protein associated with coronary artery disease OS=Homo sapiens OX=9606 GN=JCAD PE=1 SV=3	148.351	1359	3891000	241680	4.00897
Q7L804	RAB11FIP2	Rab11 family-interacting protein 2 OS=Homo sapiens OX=9606 GN=RAB11FIP2 PE=1 SV=1	58.279	512	58211	3821.8	3.92897
Q9Y4L1	HYOU1	Hypoxia up-regulated protein 1 OS=Homo sapiens OX=9606 GN=HYOU1 PE=1 SV=1	111.335	999	201060	14075	3.83642
Q8N3C0	ASCC3	Activating signal cointegrator 1 complex subunit 3 OS=Homo sapiens OX=9606 GN=ASCC3 PE=1 SV=3	251.46	2202	3462300	243860	3.82761
Q9NQB0	TCF7L2	Transcription factor 7-like 2 OS=Homo sapiens OX=9606 GN=TCF7L2 PE=1 SV=2	67.919	619	8961.9	640.56	3.80640
P55036	PSMD4	26S proteasome non-ATPase regulatory subunit 4 OS=Homo sapiens OX=9606 GN=PSMD4 PE=1 SV=1	40.737	377	168660	12502	3.75389
P62979	RPS27A	Ubiquitin-40S ribosomal protein S27a OS=Homo sapiens OX=9606 GN=RPS27A PE=1 SV=2	17.965	156	2739000	205450	3.73679
Q58FF8	HSP90AB2P	Putative heat shock protein HSP 90-beta 2 OS=Homo sapiens OX=9606 GN=HSP90AB2P PE=1 SV=2	44.349	381	630490	47735	3.72335
P23588	EIF4B	Eukaryotic translation initiation factor 4B OS=Homo sapiens OX=9606 GN=EIF4B PE=1 SV=2	69.151	611	237870	18034	3.72138
Q5T8P6	RBM26	RNA-binding protein 26 OS=Homo sapiens OX=9606 GN=RBM26 PE=1 SV=3	113.597	1007	83672	6352.4	3.71937
Q9H7E9	C8orf33	UPF0488 protein C8orf33 OS=Homo sapiens OX=9606 GN=C8orf33 PE=1 SV=1	24.993	229	54170	4190.7	3.69223
Q9UQR1	ZNF148	Zinc finger protein 148 OS=Homo sapiens OX=9606 GN=ZNF148 PE=1 SV=2	88.976	794	299320	24270	3.62444
Q13356	PPIL2	RING-type E3 ubiquitin-protein ligase PPIL2 OS=Homo sapiens OX=9606 GN=PPIL2 PE=1 SV=1	58.823	520	104940	8769.3	3.58096
Q13427	PPIG	Peptidyl-prolyl cis-trans isomerase G OS=Homo sapiens OX=9606 GN=PPIG PE=1 SV=2	88.617	754	85595	7362	3.53936
P22234	PAICS	Multifunctional protein ADE2 OS=Homo sapiens OX=9606 GN=PAICS PE=1 SV=3	47.079	425	467030	40448	3.52937
O94826	TOMM70	Mitochondrial import receptor subunit TOM70 OS=Homo sapiens OX=9606 GN=TOMM70 PE=1 SV=1	67.455	608	72192	6295.2	3.51951
O14579	COPE	Coatomer subunit epsilon OS=Homo sapiens OX=9606 GN=COPE PE=1 SV=3	34.482	308	277140	24360	3.50803
Q969S3	ZNF622	Zinc finger protein 622 OS=Homo sapiens OX=9606 GN=ZNF622 PE=1 SV=1	54.272	477	82390	7543.6	3.44914
P49588	AARS1	Alanine--tRNA ligase, cytoplasmic OS=Homo sapiens OX=9606 GN=AARS1 PE=1 SV=2	106.81	968	79596	7854.7	3.34107
O15357	INPPL1	Phosphatidylinositol 3,4,5-trisphosphate 5-phosphatase 2 OS=Homo sapiens OX=9606 GN=INPPL1 PE=1 SV=2	138.599	1258	58571	5802.5	3.33544
Q56P03	EAPP	E2F-associated phosphoprotein OS=Homo sapiens OX=9606 GN=EAPP PE=1 SV=4	32.762	285	35287	3504.6	3.33182
Q14145	KEAP1	Kelch-like ECH-associated protein 1 OS=Homo sapiens OX=9606 GN=KEAP1 PE=1 SV=2	69.666	624	62636	6226.4	3.33052
Q9H2D6	TRIOBP	TRIO and F-actin-binding protein OS=Homo sapiens OX=9606 GN=TRIOBP PE=1 SV=3	261.376	2365	16089	1609.7	3.32121
Q9BZI7	UPF3B	Regulator of nonsense transcripts 3B OS=Homo sapiens OX=9606 GN=UPF3B PE=1 SV=1	57.762	483	581520	58836	3.30506
Q96F63	CCDC97	Coiled-coil domain-containing protein 97 OS=Homo sapiens OX=9606 GN=CCDC97 PE=1 SV=1	38.947	343	48900	4976.8	3.29654
Q8IWC1	MAP7D3	MAP7 domain-containing protein 3 OS=Homo sapiens OX=9606 GN=MAP7D3 PE=1 SV=2	98.429	876	221950	23457	3.24214
P07197	NEFM	Neurofilament medium polypeptide OS=Homo sapiens OX=9606 GN=NEFM PE=1 SV=3	102.472	916	233650	24813	3.23518
O43504	LAMTOR5	Ragulator complex protein LAMTOR5 OS=Homo sapiens OX=9606 GN=LAMTOR5 PE=1 SV=1	9.614	91	134770	14437	3.22266
Q8IVM0	CCDC50	Coiled-coil domain-containing protein 50 OS=Homo sapiens OX=9606 GN=CCDC50 PE=1 SV=1	35.822	306	32318	3476.6	3.21659
Q99816	TSG101	Tumor susceptibility gene 101 protein OS=Homo sapiens OX=9606 GN=TSG101 PE=1 SV=2	43.944	390	77568	8596.4	3.17366
O00151	PDLIM1	PDZ and LIM domain protein 1 OS=Homo sapiens OX=9606 GN=PDLIM1 PE=1 SV=4	36.072	329	1522100	168700	3.17353
Q9C0J8	WDR33	pre-mRNA 3' end processing protein WDR33 OS=Homo sapiens OX=9606 GN=WDR33 PE=1 SV=2	145.891	1336	81135	9028.7	3.16773
O75381	PEX14	Peroxisomal membrane protein PEX14 OS=Homo sapiens OX=9606 GN=PEX14 PE=1 SV=1	41.237	377	82245	9186.2	3.16239
O43464	HTRA2	Serine protease HTRA2, mitochondrial OS=Homo sapiens OX=9606 GN=HTRA2 PE=1 SV=2	48.841	458	250420	27978	3.16199
Q9Y6X9	MORC2	ATPase MORC2 OS=Homo sapiens OX=9606 GN=MORC2 PE=1 SV=2	117.823	1032	41862	4733.6	3.14463
Q96RN5	MED15	Mediator of RNA polymerase II transcription subunit 15 OS=Homo sapiens OX=9606 GN=MED15 PE=1 SV=2	86.753	788	100250	11354	3.14233
Q8IV63	VRK3	Inactive serine/threonine-protein kinase VRK3 OS=Homo sapiens OX=9606 GN=VRK3 PE=1 SV=2	52.881	474	50537	5769.1	3.13092
O43747	AP1G1	AP-1 complex subunit gamma-1 OS=Homo sapiens OX=9606 GN=AP1G1 PE=1 SV=5	91.351	822	75731	8684.7	3.12434
P35244	RPA3	Replication protein A 14 kDa subunit OS=Homo sapiens OX=9606 GN=RPA3 PE=1 SV=1	13.569	121	343150	39971	3.10181
Q5T3I0	GPATCH4	G patch domain-containing protein 4 OS=Homo sapiens OX=9606 GN=GPATCH4 PE=1 SV=2	50.381	446	102740	12040	3.09309
Q15554	TERF2	Telomeric repeat-binding factor 2 OS=Homo sapiens OX=9606 GN=TERF2 PE=1 SV=3	59.594	542	79146	9302.8	3.08878
Q9BRS2	RIOK1	Serine/threonine-protein kinase RIO1 OS=Homo sapiens OX=9606 GN=RIOK1 PE=1 SV=2	65.583	568	113160	13592	3.05753
Q15051	IQCB1	IQ calmodulin-binding motif-containing protein 1 OS=Homo sapiens OX=9606 GN=IQCB1 PE=1 SV=1	68.929	598	81760	10041	3.02549
Q15836	VAMP3	Vesicle-associated membrane protein 3 OS=Homo sapiens OX=9606 GN=VAMP3 PE=1 SV=3	11.309	100	16692	2079.2	3.00506

Appendix Table S4. A list of reagents used in the study

Reagent	Supplier	Catalog No.
Human EGF	Peprotech	#100-15
Human IFNγ	Peprotech	#300-02
Mouse IFNγ	Peprotech	#3315-05
Afatinib	Aladdin	#401422
U0126	Aladdin	#U274745
LY294002	Aladdin	#L124970
Saracatinib	Aladdin	#S125141
Ruxolitinib	Aladdin	#R126338
Amuvatinib	Aladdin	#M127412
Decitabine	Aladdin	#408747
Recombinant human GST-SRC protein	SinoBiological	#S19-18G
Recombinant human GST-Ran protein	SinoBiological	#Ag0748
Polybrene	Millipore	#3924803
Hygromycin	InvivoGen	#ant-hg-1
Blasticidin	InvivoGen	#ant-bl-05
SYBR	Bio-Rad	#1725125
Cycloheximide	Sigma	#239763

Appendix Table S5. A list of primary antibodies used in the study

Antibody	Supplier	Catalog No.	Appl.[a]	Usage
Mouse anti-Flag M2 antibody clone M2	Sigma-Aldrich	#F3165	WB/IP	1:2000/1 μg
Mouse anti-HA. 11 Epitope Tag antibody clone 16B12	BioLegend	#901515	WB/IP	1:2000/1 μg
Mouse anti-Myc-Tag antibody clone 9B11	Cell Signaling Technology	#2276S	WB	1:2000
Mouse anti-β-actin antibody clone AC-74	Sigma-Aldrich	#A2228	WB	1:5000
Rabbit anti-GLDC polyclonal antibody	NOVUS	#NBP1-32907	WB/IP/IF	1:1000/1 μg /1:200
Rabbit anti-GLDC polyclonal antibody	GeneTex	#GTX110267	WB/IP	1:1000/1 μg
Rabbit anti-HLA-A antibody clone EP1395Y	Abcam	#ab52922	WB	1:1000
Mouse anti-B2M antibody clone 1C3B7	Proteintech	#66207-1-lg	WB	1:1000
Rabbit anti-EGFR polyclonal antibody	Proteintech	#18986-1-AP	WB	1:2000
Rabbit anti-STAT1 antibody clone D1K9Y	Cell Signaling Technology	#14994	WB/ChIP	1:1000/1:200
Rabbit anti-pSTAT1 (Tyr701) antibody clone 58D6	Cell Signaling Technology	#9167	WB	1:1000
Rabbit anti-pSTAT1 (Ser727) antibody clone D3B7	Cell Signaling Technology	#8826	WB	1:1000
Rabbit anti-p65 antibody clone D3B7	Cell Signaling Technology	#8242	WB	1:20000
Mouse anti-β-tubulin polyclonal antibody	ABclonal	#AC021	WB	1:5000
Rabbit anti-Lamin B1 polyclonal antibody	Proteintech	#12987-1-AP	WB	1:7500
Rabbit anti-p-Ser/Thr polyclonal antibody	Abcam	#ab17464	WB	1:1000

Mouse anti-p-Tyr antibody clone P-Tyr-100	Cell Signaling Technology	#9411	WB	1:2000
Rabbit anti-p-Tyr antibody clone P-Tyr-1000	Cell Signaling Technology	#8954	WB	1:2000
Rabbit anti-pSRC (Tyr419) polyclonal antibody	Abcam	#ab4816	WB	1:1000
Rabbit anti-SRC antibody clone 36D10	Cell Signaling Technology	#2109	WB	1:1000
Rabbit anti-Ran polyclonal antibody	Proteintech	#10469-1-AP	WB	1:1500
Rabbit anti-SMARCE1 polyclonal antibody	FORTIS	#A300-810A	WB/IP /ChIP	1:1500/2 μg /2 μg
Rabbit anti-DMAP1 polyclonal antibody	Proteintech	#10411-1-AP	WB/IP	1:1500/2 μg
Rabbit anti-DNMT1 antibody clone D63A6	Cell Signaling Technology	#5032	WB	1:1000
Mouse anti-5mC antibody clone 33D3	Abcam	#ab10805	MeDIP	1 μg
Rabbit anti-ARID1A antibody clone D2A8U	Cell Signaling Technology	#12354	WB	1:1000
Rabbit anti-ARID2 antibody clone D8D8U	Cell Signaling Technology	#82342	WB	1:1000
Rabbit anti-SMARCC1 antibody clone D7F8S	Cell Signaling Technology	#11956	WB	1:1000

Appendix Table S6. A list of flow antibodies used in the study

Antibody	Clone	Supplier	Catalog No.
FITC anti-human HLA-ABC antibody	W6/32	BioLegend	#311404
APC anti-human HLA-ABC antibody	W6/32	BioLegend	#311410
PE anti-human B2M antibody	TU99	BD Biosciences	#551337
Purified anti-human B2M antibody	2M2	BioLegend	#316302
APC anti-mouse H-2$K^{d/}D^{d}$ antibody	34-1-2S	BioLegend	#114714
APC anti-mouse H-2$K^{b/}D^{b}$ antibody	28-8-6	BioLegend	#114614
APC anti-mouse H-2K^{b} bound to SIINFEKL antibody	25-D1.16	BioLegend	#141606
FITC anti-mouse CD45.2 antibody	104	BioLegend	109806
PE-Cy7 anti-mouse CD3 antibody	145-2C11	BD Biosciences	#552774
PB anti-mouse CD8 antibody	53-6.7	BD Biosciences	#558106
PerCP-710 anti-mouse GzmB antibody	NGZB	eBioscience	#46-8898-82
APC anti-mouse IFNγ antibody	XMG1.2	eBioscience	#17-7311-82

Appendix Table S7. A list of gRNA sequences

Human *GLDC*	5'-CAGATCTGGAGATCGTATAT-3'
Human *Ran*	5'-GCCGGCCAGGAGAAATTCGG-3'
Human *SMARCE1*	5'-TTATGTAAGCAAGGTACGCG-3'
Human *STAT1* #1	5'-TGTGATAGGGTCATGTTCGT-3'
Human *STAT1* #2	5'-ATTGGGCGGCCCCCCAATAC-3'
Human *p65* #1	5'-AGCGCCCCTCGCACTTGTAG-3'
Human *p65* #2	5'-GCTTCCGCTACAAGTGCGAG-3'
Human *SRC* #1	5'-GGCTTGCTGGGGGTCTGCGAGG-3'
Human *SRC* #2	5'-AGCGCCGTGCACGTTCTCGG-3'
Human *DNMT1* #1	5'-CGCTGCCCGACGATGTCCGC-3'
Human *DNMT1* #2	5'-GGTACGCGCCGGCATCTCGG-3'
Human *DMAP1* #1	5'-CGTTAGGAACATCCAAGCGG-3'
Human *DMAP1* #2	5'-AACTCGCCTCCGCTTAGGTC-3'
Mouse *Gldc*	5'-TCCCGGAGACACATCGGCCC-3'

Appendix Table S8. A list of qPCR sequences

Human *GAPDH*	GTCTCCTCTGACTTCAACAGCG
	ACCACCCTGTTGCTGTAGCCAA
Human *β-actin*	GCACAGAGCCTCGCCTT
	CCTTGCACATGCCGGAG
Human *HLA-A*	AAAAGGAGGGAGTTACACTCAGG
	GCTGTGAGGGACACATCAGAG
Human *HLA-B*	CAGTTCGTGAGGTTCGACAG
	CAGCCGTACATGCTCTGGA
Human *HLA-C*	CACACCTCTCCTTTGTGACTTCAA
	CCACCTCCTCACATTATGCTAACA
Human *B2M*	GAGGCTATCCAGCGTACTCCA
	CGGCAGGCATACTCATCTTTT
Human *TAP1*	TGCCCCGCATATTCTCCCT
	CACCTGCGTTTTCGCTCTTG
Human *TAP2*	TGGACGCGGCTTTACTGTG
	GCAGCCCTCTTAGCTTTAGCA
Human *TAPBP*	CCTGGAGGTAGCAGGTCTTTC
	ATCCTTGCAGGTGGACAGGTA
Human *ERAP1*	GCAAACCTTACCACGCTGAC
	GGTTCTTCCGATAGCCTCTCTC
Human *ERAP2*	CACTAATGGGGAACGATTTCCTT
	CTGACCAAGACTTCGATCTTCTC
Human *PSMB9*	GGTTCTGATTCCCGAGTGTCT
	CAGCCAAAACAAGTGGAGGTT
Human *PSMB10*	TCCTTCGAGAACTGCCAAAGA
	ATCGTTAGTGGCTCGCGTATC
Human *IRF1*	GAGGAGGTGAAAGACCAGAGCA
	TAGCATCTCGGCTGGACTTCGA
Human *NLRC5*	AGTGGCTCTTCCGCTTGGACAT
	CGGAACCCTAAGAACTTGGCTG
Human *STAT1*	ATGGCAGTCTGGCGGCTGAATT
	CCAAACCAGGCTGGCACAATTG
Mouse *Gapdh*	ACGGCCGCATCTTCTTGTGCA
	ACGGCCAAATCCGTTCACACC
Mouse *H2-K1*	GCTGGTGAAGCAGAGAGACTCAG
	GGTGACTTTATCTTCAGGTCTGCT

Mouse *H2-D1*	AGTGGTGCTGCAGAGCATTACAA
	GGTGACTTCACCTTTAGATCTGGG
Mouse *B2m*	TGGTGCTTGTCTCACTGACC
	TTCAGTATGTTCGGCTTCCC
Mouse *Tap1*	AGTCTGGAGCCCACGATTTCATC
	GGGTGATAAGAAGAACCGTCCG
Mouse *Tapbp*	GGCCTGTCTAAGAAACCTGCC
	CCACCTTGAAGTATAGCTTTGGG
Mouse *Erap1*	TAATGGAGACTCATTCCCTTGGA
	AAAGTCAGAGTGCTGAGGTTTG
Mouse *Irf1*	TCCAAGTCCAGCCGAGACACTA
	ACTGCTGTGGTCATCAGGTAGG
Mouse *Nlrc5*	TCAGCCCAGAACAAGTATCC
	TGGGCACAGACTTCCATTAG

Appendix Table S9. A list of qPCR sequences for CHIP/MeDIP analysis

Human *IRF1 #1*	TACTTCCCCTTCGCCGCTA
	CGTCTTGCCTCGACTAAGGAG
Human *IRF1 #2*	CCCTGTACTTCCCCTTCGCC
	GCCACCGAGCAATCCAAACA
Human *NLRC5 #1*	GACTGTTTTTCCCGGGTCAG
	TGAAGGTCGGCTCACATGC
Human *NLRC5 #2*	TGTTTTTCCCGGGTCAGCC
	GTGAAGGTCGGCTCACATGC
Mouse *Irf1 #1*	TCGCCGCTTAGCTCTACAAC
	TGAAAGCACGTCCTACCTCG
Mouse *Irf1 #2*	TGCCTTGTACTTCCCCTTCG
	GGCGCCGCGAAGAAATCTAA
