# Supplementary figures and images for: Nuclear glycine decarboxylase suppresses STAT1-dependent MHC-I and promotes cancer immune evasion (part 1 of 2)
Source: EMBO J. 2025 Sep 8;44(20):5712–33. doi: 10.1038/s44318-025-00557-3 (PMC12528744; doi:10.1038/s44318-025-00557-3)

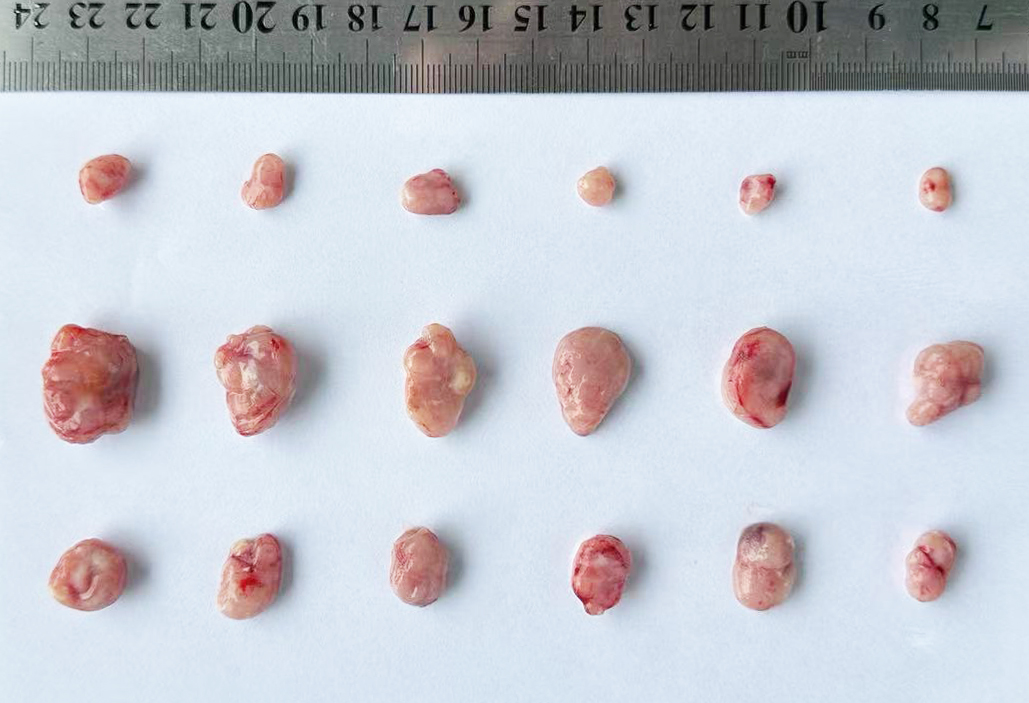

Supplement: Supplementary file 4 — Source data Fig. 2 [file 44318_2025_557_MOESM4_ESM.zip › Figure 2/2G/Figure 2G---Tumor image.jpg]

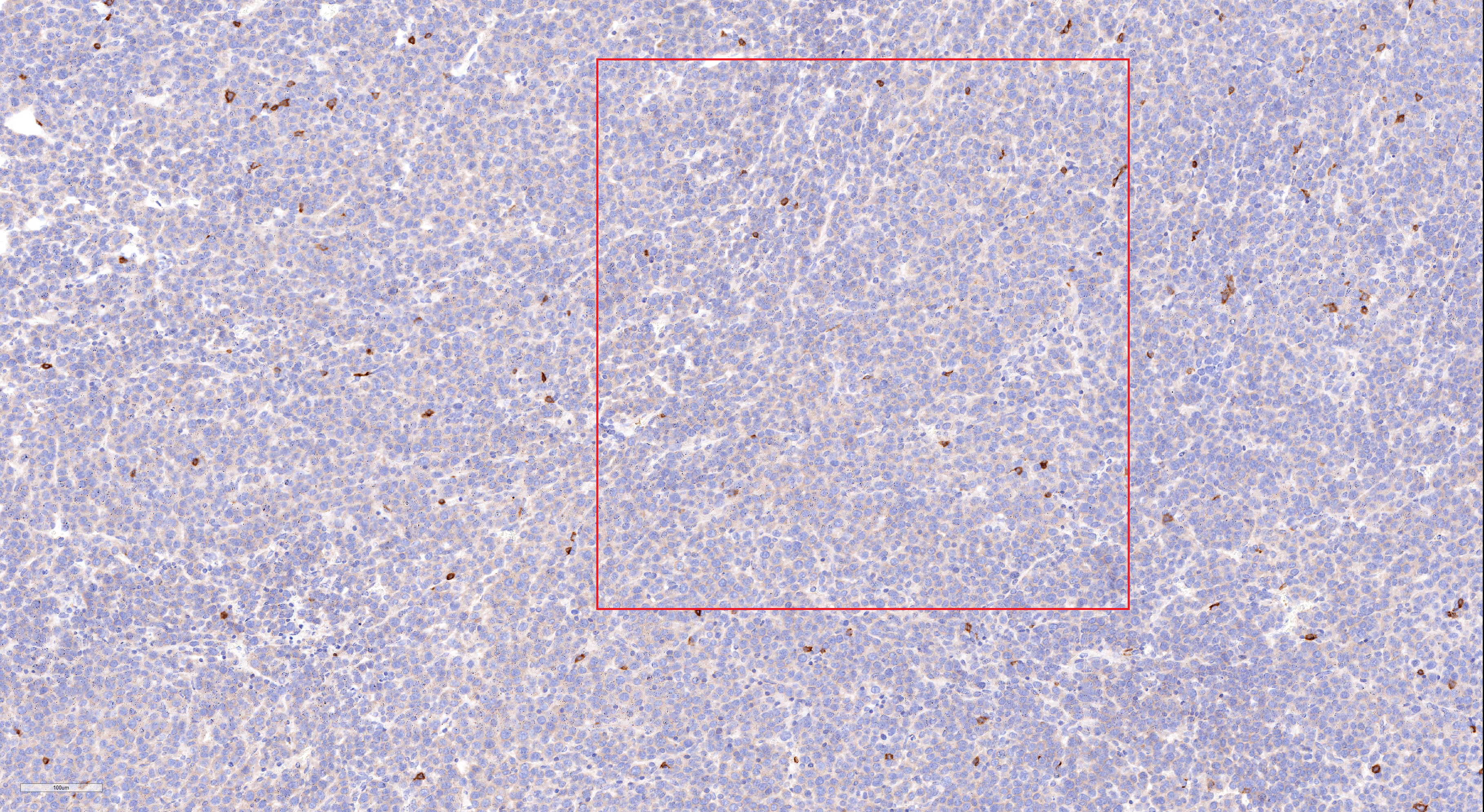

Supplement: Supplementary file 4 — Source data Fig. 2 [file 44318_2025_557_MOESM4_ESM.zip › Figure 2/2I/Figure 2I---IHC-CD8-gGldc-GldcGE.tif]

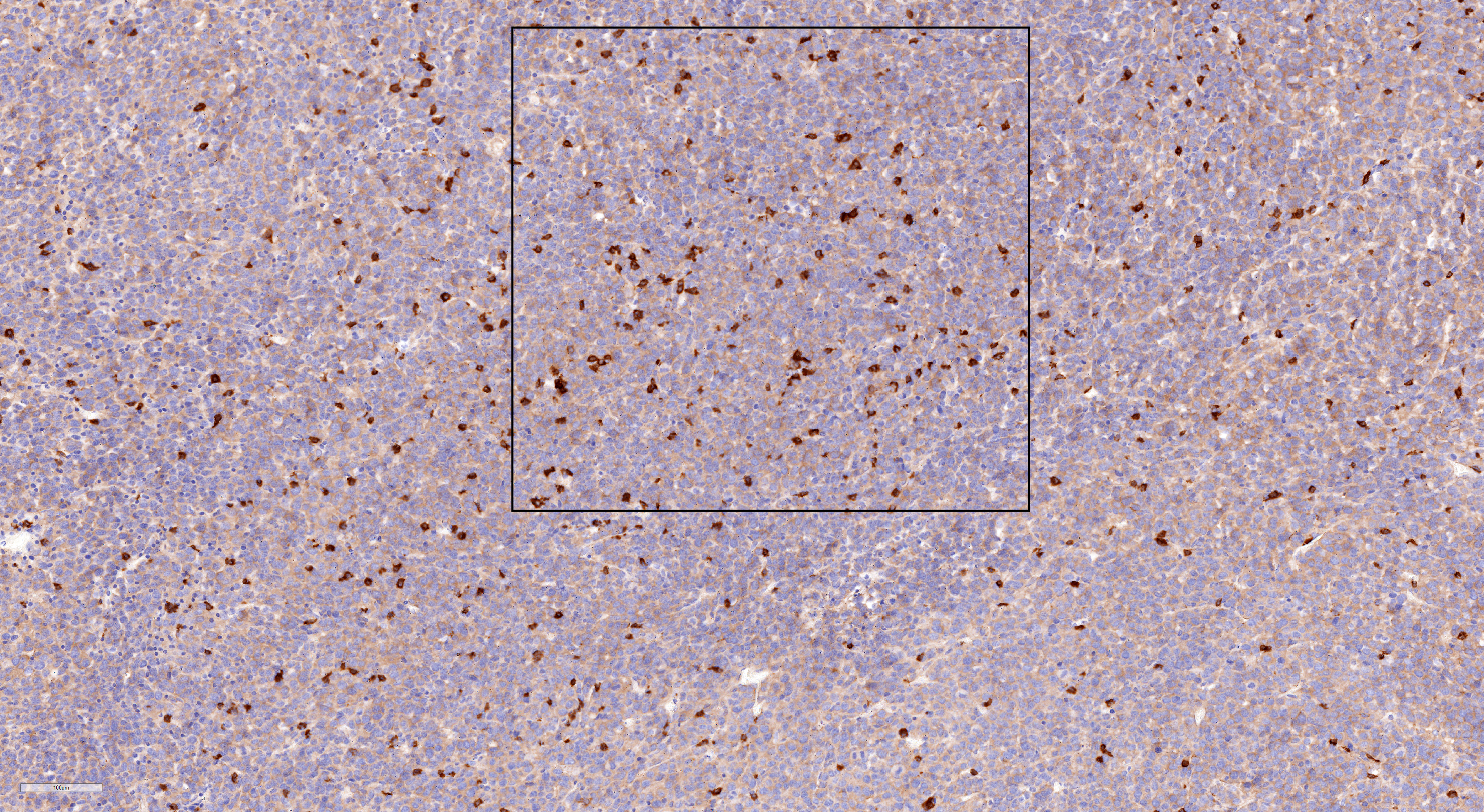

Supplement: Supplementary file 4 — Source data Fig. 2 [file 44318_2025_557_MOESM4_ESM.zip › Figure 2/2I/Figure 2I---IHC-CD8-gGldc.tif]

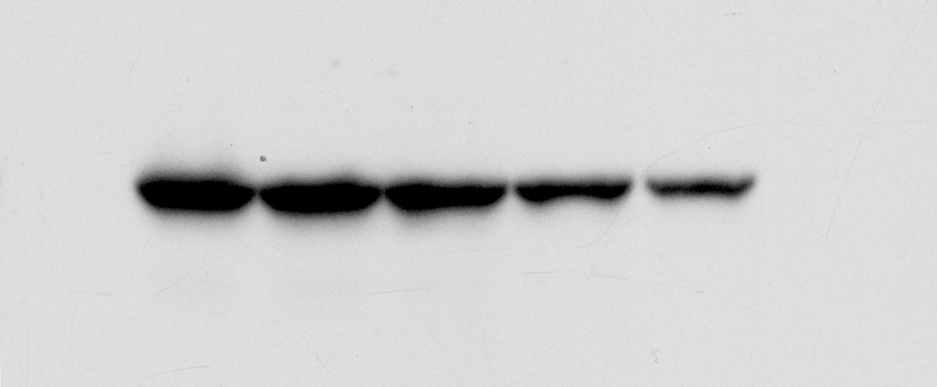

Supplement: Supplementary file 5 — Source data Fig. 3 [file 44318_2025_557_MOESM5_ESM.zip › Figure 3/3C/Figure 3C---gGLDC--Western HLA-A.jpg]

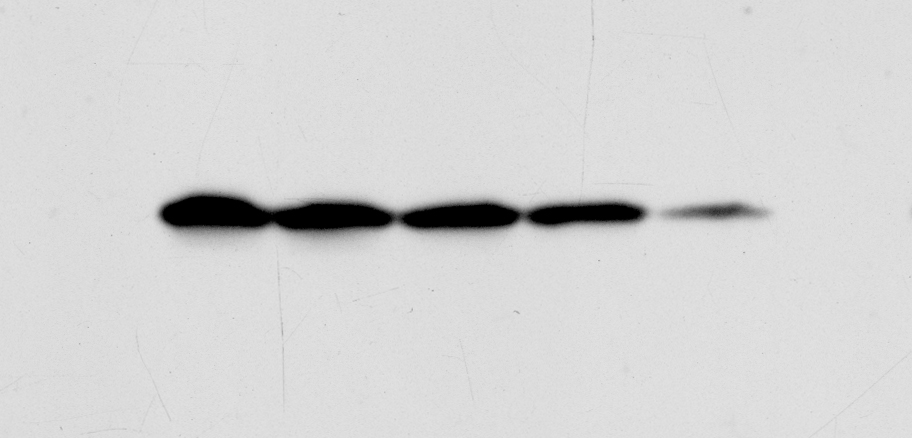

Supplement: Supplementary file 5 — Source data Fig. 3 [file 44318_2025_557_MOESM5_ESM.zip › Figure 3/3C/Figure 3C---gGLDC--Western B2M.jpg]

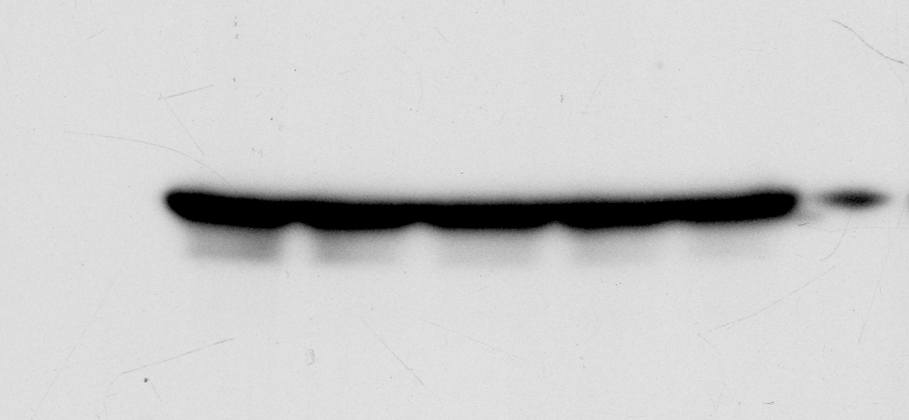

Supplement: Supplementary file 5 — Source data Fig. 3 [file 44318_2025_557_MOESM5_ESM.zip › Figure 3/3C/Figure 3C---gNC--Western actin.jpg]

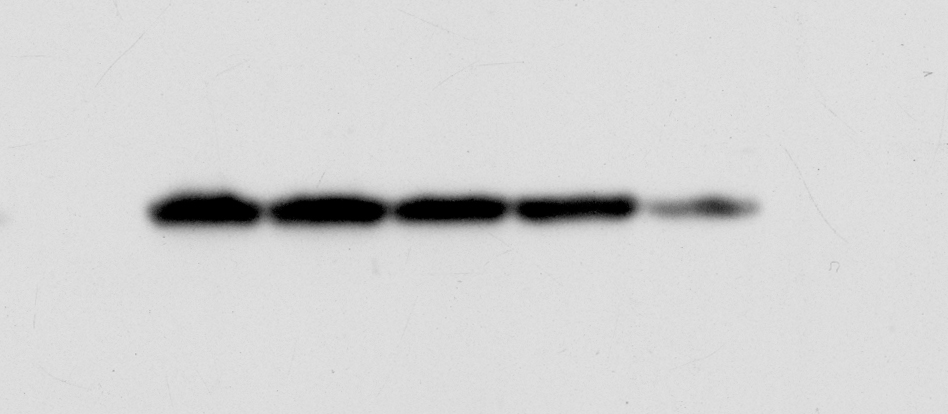

Supplement: Supplementary file 5 — Source data Fig. 3 [file 44318_2025_557_MOESM5_ESM.zip › Figure 3/3C/Figure 3C---gNC--Western B2M.jpg]

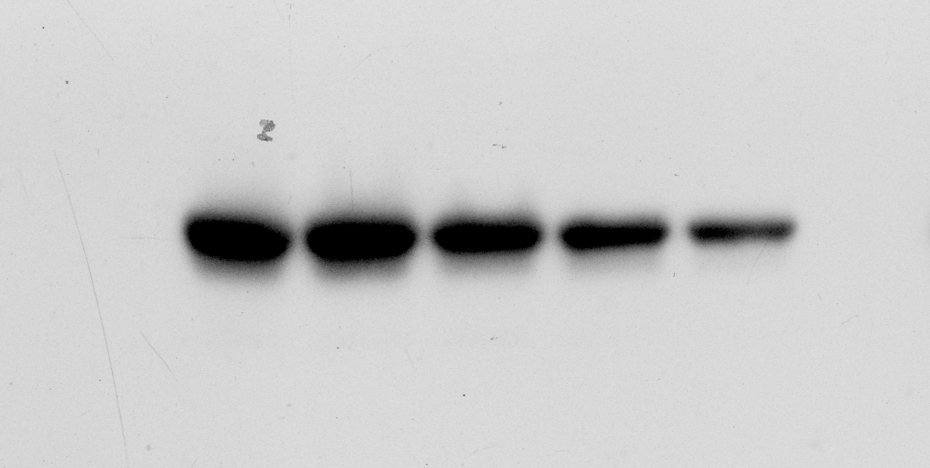

Supplement: Supplementary file 5 — Source data Fig. 3 [file 44318_2025_557_MOESM5_ESM.zip › Figure 3/3C/Figure 3C---gNC--Western HLA-A.jpg]

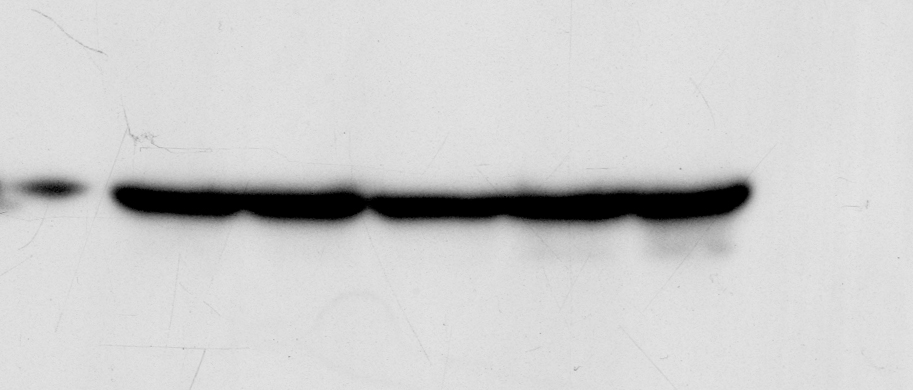

Supplement: Supplementary file 5 — Source data Fig. 3 [file 44318_2025_557_MOESM5_ESM.zip › Figure 3/3C/Figure 3C---gGLDC--Western actin.jpg]

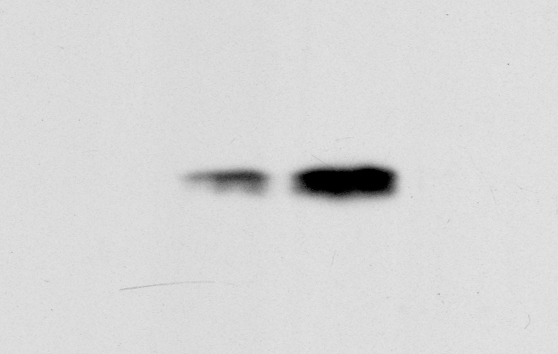

Supplement: Supplementary file 5 — Source data Fig. 3 [file 44318_2025_557_MOESM5_ESM.zip › Figure 3/3A/Figure 3A---Western B2M.jpg]

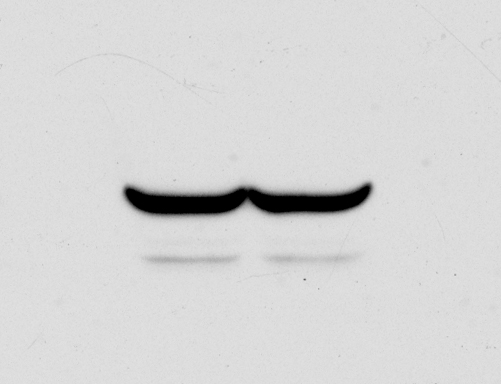

Supplement: Supplementary file 5 — Source data Fig. 3 [file 44318_2025_557_MOESM5_ESM.zip › Figure 3/3A/Figure 3A---Western actin.jpg]

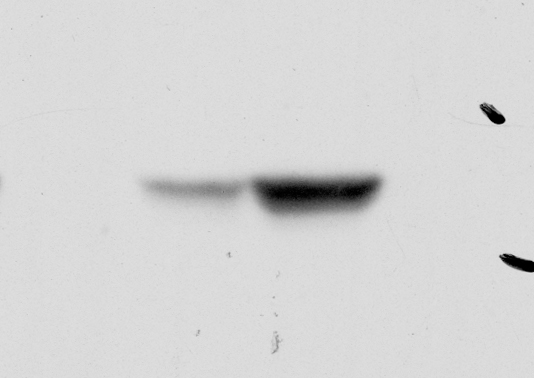

Supplement: Supplementary file 5 — Source data Fig. 3 [file 44318_2025_557_MOESM5_ESM.zip › Figure 3/3A/Figure 3A---Western HLA-A.jpg]

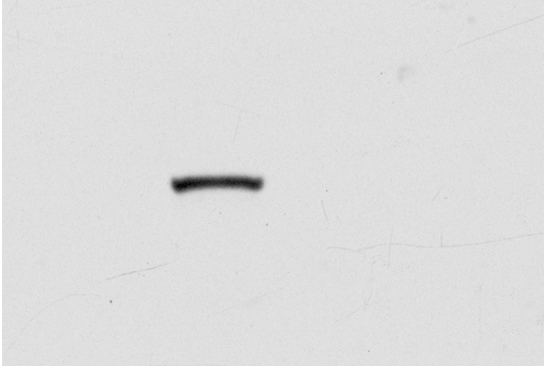

Supplement: Supplementary file 5 — Source data Fig. 3 [file 44318_2025_557_MOESM5_ESM.zip › Figure 3/3A/Figure 3A---Western GLDC.jpg]

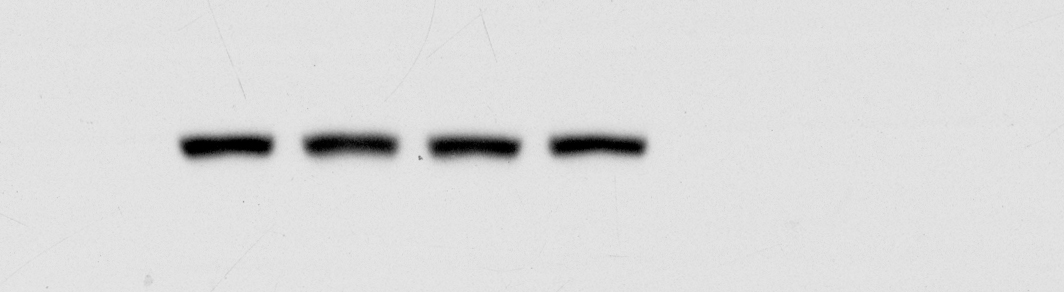

Supplement: Supplementary file 5 — Source data Fig. 3 [file 44318_2025_557_MOESM5_ESM.zip › Figure 3/3I/Figure3I---western p65.jpg]

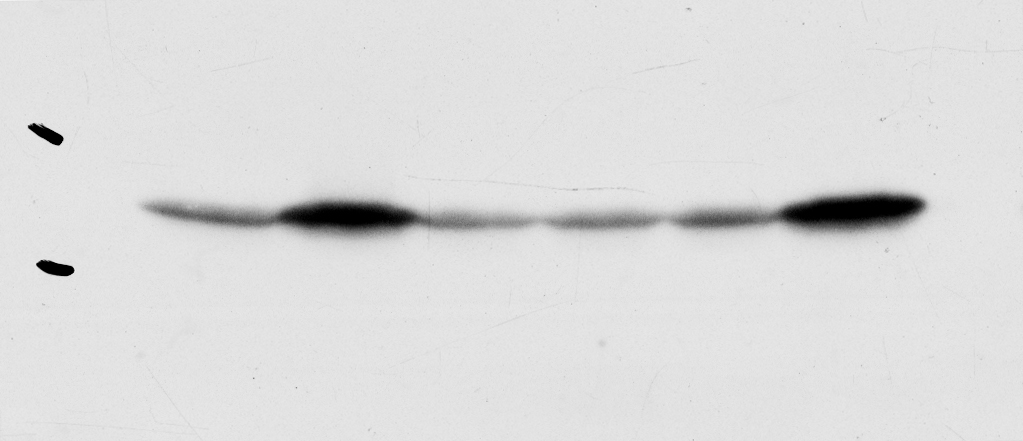

Supplement: Supplementary file 5 — Source data Fig. 3 [file 44318_2025_557_MOESM5_ESM.zip › Figure 3/3I/Figure3I---western HLA-A.jpg]

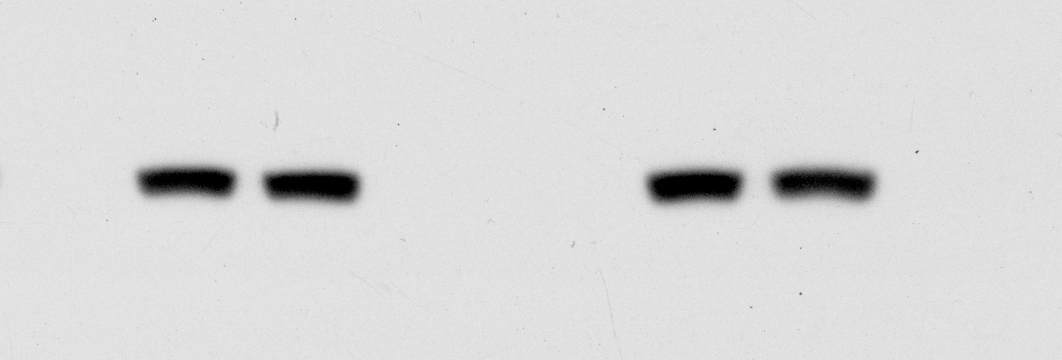

Supplement: Supplementary file 5 — Source data Fig. 3 [file 44318_2025_557_MOESM5_ESM.zip › Figure 3/3I/Figure3I---western STAT1.jpg]

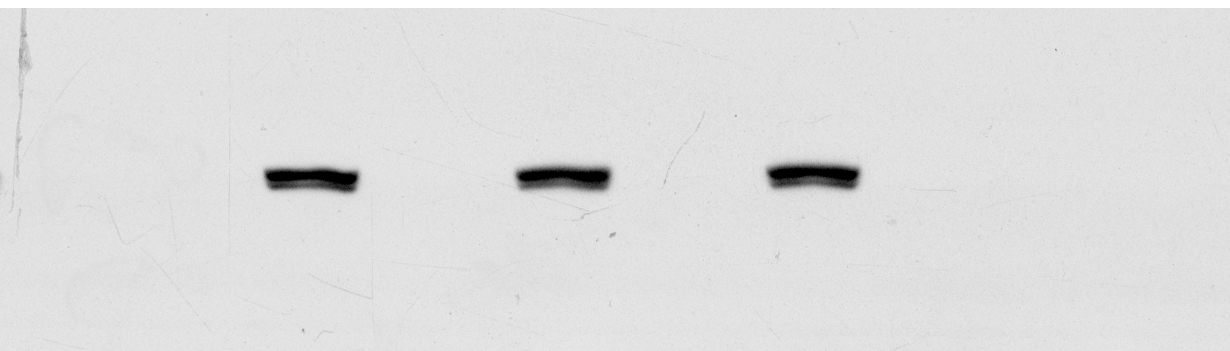

Supplement: Supplementary file 5 — Source data Fig. 3 [file 44318_2025_557_MOESM5_ESM.zip › Figure 3/3I/Figure3I---western GLDC.jpg]

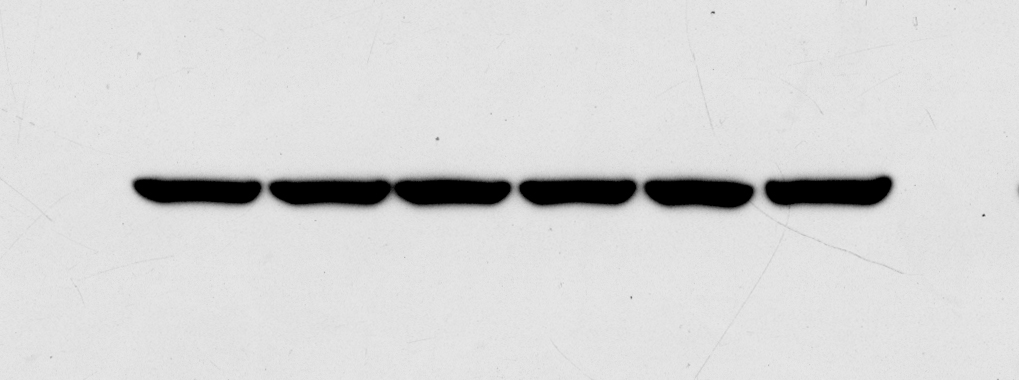

Supplement: Supplementary file 5 — Source data Fig. 3 [file 44318_2025_557_MOESM5_ESM.zip › Figure 3/3I/Figure3I---western actin.jpg]

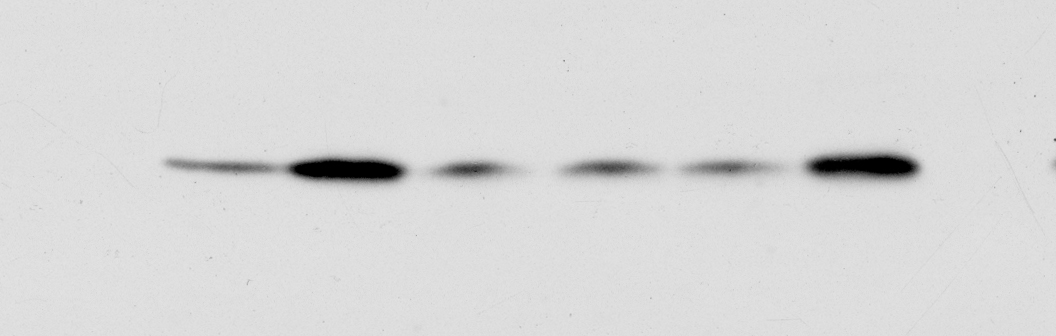

Supplement: Supplementary file 5 — Source data Fig. 3 [file 44318_2025_557_MOESM5_ESM.zip › Figure 3/3I/Figure3I---western B2M.jpg]

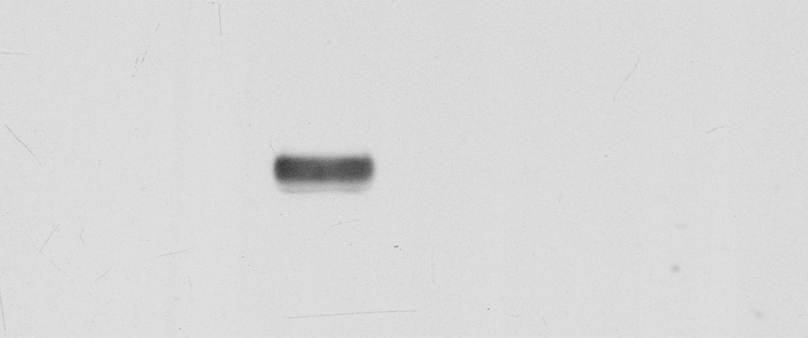

Supplement: Supplementary file 6 — Source data Fig. 4 [file 44318_2025_557_MOESM6_ESM.zip › Figure 4/4E/Figure 4E---IP-p-Tyr.jpg]

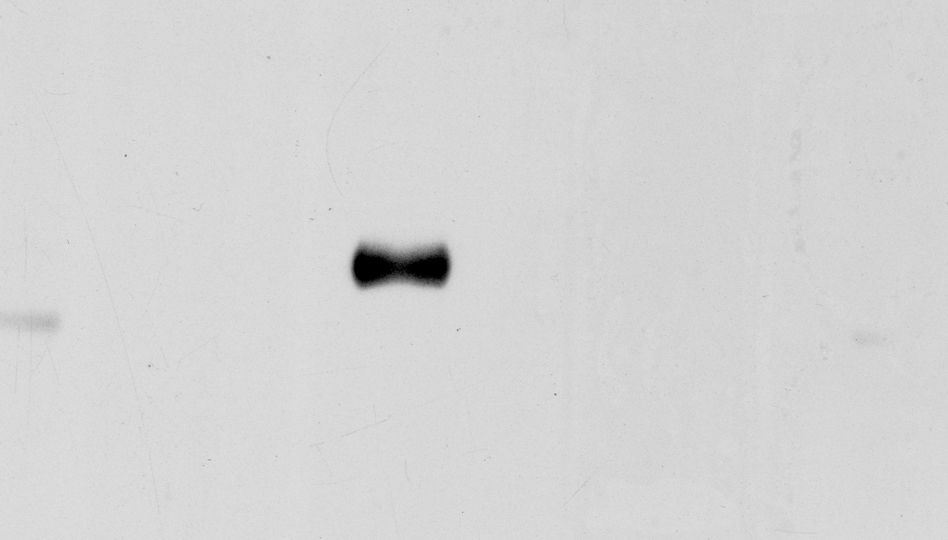

Supplement: Supplementary file 6 — Source data Fig. 4 [file 44318_2025_557_MOESM6_ESM.zip › Figure 4/4E/Figure 4E---IP-pGLDCY993.jpg]

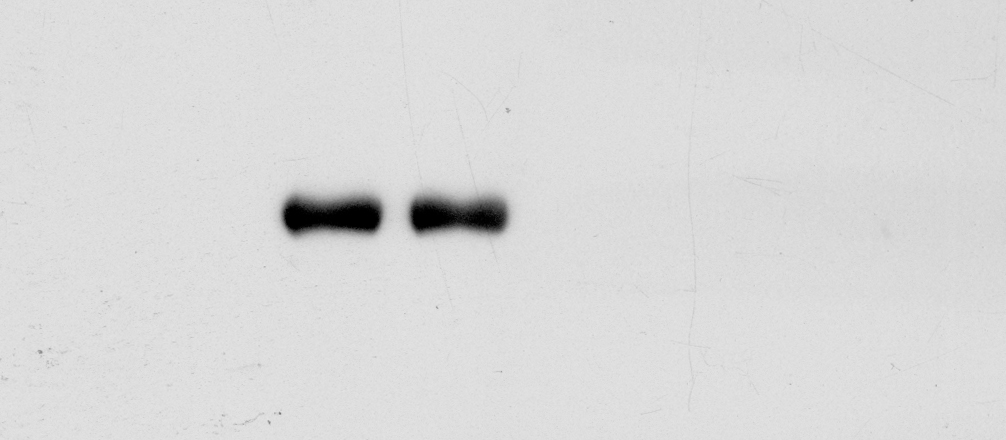

Supplement: Supplementary file 6 — Source data Fig. 4 [file 44318_2025_557_MOESM6_ESM.zip › Figure 4/4E/Figure 4E---IP-GLDC.jpg]

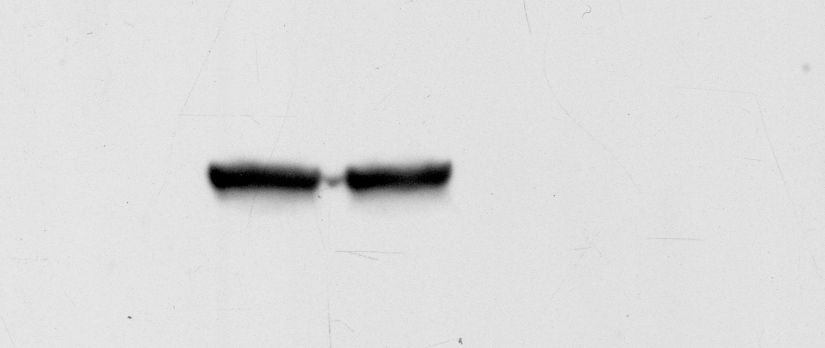

Supplement: Supplementary file 6 — Source data Fig. 4 [file 44318_2025_557_MOESM6_ESM.zip › Figure 4/4E/Figure 4E---Lysate-GLDC.jpg]

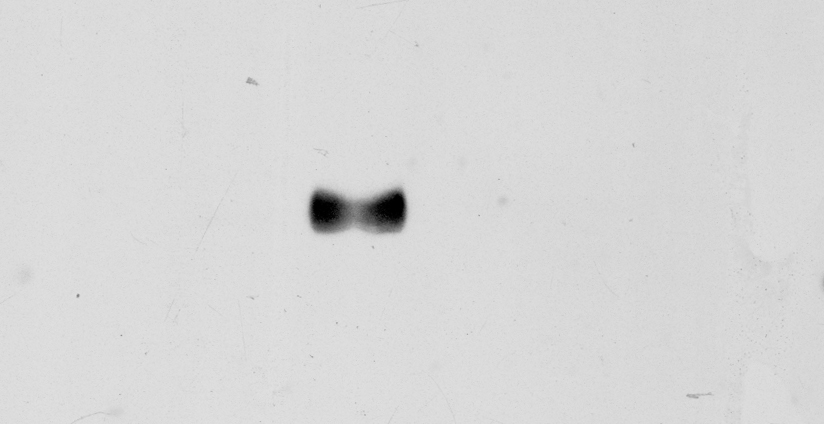

Supplement: Supplementary file 6 — Source data Fig. 4 [file 44318_2025_557_MOESM6_ESM.zip › Figure 4/4E/Figure 4E---IP-pGLDCY1008.jpg]

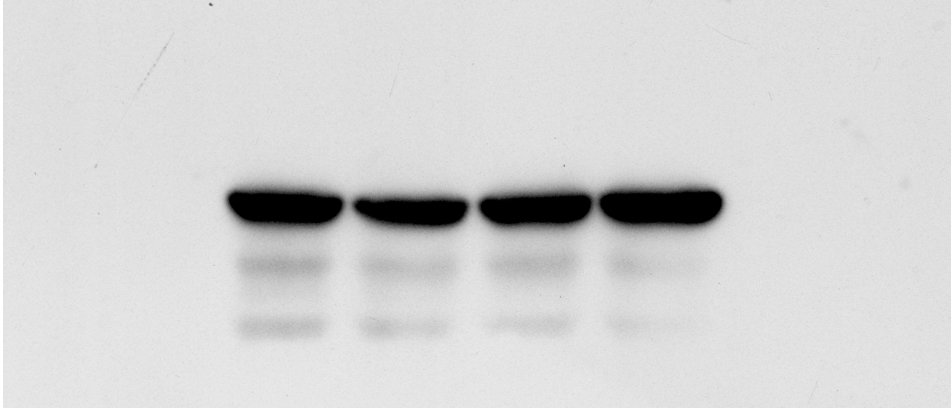

Supplement: Supplementary file 6 — Source data Fig. 4 [file 44318_2025_557_MOESM6_ESM.zip › Figure 4/4E/Figure 4E---Lysate-actin.jpg]

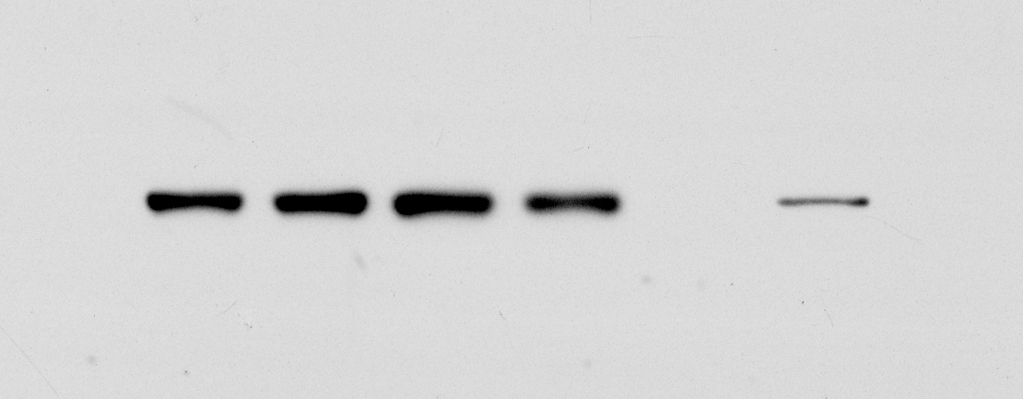

Supplement: Supplementary file 6 — Source data Fig. 4 [file 44318_2025_557_MOESM6_ESM.zip › Figure 4/4B/Figure 4B---A549-GLDC-SE.jpg]

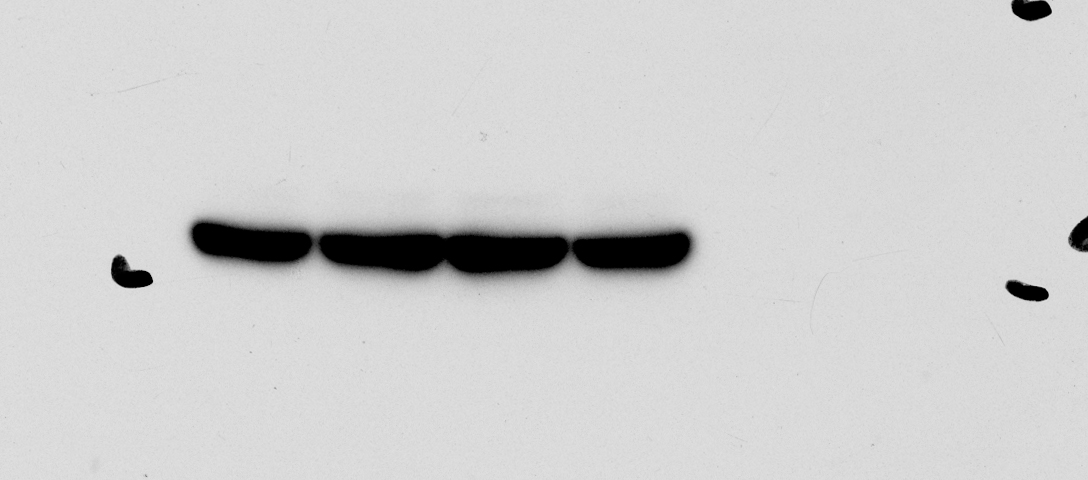

Supplement: Supplementary file 6 — Source data Fig. 4 [file 44318_2025_557_MOESM6_ESM.zip › Figure 4/4B/Figure 4B---H1299-tubulin.jpg]

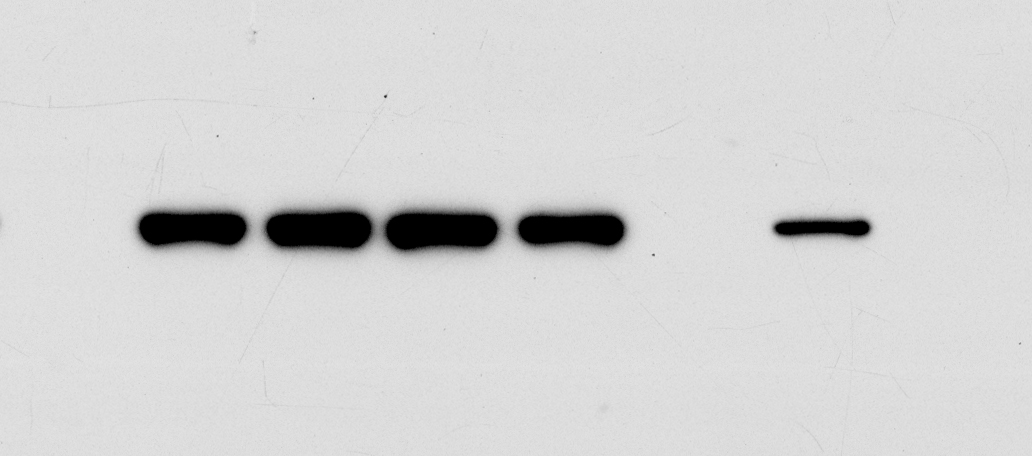

Supplement: Supplementary file 6 — Source data Fig. 4 [file 44318_2025_557_MOESM6_ESM.zip › Figure 4/4B/Figure 4B---A549-GLDC-LE.jpg]

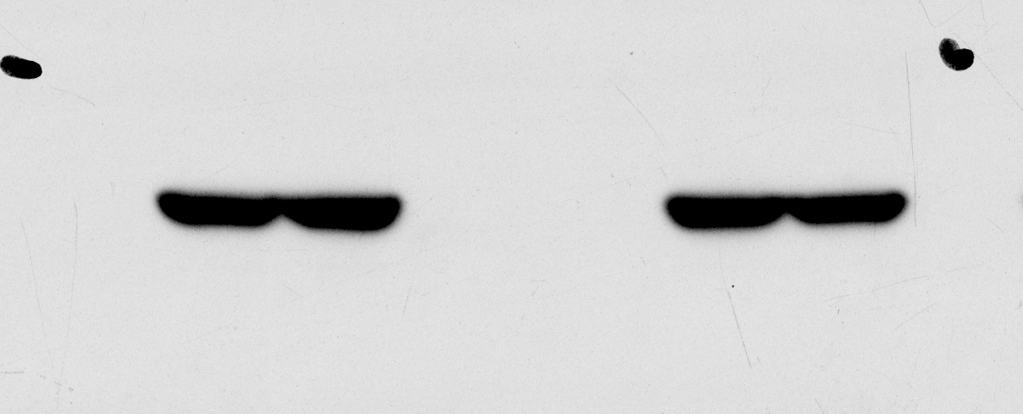

Supplement: Supplementary file 6 — Source data Fig. 4 [file 44318_2025_557_MOESM6_ESM.zip › Figure 4/4B/Figure 4B---A549-Lamin B1.jpg]

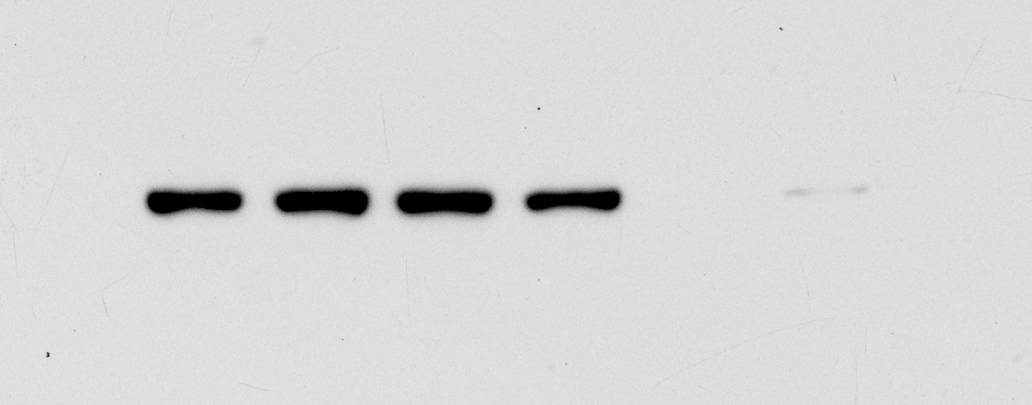

Supplement: Supplementary file 6 — Source data Fig. 4 [file 44318_2025_557_MOESM6_ESM.zip › Figure 4/4B/Figure 4B---H1299-GLDC-SE.jpg]

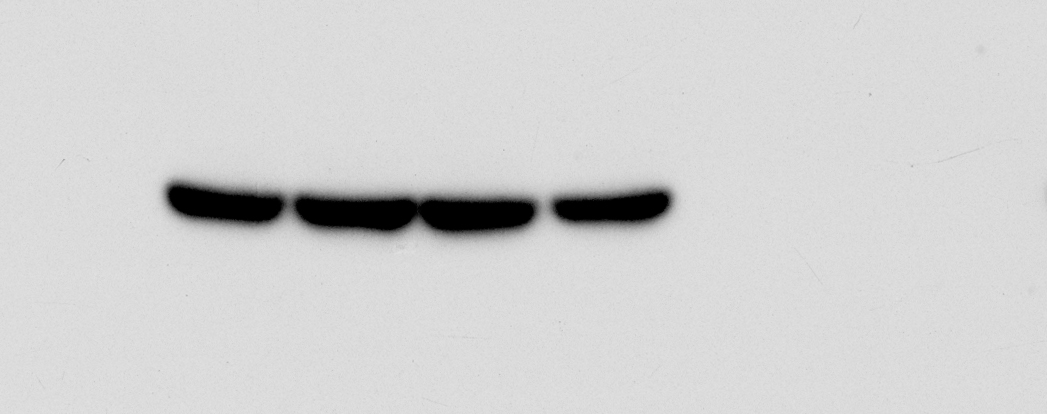

Supplement: Supplementary file 6 — Source data Fig. 4 [file 44318_2025_557_MOESM6_ESM.zip › Figure 4/4B/Figure 4B---A549-tubulin.jpg]

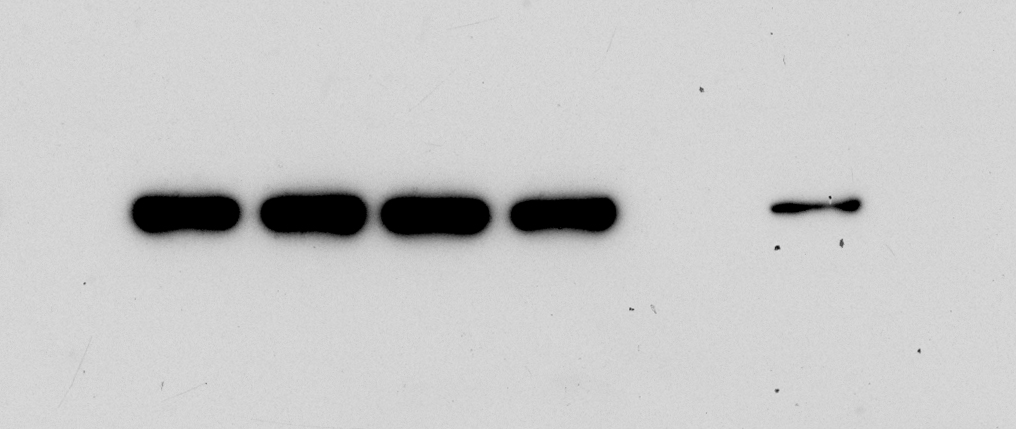

Supplement: Supplementary file 6 — Source data Fig. 4 [file 44318_2025_557_MOESM6_ESM.zip › Figure 4/4B/Figure 4B---H1299-GLDC-LE.jpg]

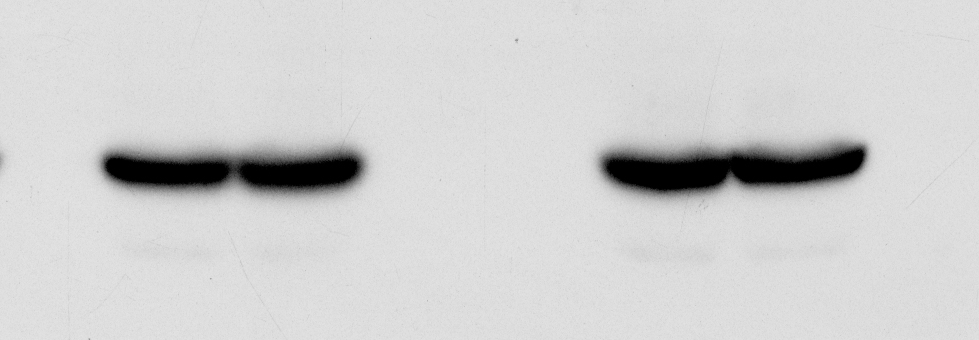

Supplement: Supplementary file 6 — Source data Fig. 4 [file 44318_2025_557_MOESM6_ESM.zip › Figure 4/4B/Figure 4B---H1299-Lamin B1.jpg]

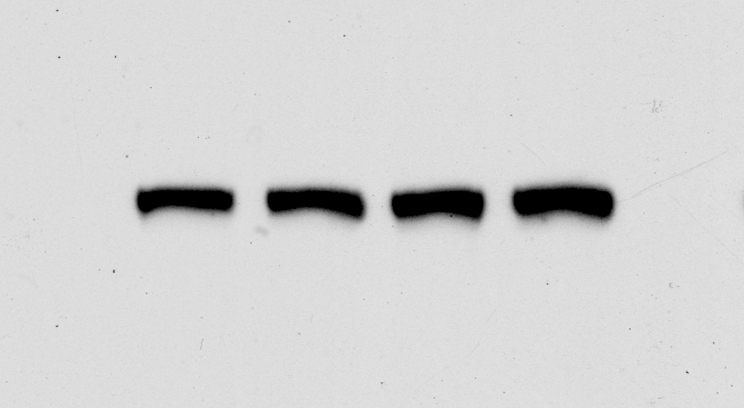

Supplement: Supplementary file 6 — Source data Fig. 4 [file 44318_2025_557_MOESM6_ESM.zip › Figure 4/4K/Figure 4K---WCL-GLDC.jpg]

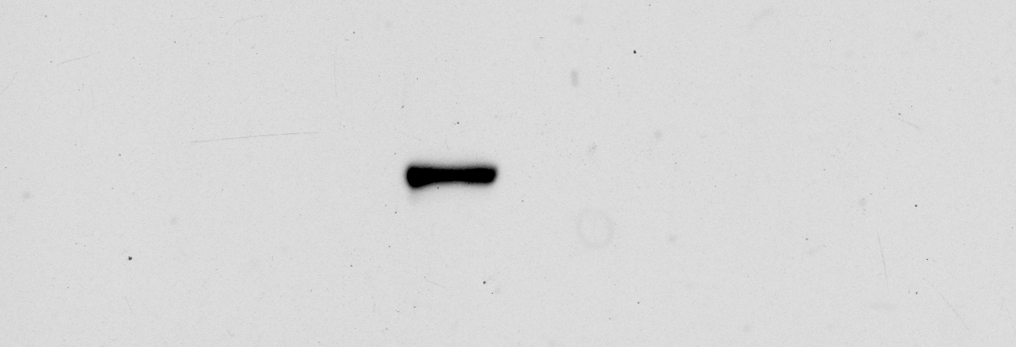

Supplement: Supplementary file 6 — Source data Fig. 4 [file 44318_2025_557_MOESM6_ESM.zip › Figure 4/4K/Figure 4K---Nuclear-GLDC.jpg]

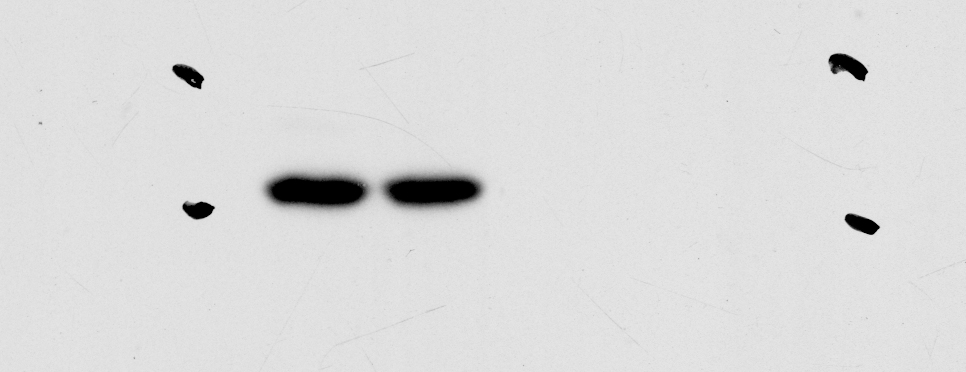

Supplement: Supplementary file 6 — Source data Fig. 4 [file 44318_2025_557_MOESM6_ESM.zip › Figure 4/4K/Figure 4K---WCL-Ran.jpg]

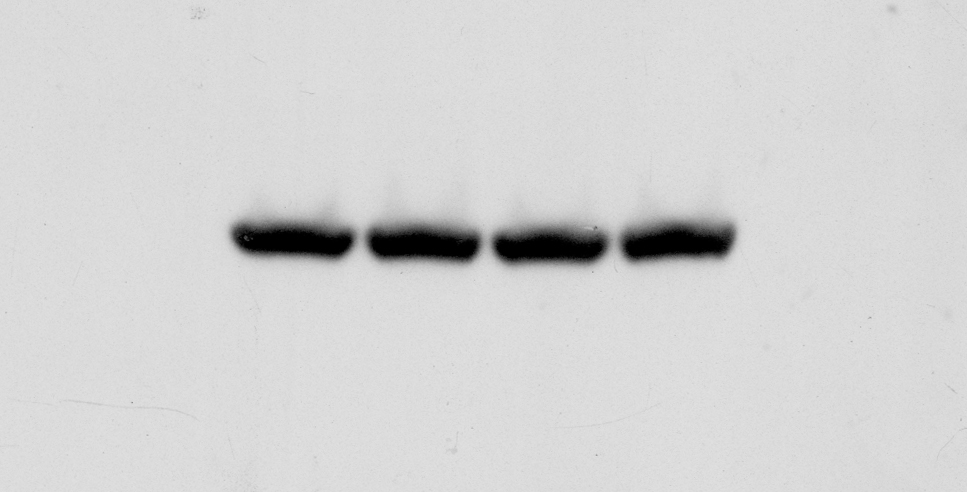

Supplement: Supplementary file 6 — Source data Fig. 4 [file 44318_2025_557_MOESM6_ESM.zip › Figure 4/4K/Figure 4K---Nuclear-Lamin B1.jpg]

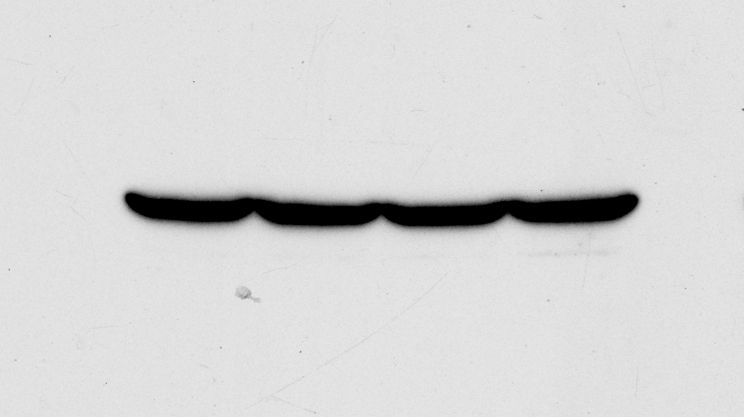

Supplement: Supplementary file 6 — Source data Fig. 4 [file 44318_2025_557_MOESM6_ESM.zip › Figure 4/4K/Figure 4K---WCL-actin.jpg]

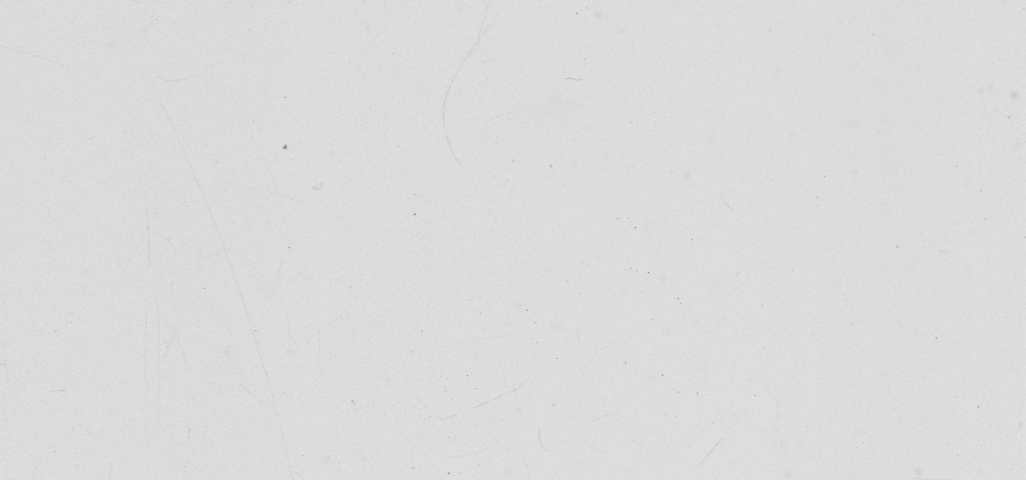

Supplement: Supplementary file 6 — Source data Fig. 4 [file 44318_2025_557_MOESM6_ESM.zip › Figure 4/4K/Figure 4K---Nuclear-tubulin.jpg]

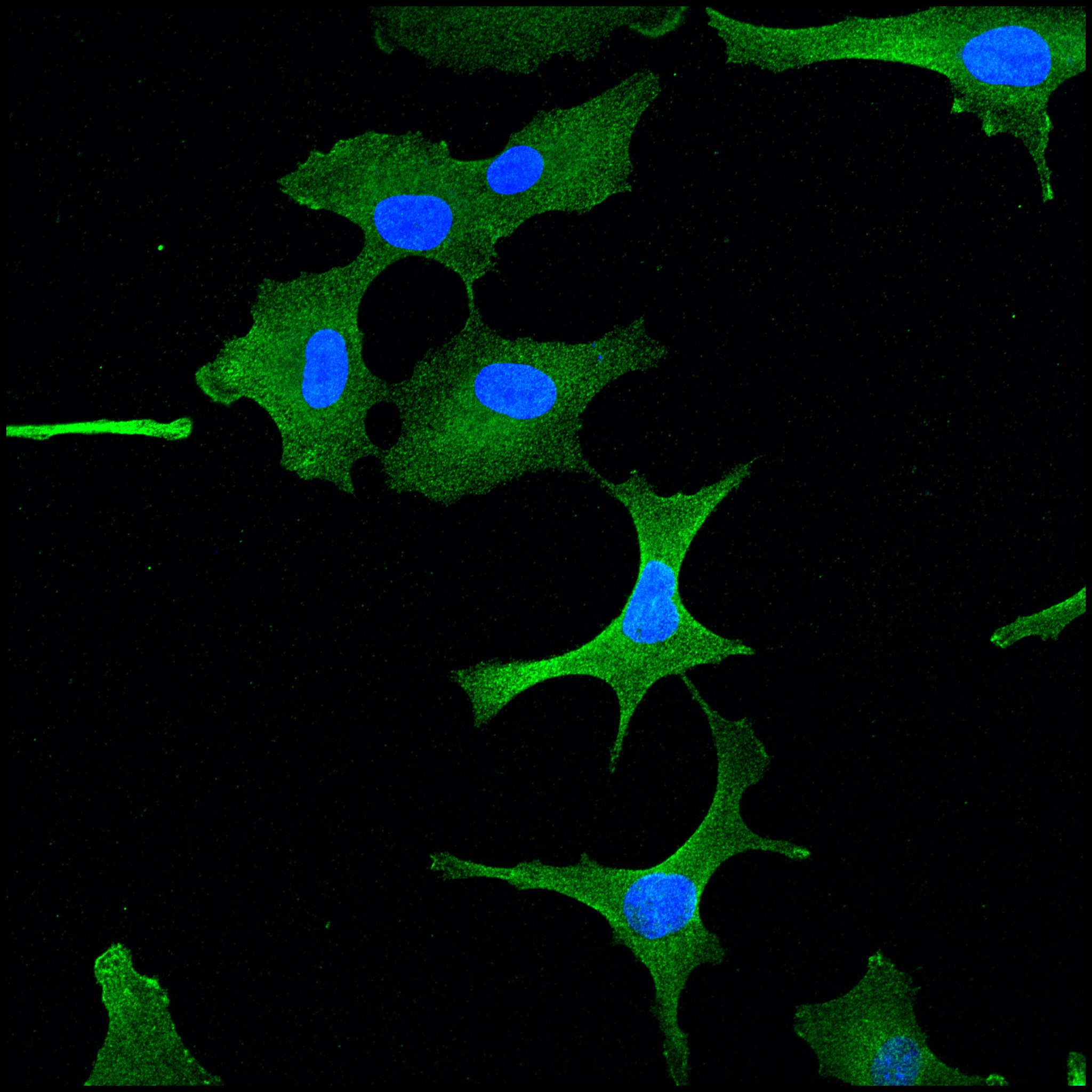

Supplement: Supplementary file 6 — Source data Fig. 4 [file 44318_2025_557_MOESM6_ESM.zip › Figure 4/4L/Figure 4L---WT-Flag-DAPI-Merge.jpg]

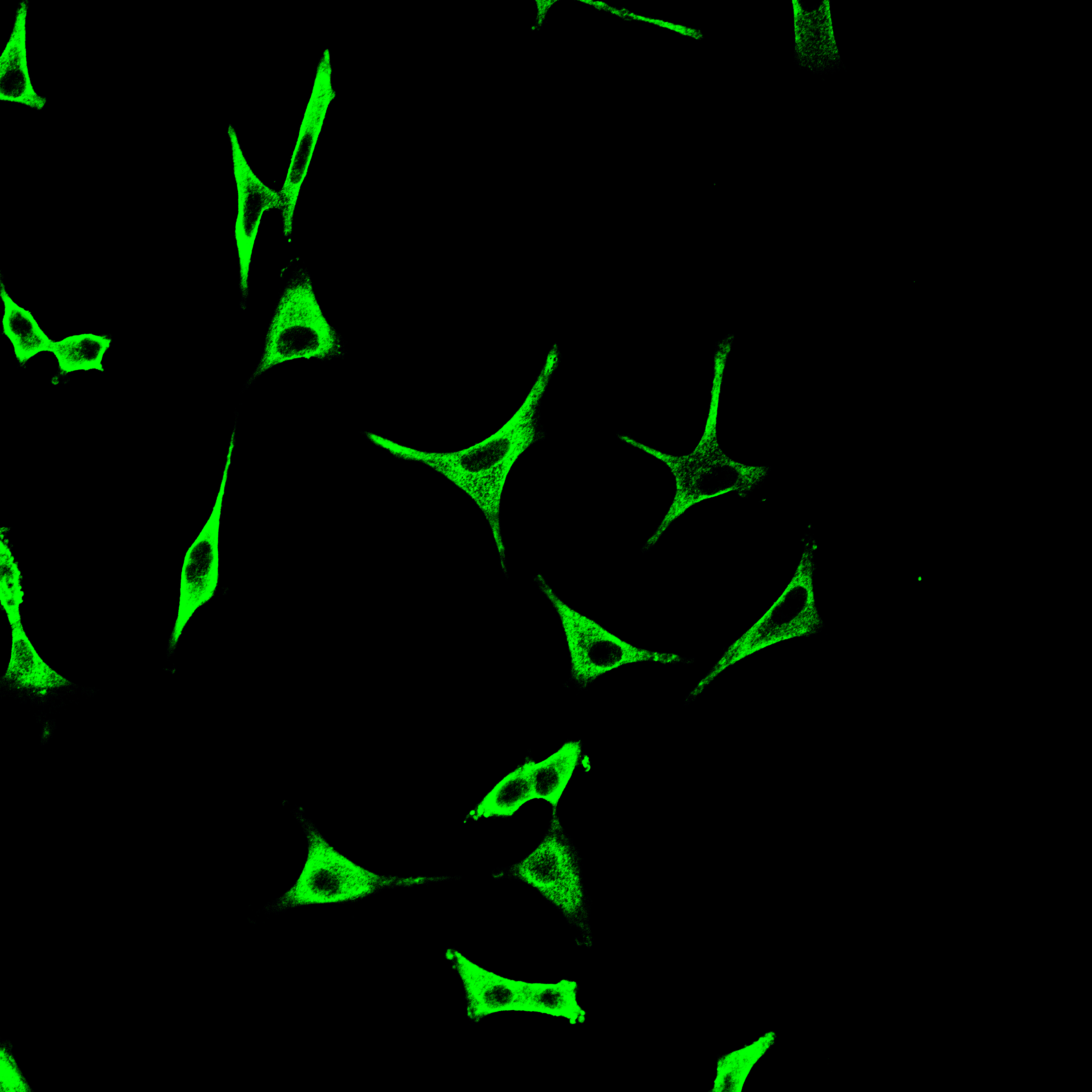

Supplement: Supplementary file 6 — Source data Fig. 4 [file 44318_2025_557_MOESM6_ESM.zip › Figure 4/4L/Figure 4L---Y993-1008F-Flag.jpg]

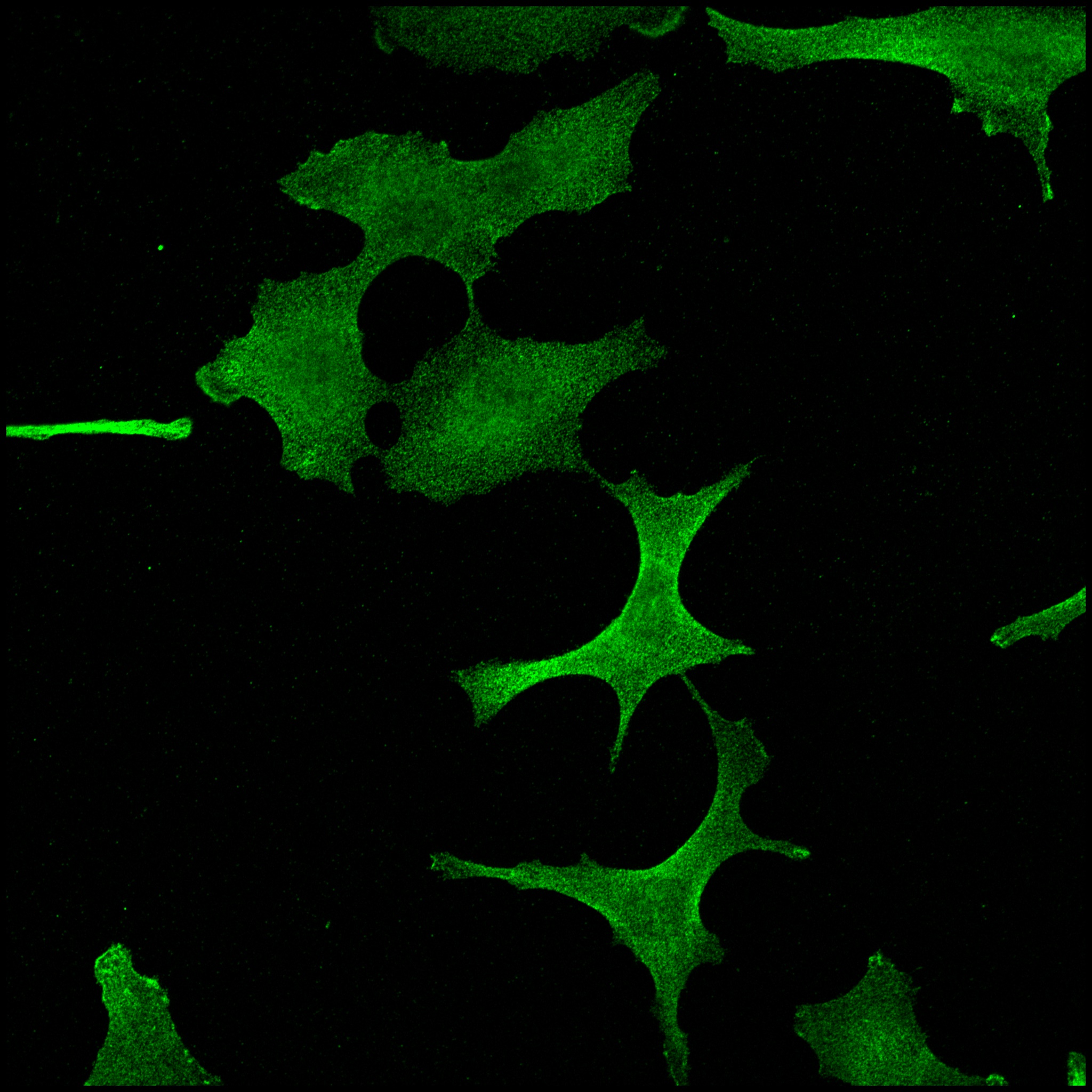

Supplement: Supplementary file 6 — Source data Fig. 4 [file 44318_2025_557_MOESM6_ESM.zip › Figure 4/4L/Figure 4L---WT-Flag.jpg]

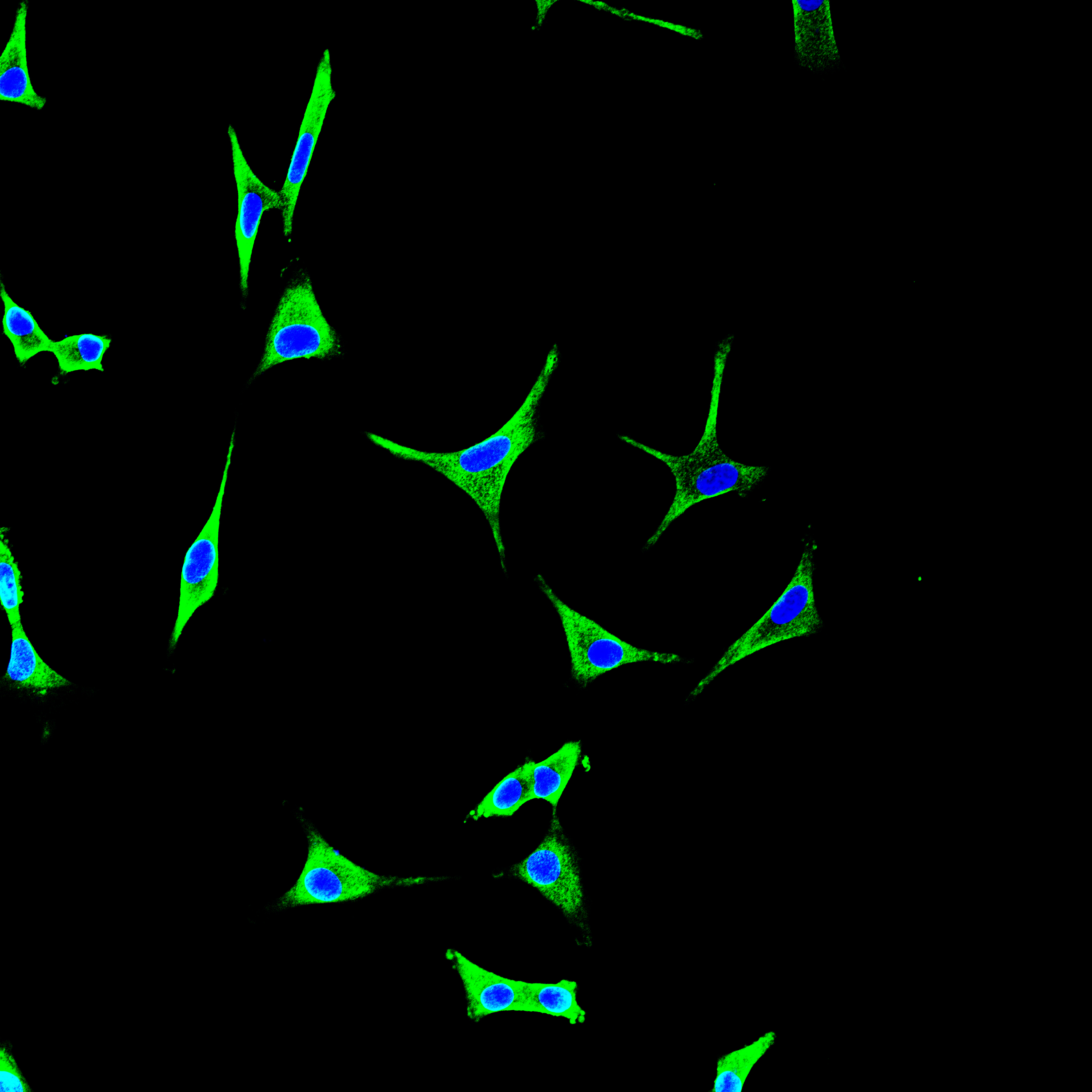

Supplement: Supplementary file 6 — Source data Fig. 4 [file 44318_2025_557_MOESM6_ESM.zip › Figure 4/4L/Figure 4L---Y993-1008F-Flag-DAPI-Merge.jpg]

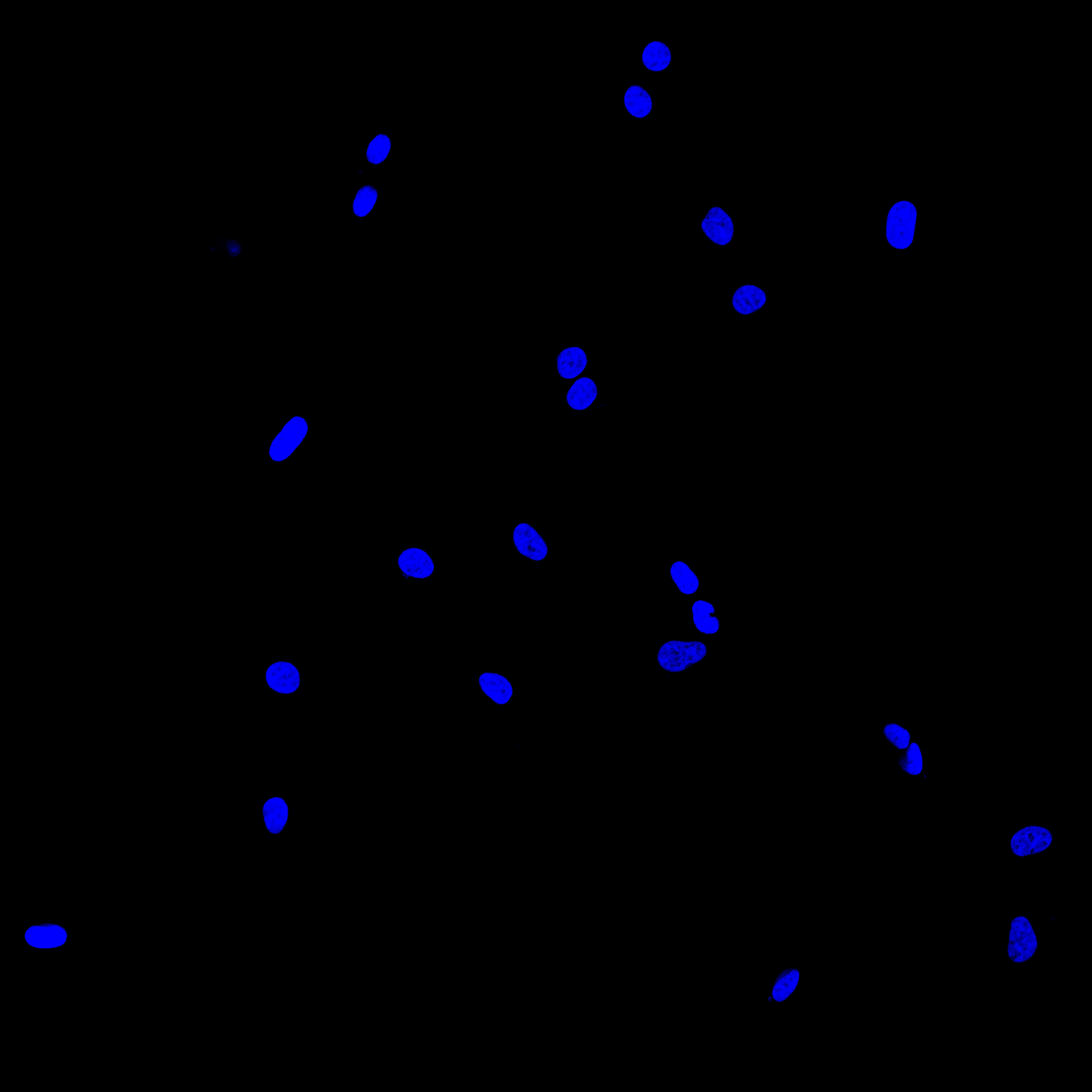

Supplement: Supplementary file 6 — Source data Fig. 4 [file 44318_2025_557_MOESM6_ESM.zip › Figure 4/4L/Figure 4L---L998A-V999A-DAPI.jpg]

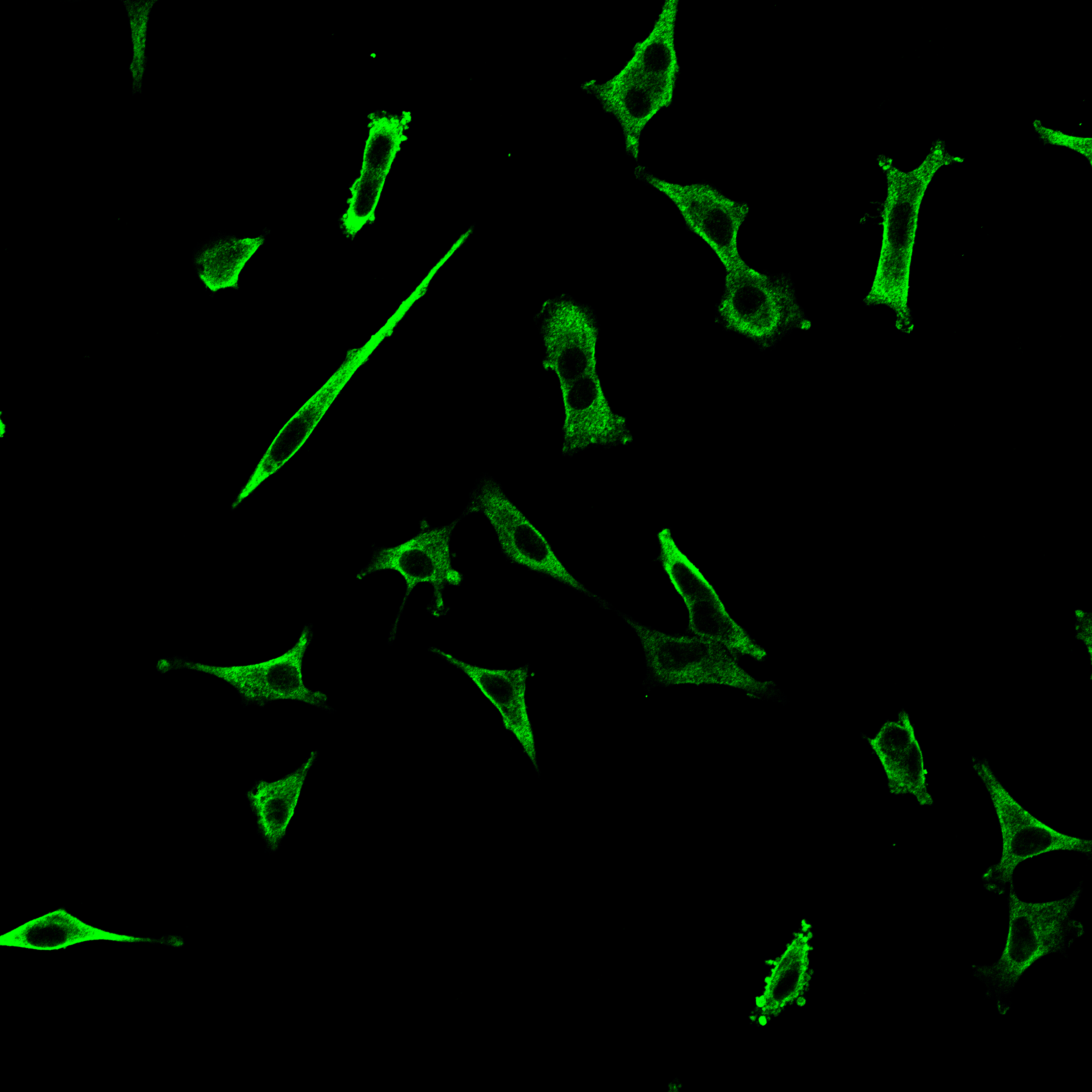

Supplement: Supplementary file 6 — Source data Fig. 4 [file 44318_2025_557_MOESM6_ESM.zip › Figure 4/4L/Figure 4L---L998A-V999A-Flag.jpg]

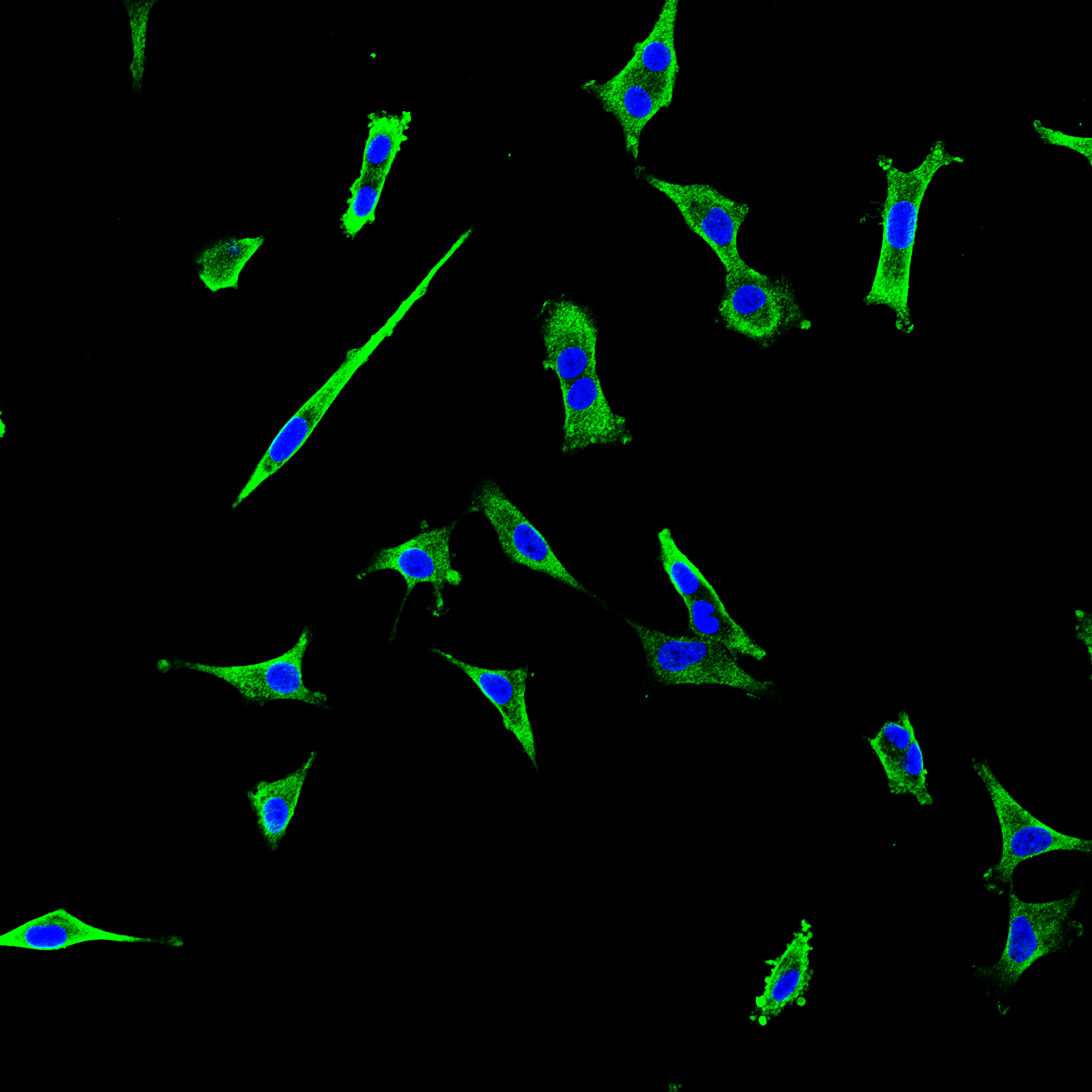

Supplement: Supplementary file 6 — Source data Fig. 4 [file 44318_2025_557_MOESM6_ESM.zip › Figure 4/4L/Figure 4L---L998A-V999A-Flag-DAPI-Merge.jpg]

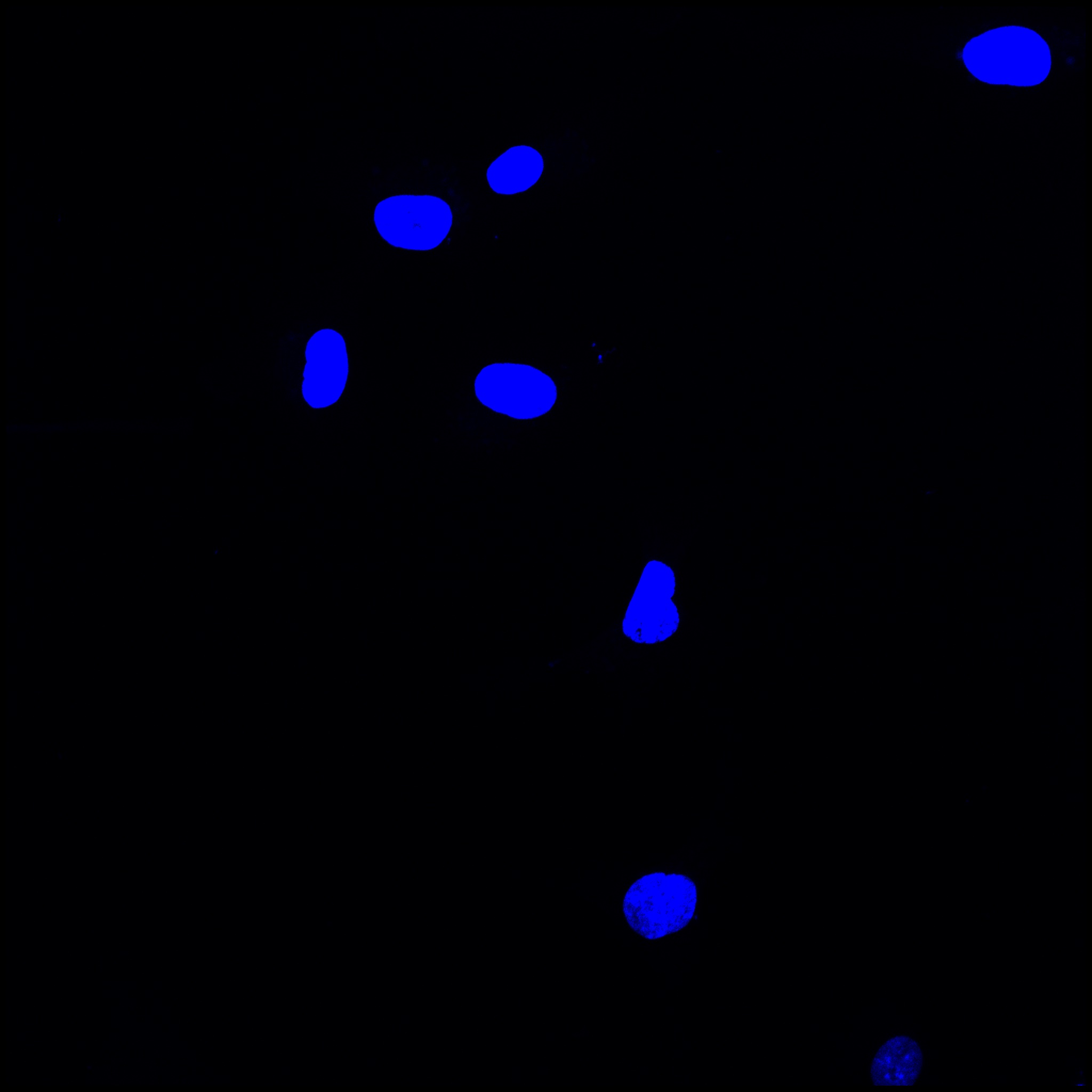

Supplement: Supplementary file 6 — Source data Fig. 4 [file 44318_2025_557_MOESM6_ESM.zip › Figure 4/4L/Figure 4L---WT-DAPI.jpg]

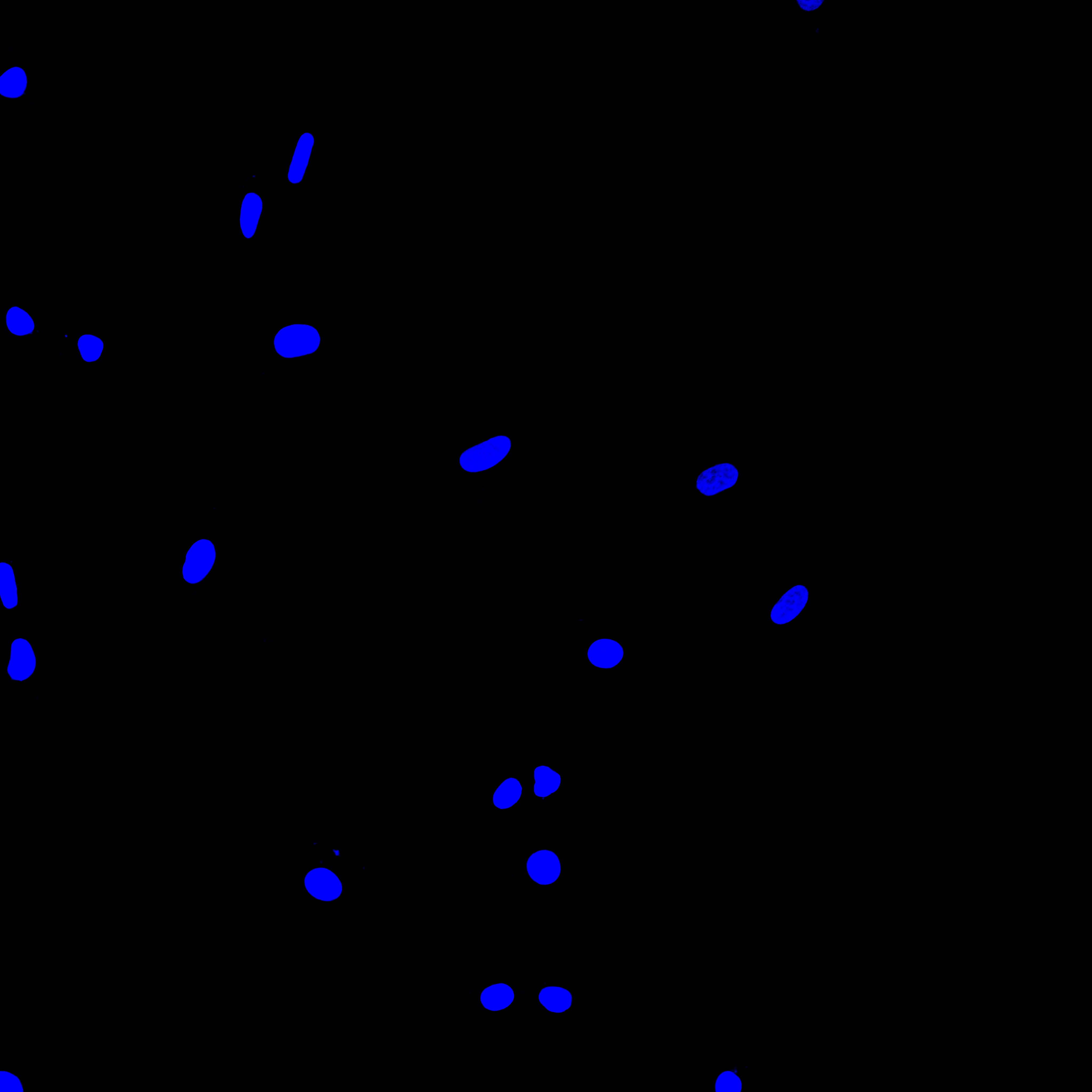

Supplement: Supplementary file 6 — Source data Fig. 4 [file 44318_2025_557_MOESM6_ESM.zip › Figure 4/4L/Figure 4L---Y993-1008F-DAPI.jpg]

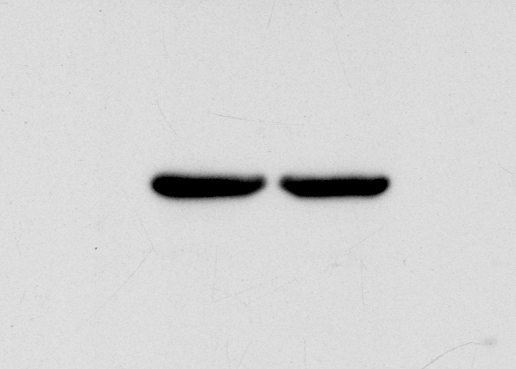

Supplement: Supplementary file 6 — Source data Fig. 4 [file 44318_2025_557_MOESM6_ESM.zip › Figure 4/4J/Figure 4J---Lysate-actin.jpg]

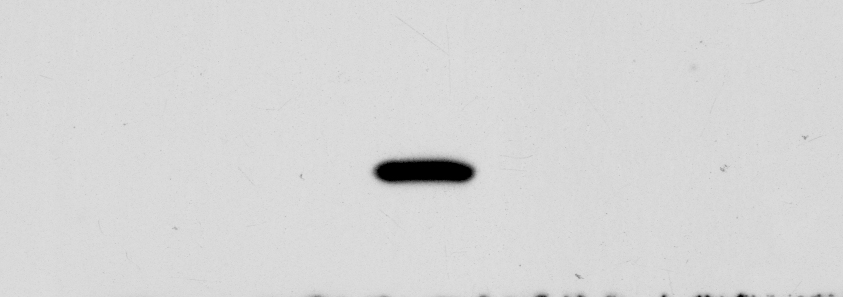

Supplement: Supplementary file 6 — Source data Fig. 4 [file 44318_2025_557_MOESM6_ESM.zip › Figure 4/4J/Figure 4J---IP-Ran.jpg]

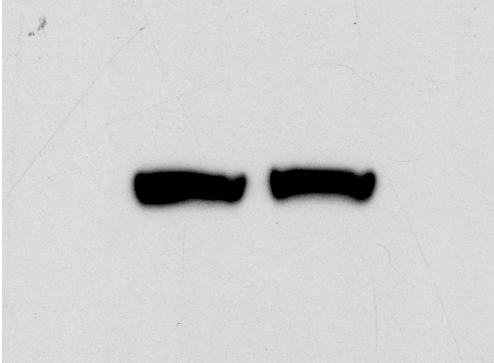

Supplement: Supplementary file 6 — Source data Fig. 4 [file 44318_2025_557_MOESM6_ESM.zip › Figure 4/4J/Figure 4J---Lysate-GLDC.jpg]

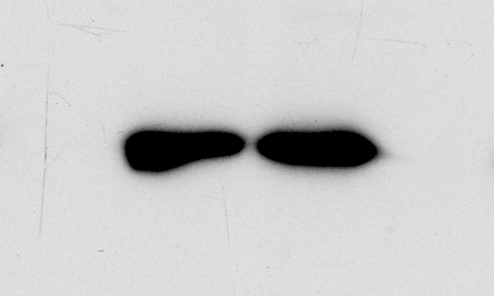

Supplement: Supplementary file 6 — Source data Fig. 4 [file 44318_2025_557_MOESM6_ESM.zip › Figure 4/4J/Figure 4J---Lysate-Ran.jpg]

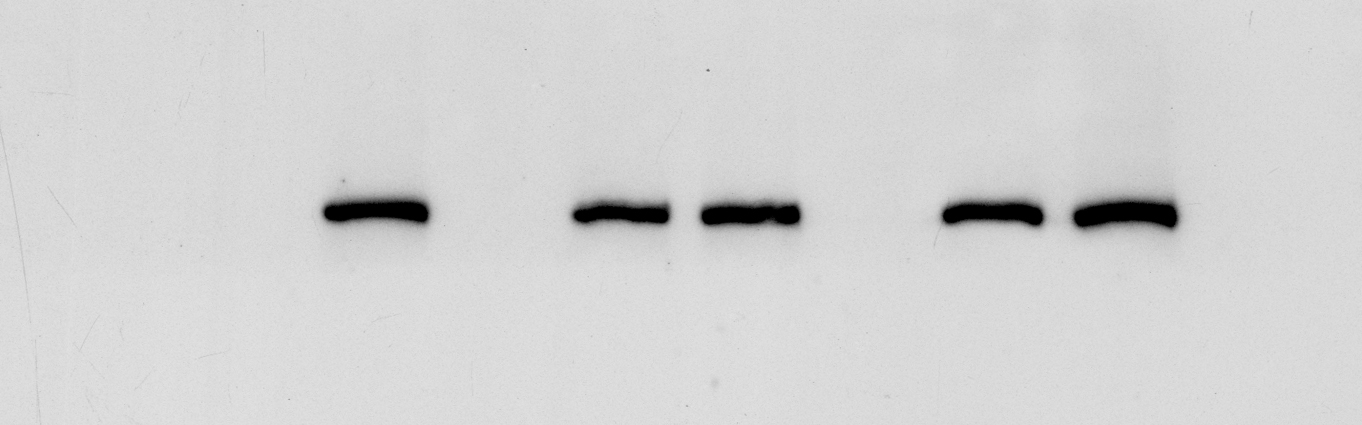

Supplement: Supplementary file 6 — Source data Fig. 4 [file 44318_2025_557_MOESM6_ESM.zip › Figure 4/4C/Figure 3C---GLDC.jpg]

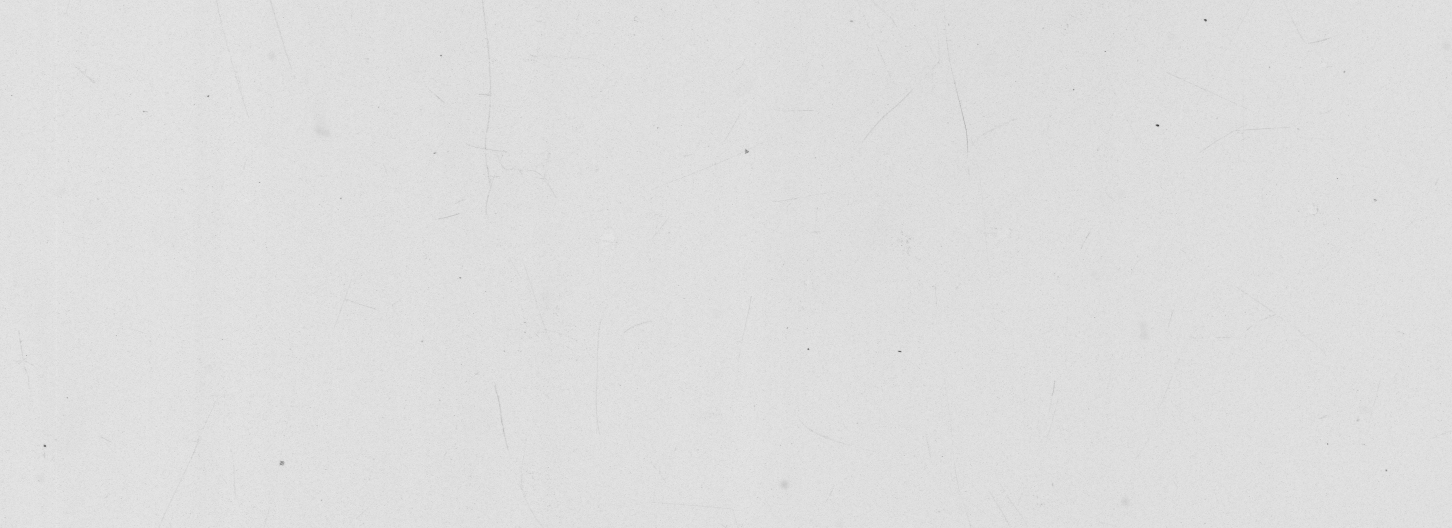

Supplement: Supplementary file 6 — Source data Fig. 4 [file 44318_2025_557_MOESM6_ESM.zip › Figure 4/4C/Figure 3C---tubulin.jpg]

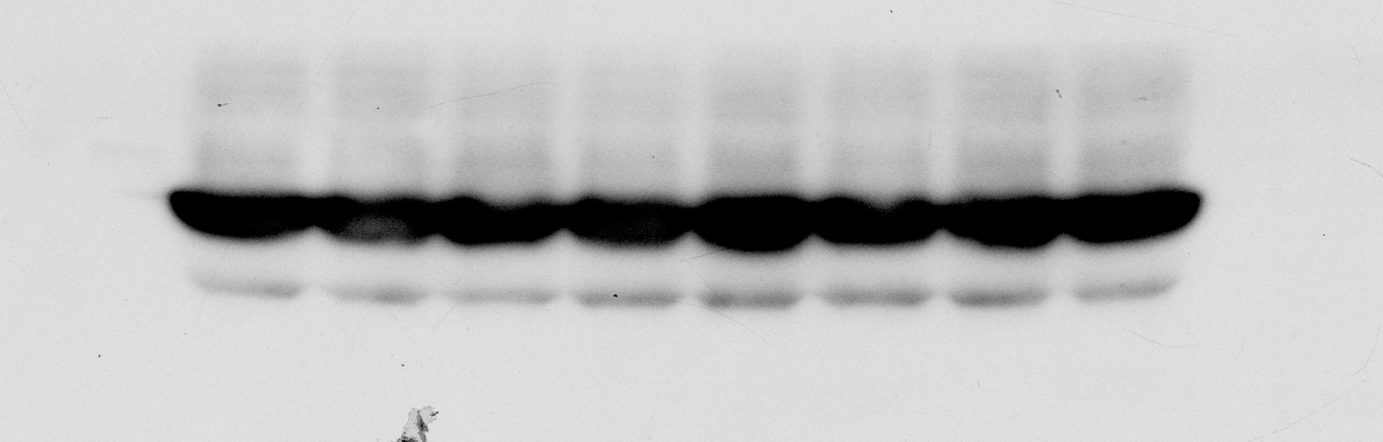

Supplement: Supplementary file 6 — Source data Fig. 4 [file 44318_2025_557_MOESM6_ESM.zip › Figure 4/4C/Figure 3C---Lamin B1.jpg]

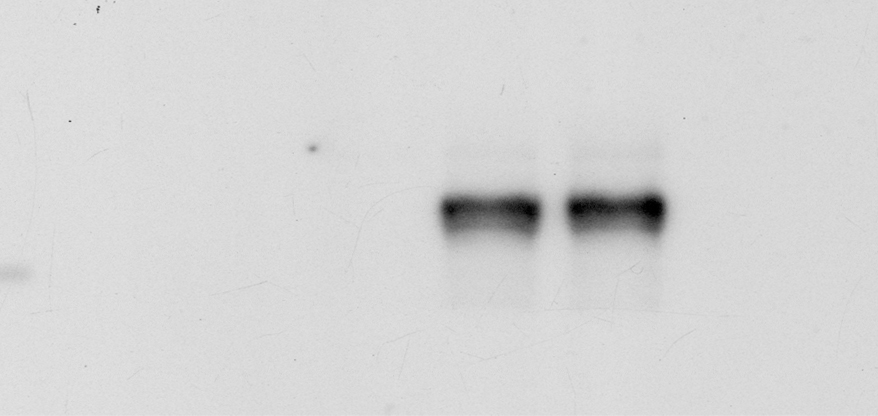

Supplement: Supplementary file 6 — Source data Fig. 4 [file 44318_2025_557_MOESM6_ESM.zip › Figure 4/4D/Figure 4D---IP-p-Tyr.jpg]

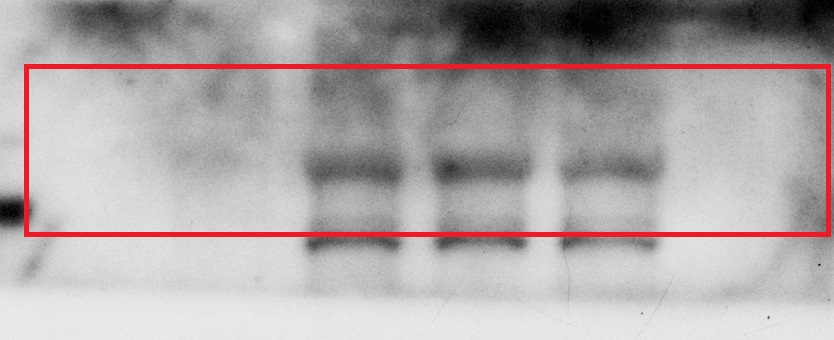

Supplement: Supplementary file 6 — Source data Fig. 4 [file 44318_2025_557_MOESM6_ESM.zip › Figure 4/4D/Figure 4D---IP-p-Ser-Thr.jpg]

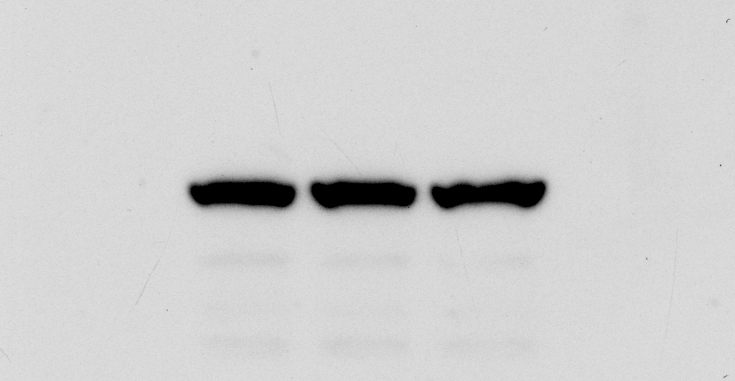

Supplement: Supplementary file 6 — Source data Fig. 4 [file 44318_2025_557_MOESM6_ESM.zip › Figure 4/4D/Figure 4D---Lysate-actin.jpg]

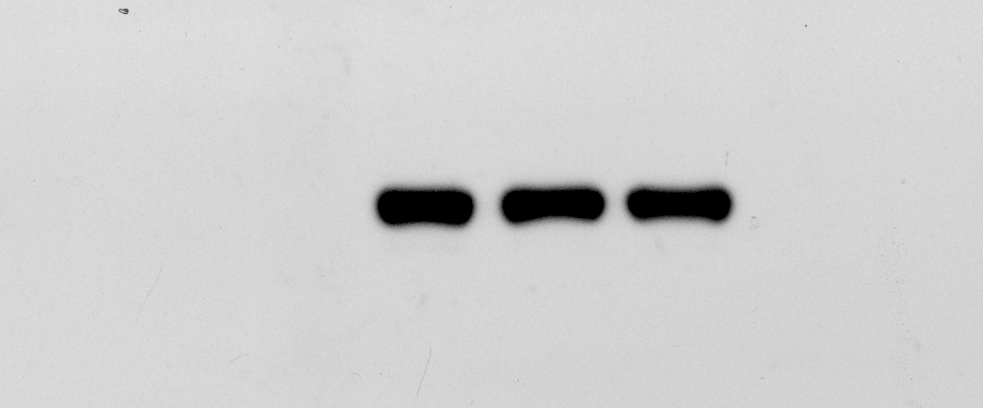

Supplement: Supplementary file 6 — Source data Fig. 4 [file 44318_2025_557_MOESM6_ESM.zip › Figure 4/4D/Figure 4D---IP-GLDC.jpg]

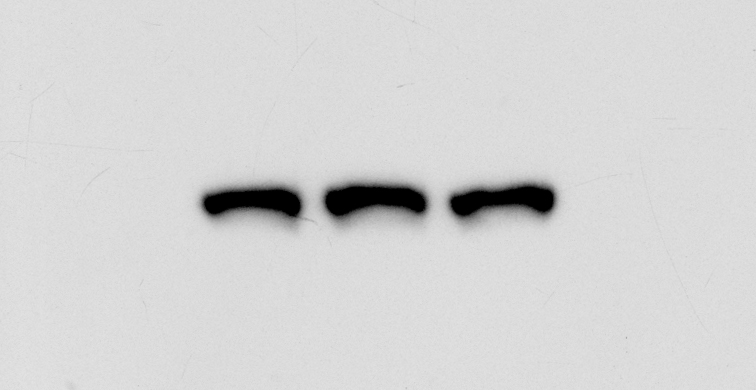

Supplement: Supplementary file 6 — Source data Fig. 4 [file 44318_2025_557_MOESM6_ESM.zip › Figure 4/4D/Figure 4D---Lysate-GLDC.jpg]

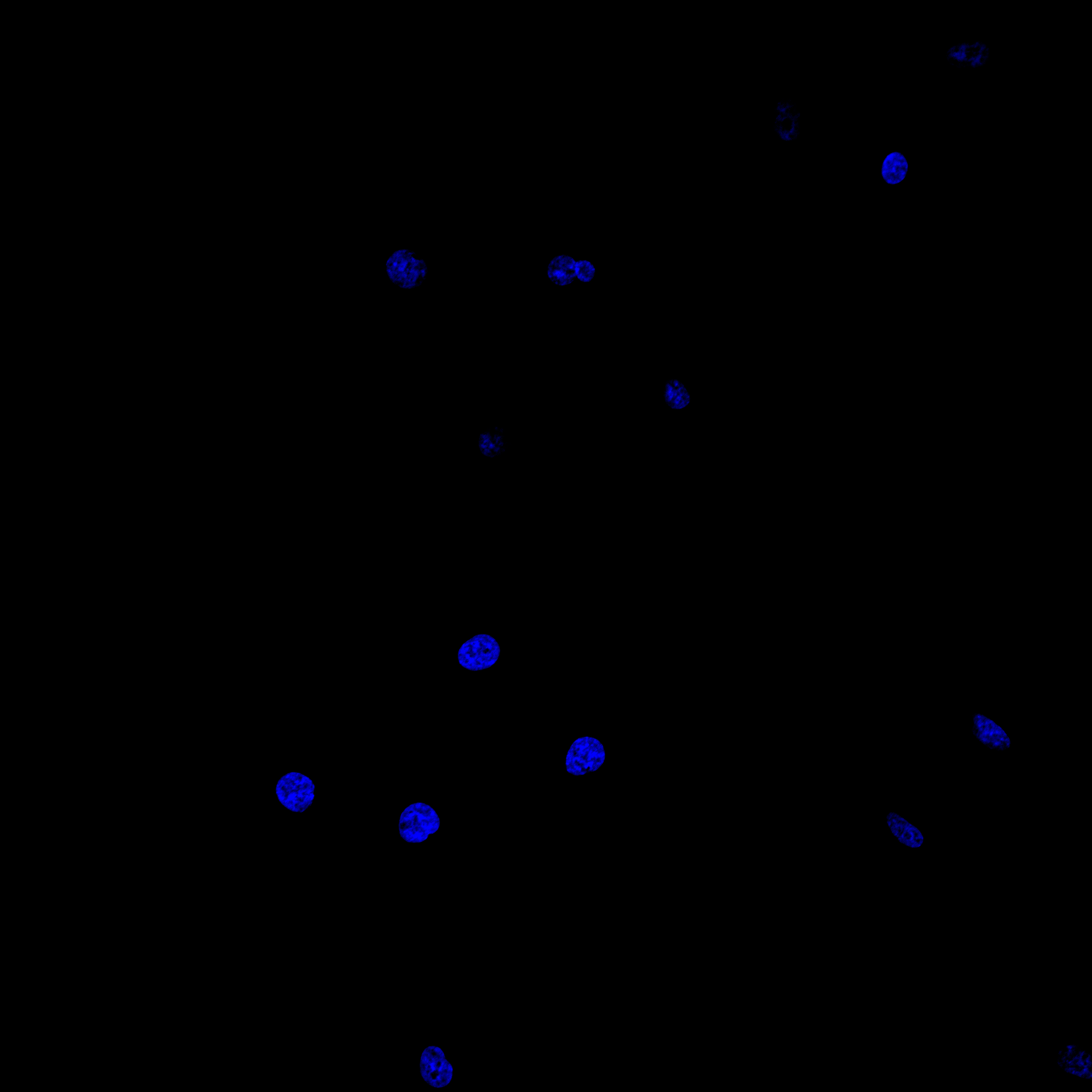

Supplement: Supplementary file 6 — Source data Fig. 4 [file 44318_2025_557_MOESM6_ESM.zip › Figure 4/4A/Figure 4A---EGF--DAPI.jpg]

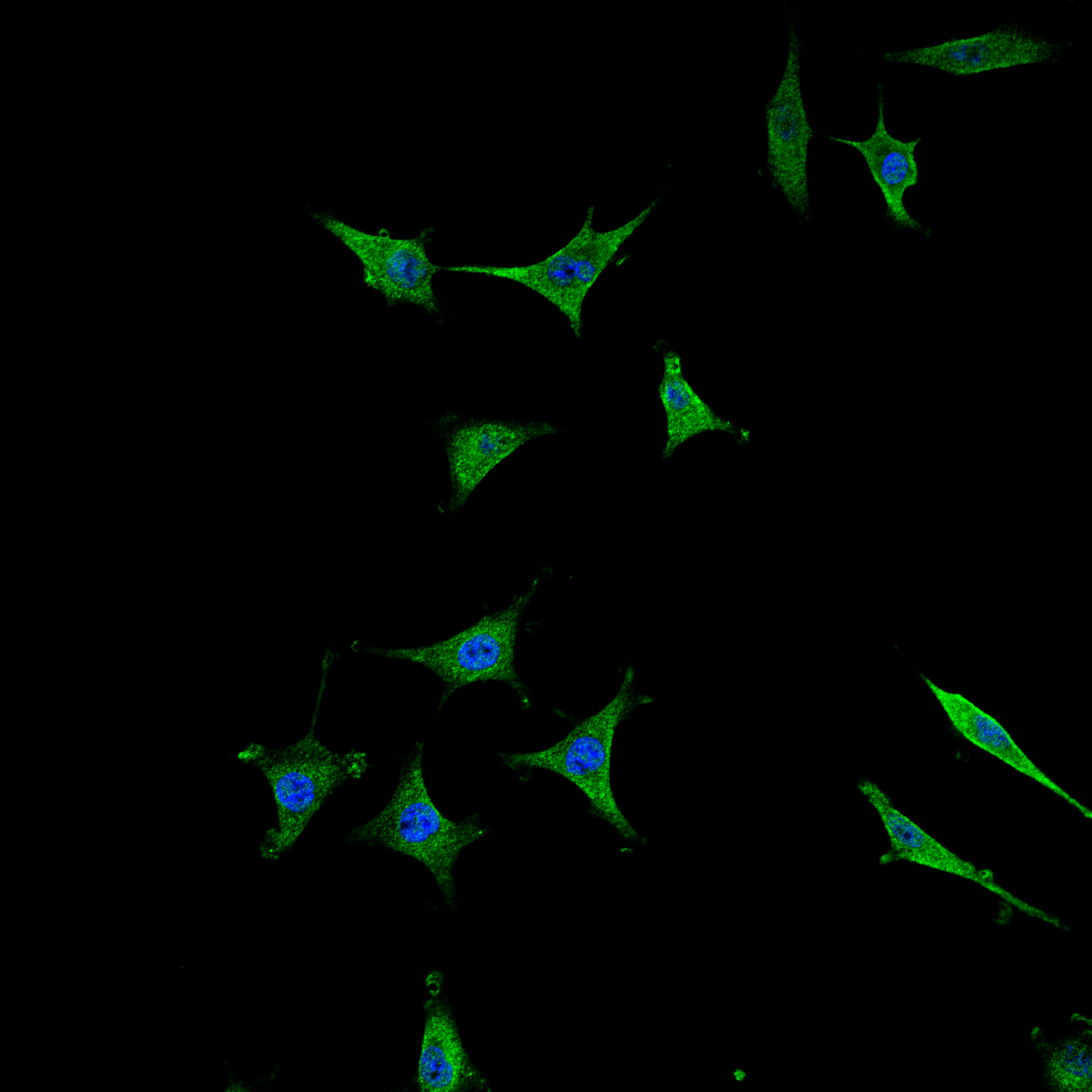

Supplement: Supplementary file 6 — Source data Fig. 4 [file 44318_2025_557_MOESM6_ESM.zip › Figure 4/4A/Figure 4A---EGF--GLDC-DAPI-Merge.jpg]

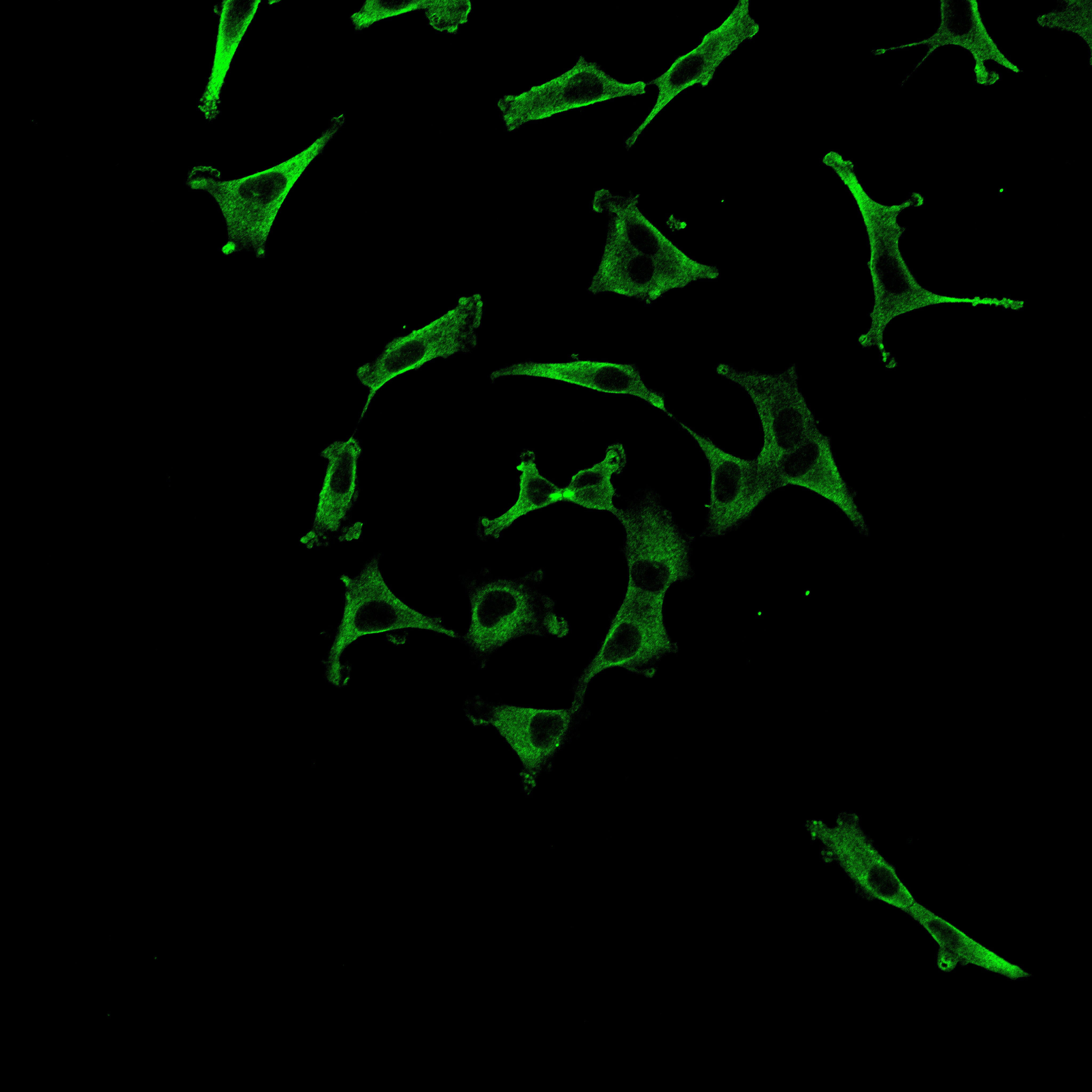

Supplement: Supplementary file 6 — Source data Fig. 4 [file 44318_2025_557_MOESM6_ESM.zip › Figure 4/4A/Figure 4A---Mock--GLDC.jpg]

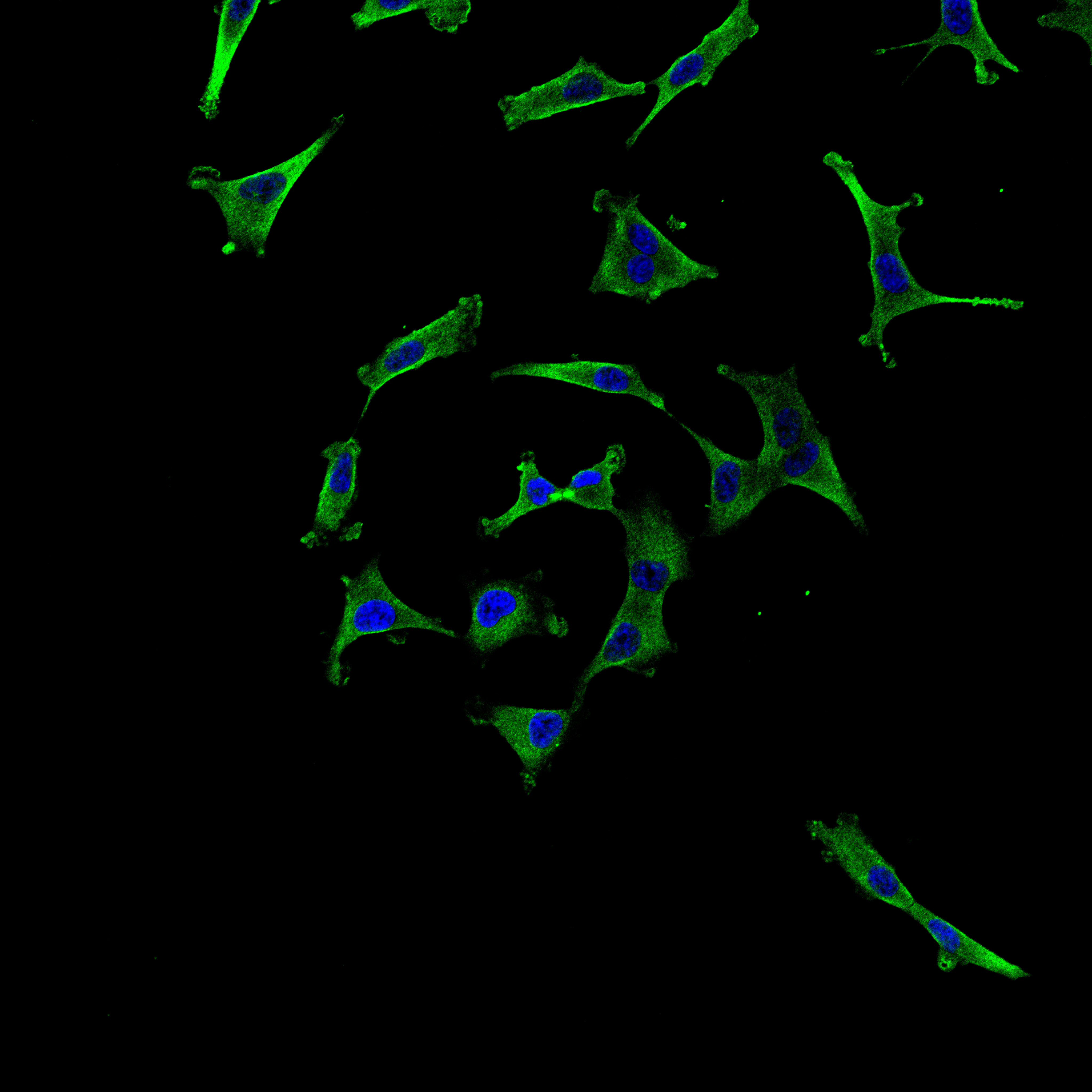

Supplement: Supplementary file 6 — Source data Fig. 4 [file 44318_2025_557_MOESM6_ESM.zip › Figure 4/4A/Figure 4A---Mock--GLDC-DAPI-Merge.jpg]

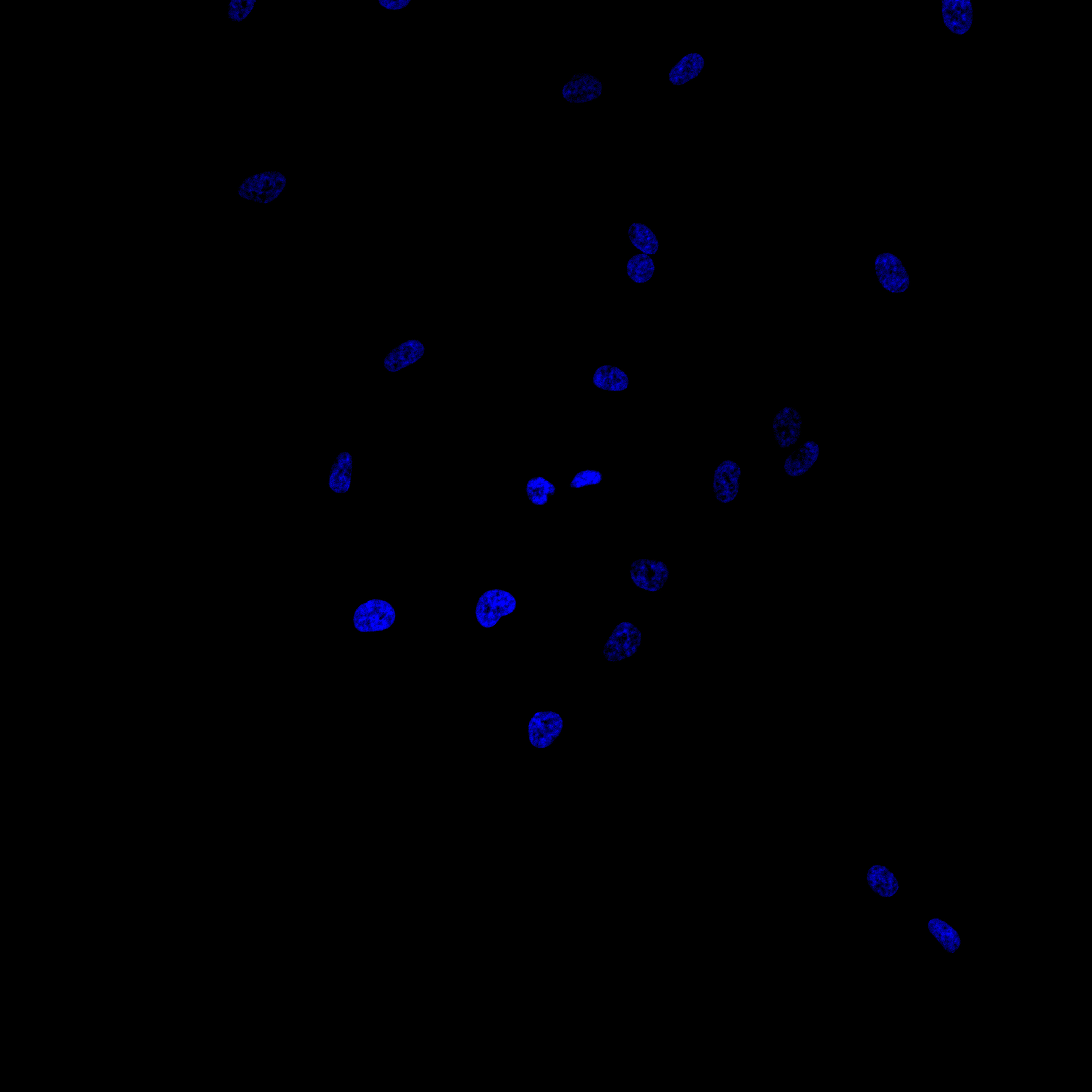

Supplement: Supplementary file 6 — Source data Fig. 4 [file 44318_2025_557_MOESM6_ESM.zip › Figure 4/4A/Figure 4A---Mock--DAPI.jpg]

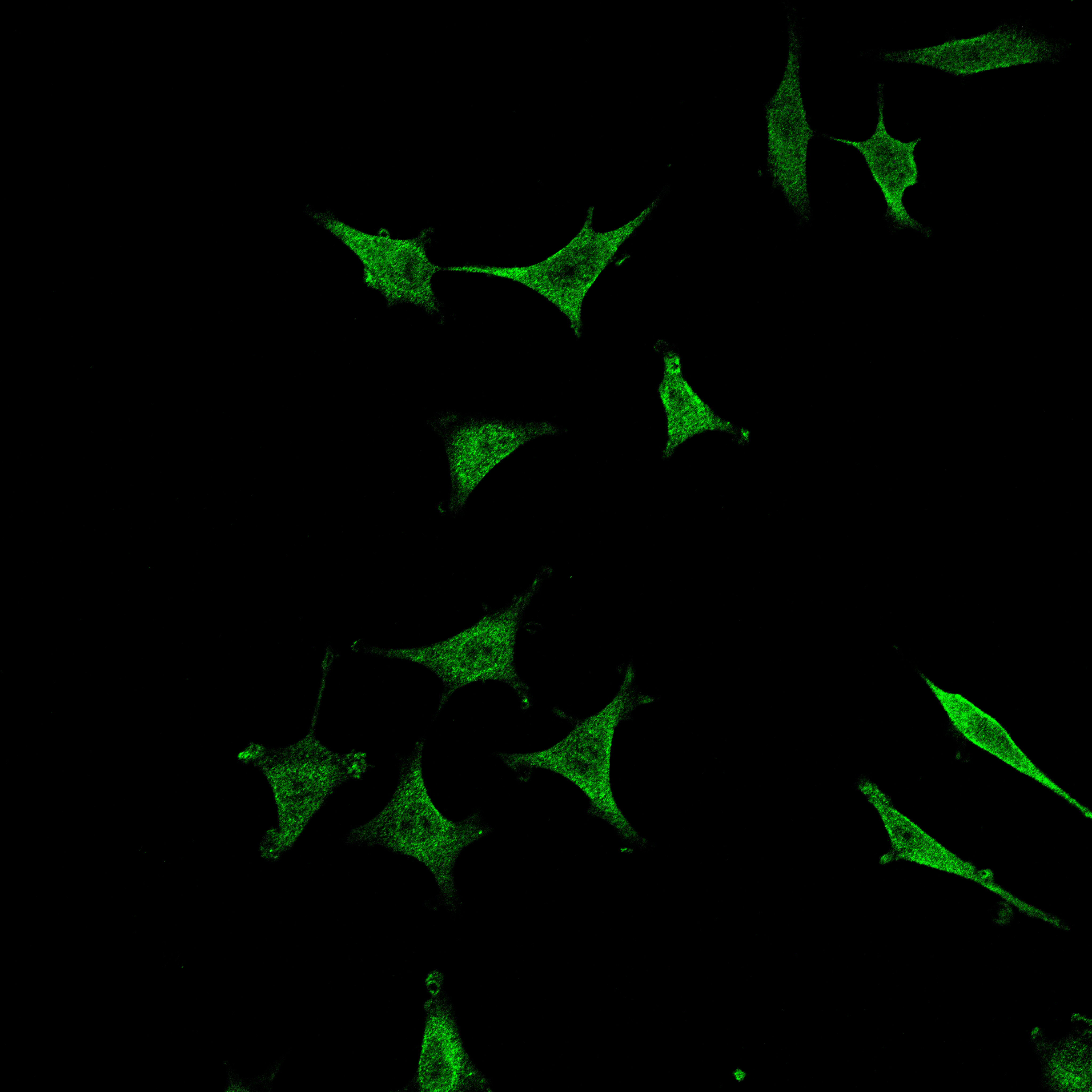

Supplement: Supplementary file 6 — Source data Fig. 4 [file 44318_2025_557_MOESM6_ESM.zip › Figure 4/4A/Figure 4A---EGF--GLDC.jpg]

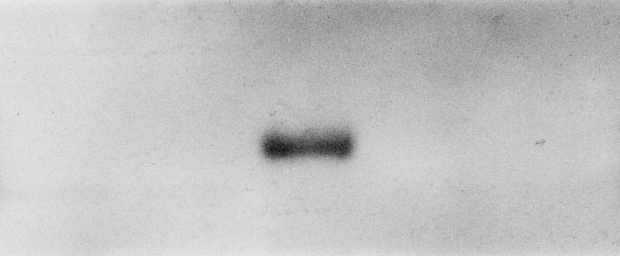

Supplement: Supplementary file 6 — Source data Fig. 4 [file 44318_2025_557_MOESM6_ESM.zip › Figure 4/4F/Figure 4F---pGLDCY1008.jpg]

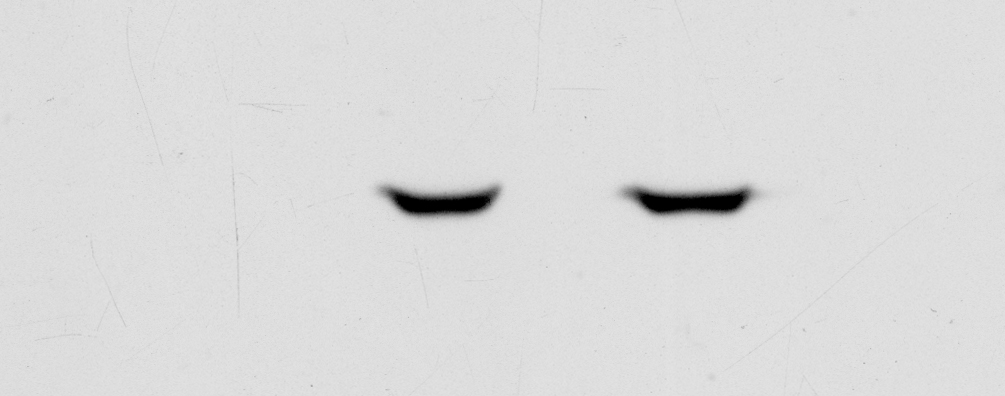

Supplement: Supplementary file 6 — Source data Fig. 4 [file 44318_2025_557_MOESM6_ESM.zip › Figure 4/4F/Figure 4F---SRC.jpg]

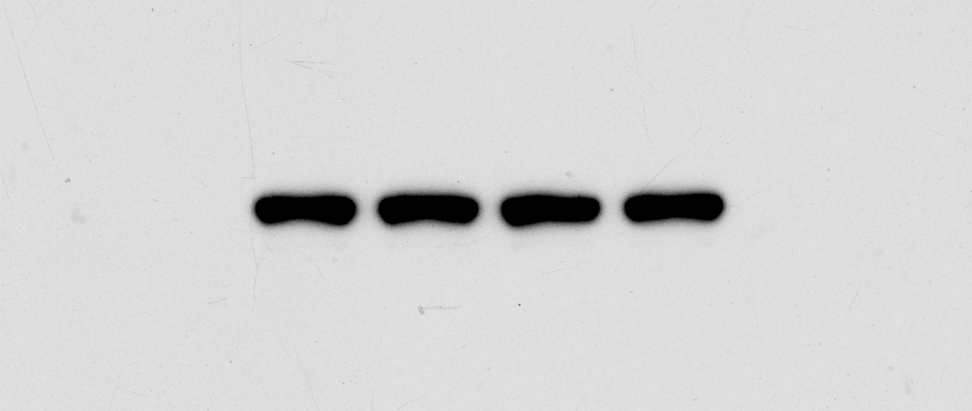

Supplement: Supplementary file 6 — Source data Fig. 4 [file 44318_2025_557_MOESM6_ESM.zip › Figure 4/4F/Figure 4F---Flag.jpg]

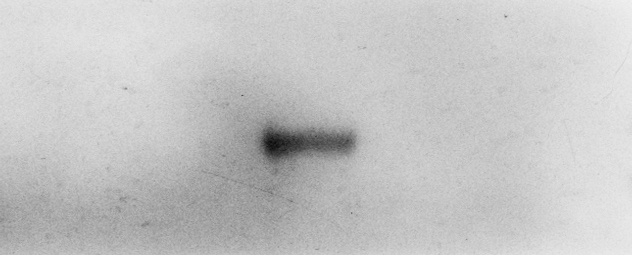

Supplement: Supplementary file 6 — Source data Fig. 4 [file 44318_2025_557_MOESM6_ESM.zip › Figure 4/4F/Figure 4F---pGLDCY993.jpg]

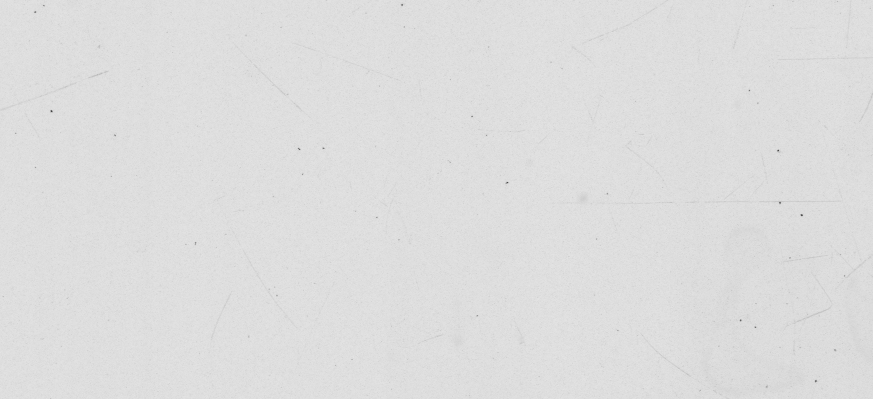

Supplement: Supplementary file 6 — Source data Fig. 4 [file 44318_2025_557_MOESM6_ESM.zip › Figure 4/4H/Figure 4H---tubulin.jpg]

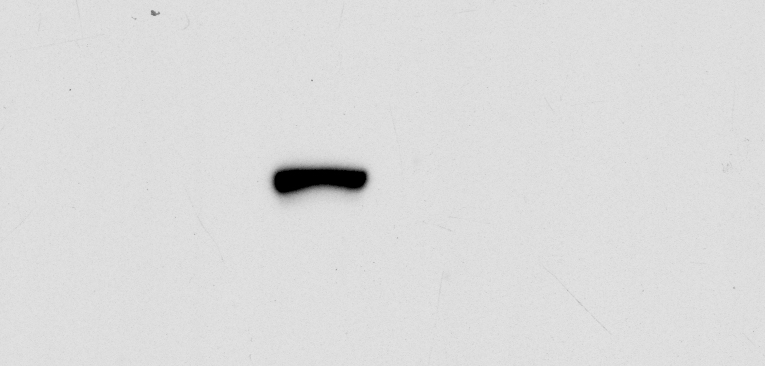

Supplement: Supplementary file 6 — Source data Fig. 4 [file 44318_2025_557_MOESM6_ESM.zip › Figure 4/4H/Figure 4H---GLDC.jpg]

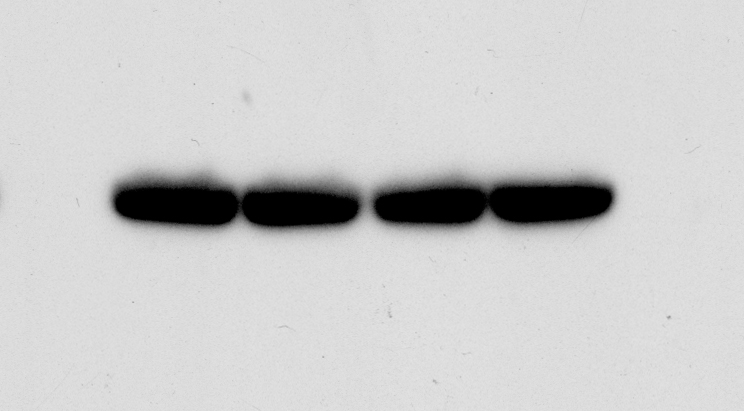

Supplement: Supplementary file 6 — Source data Fig. 4 [file 44318_2025_557_MOESM6_ESM.zip › Figure 4/4H/Figure 4H---Lamin B1.jpg]

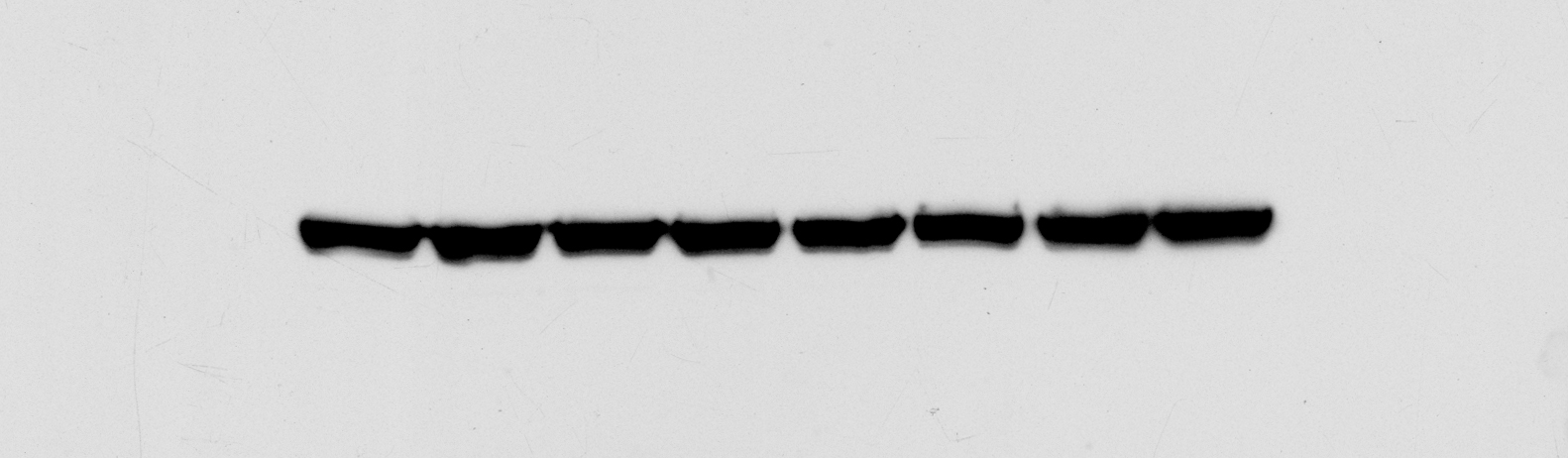

Supplement: Supplementary file 6 — Source data Fig. 4 [file 44318_2025_557_MOESM6_ESM.zip › Figure 4/4I/Figure 4I---Lysate-Flag.jpg]

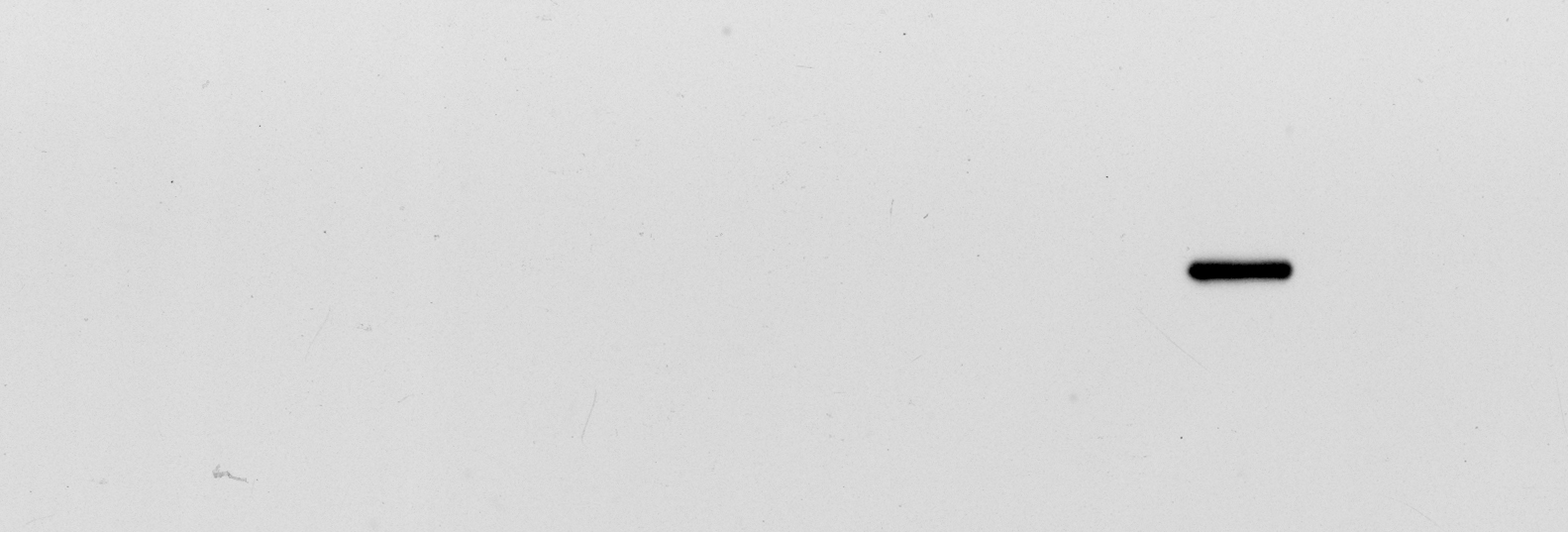

Supplement: Supplementary file 6 — Source data Fig. 4 [file 44318_2025_557_MOESM6_ESM.zip › Figure 4/4I/Figure 4I---IP-Flag.jpg]

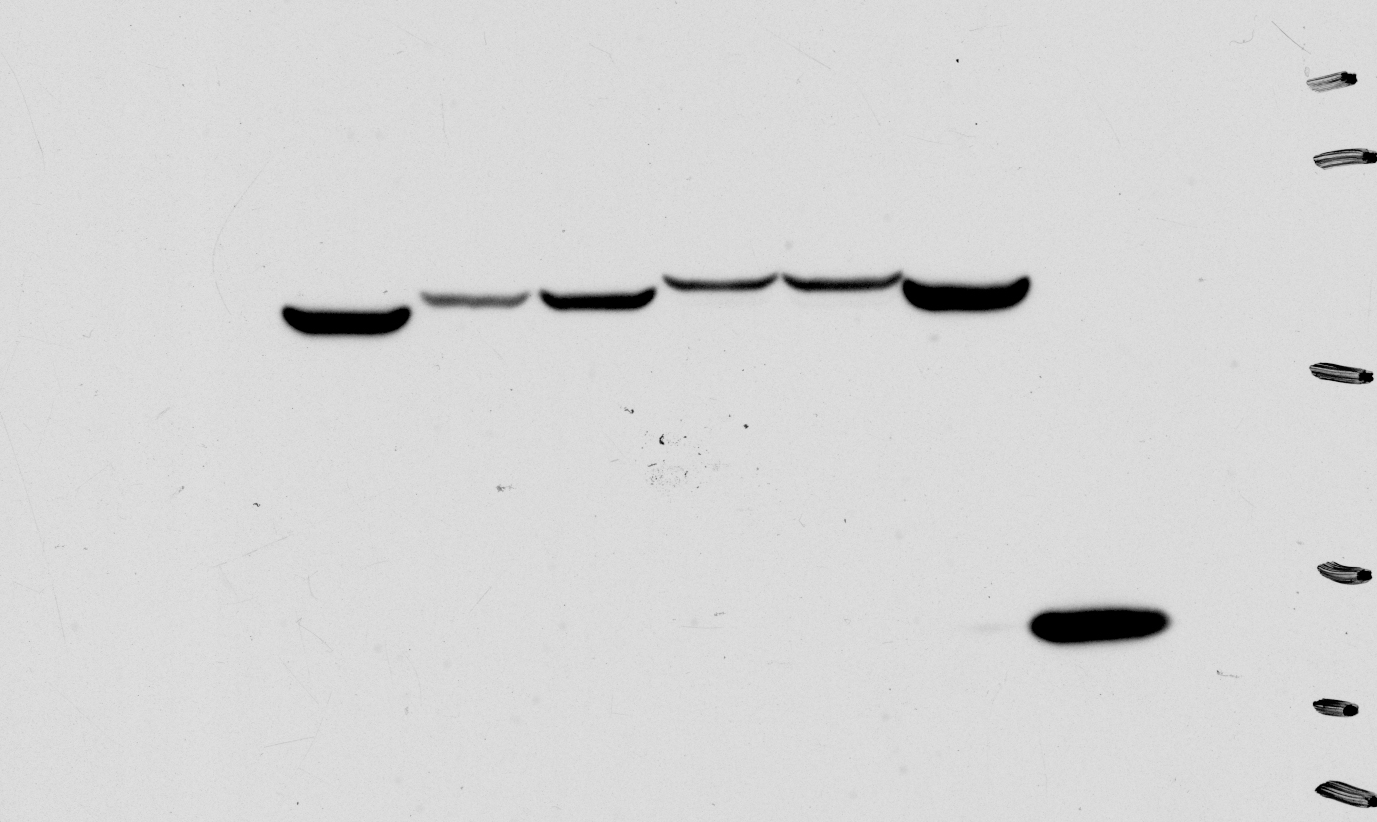

Supplement: Supplementary file 6 — Source data Fig. 4 [file 44318_2025_557_MOESM6_ESM.zip › Figure 4/4I/Figure 4I---Lysate-HA.jpg]

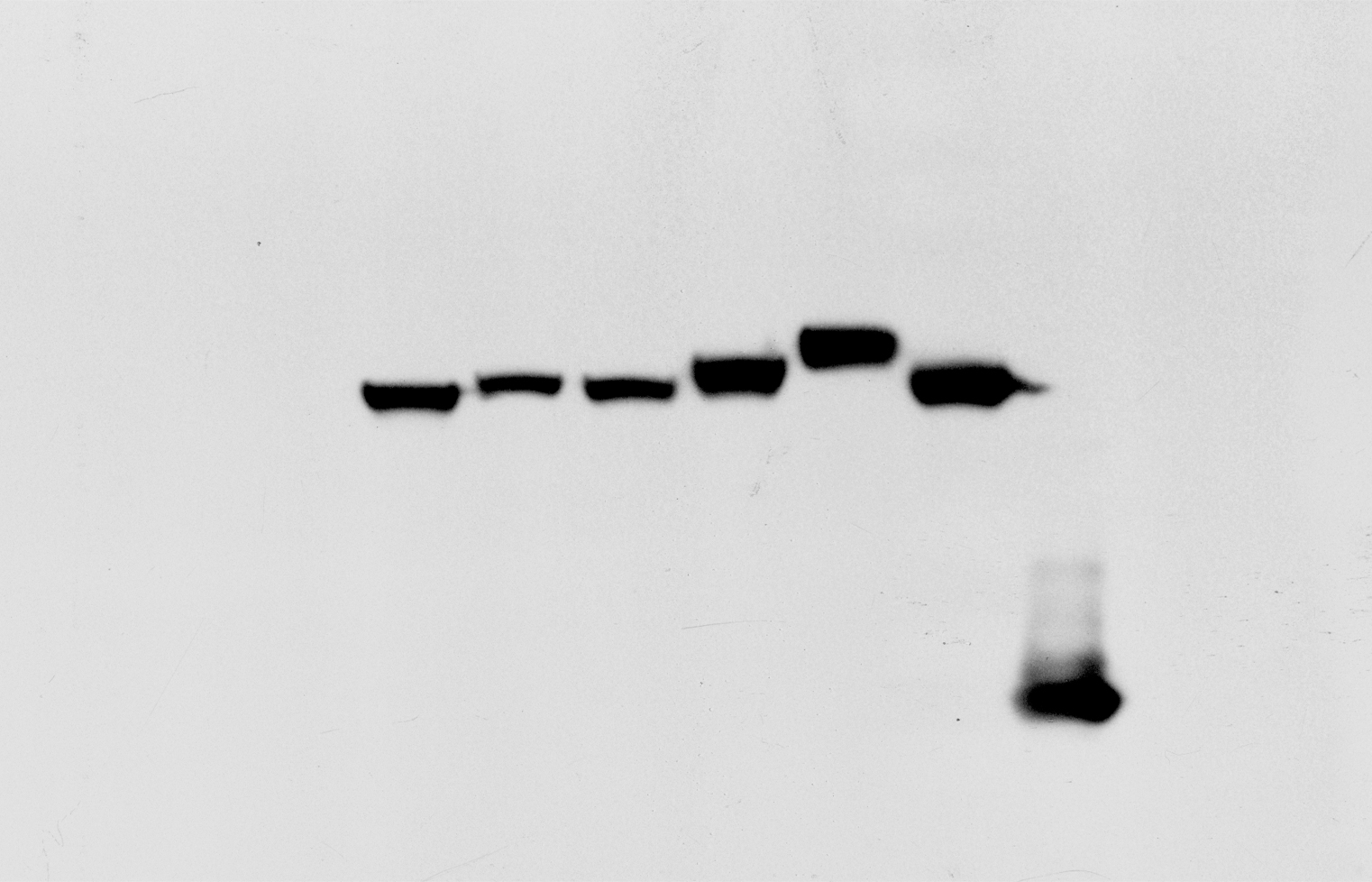

Supplement: Supplementary file 6 — Source data Fig. 4 [file 44318_2025_557_MOESM6_ESM.zip › Figure 4/4I/Figure 4I---IP-HA.jpg]

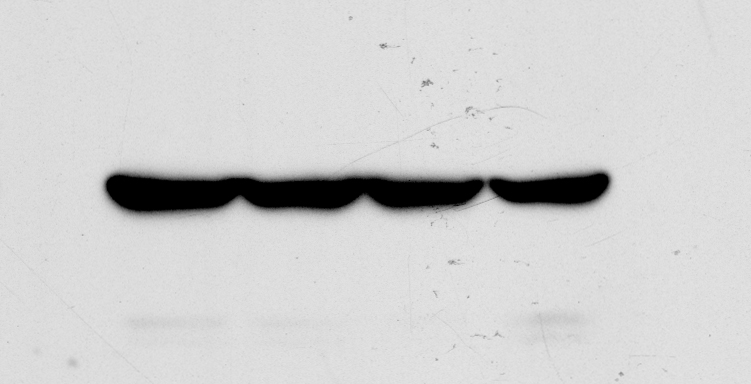

Supplement: Supplementary file 6 — Source data Fig. 4 [file 44318_2025_557_MOESM6_ESM.zip › Figure 4/4G/Figure 4G---Lysate-actin.jpg]

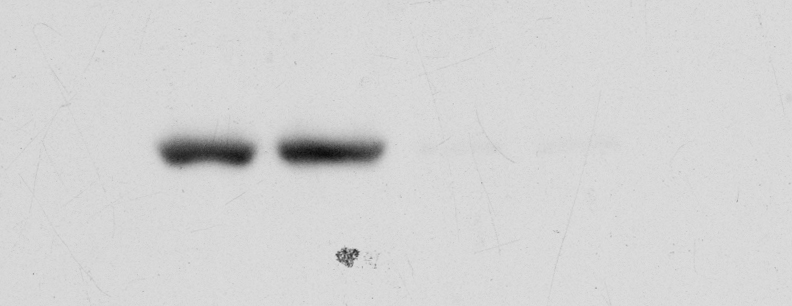

Supplement: Supplementary file 6 — Source data Fig. 4 [file 44318_2025_557_MOESM6_ESM.zip › Figure 4/4G/Figure 4G---Lysate-SRC.jpg]

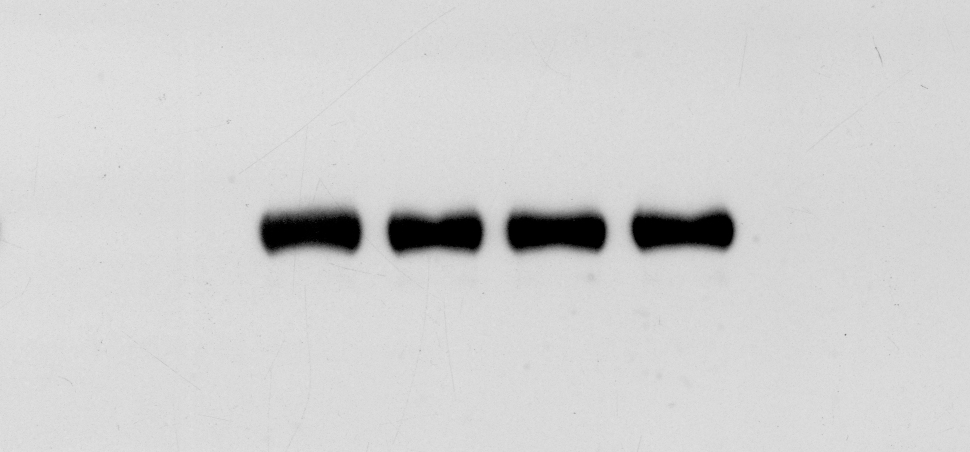

Supplement: Supplementary file 6 — Source data Fig. 4 [file 44318_2025_557_MOESM6_ESM.zip › Figure 4/4G/Figure 4G---IP-GLDC.jpg]

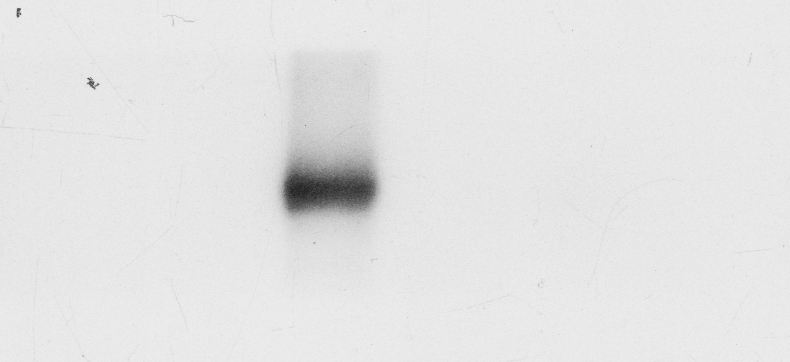

Supplement: Supplementary file 6 — Source data Fig. 4 [file 44318_2025_557_MOESM6_ESM.zip › Figure 4/4G/Figure 4G---IP-pGLDCY993.jpg]

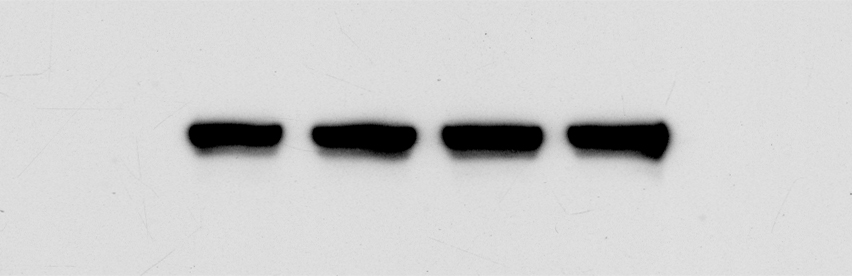

Supplement: Supplementary file 6 — Source data Fig. 4 [file 44318_2025_557_MOESM6_ESM.zip › Figure 4/4G/Figure 4G---Lysate-GLDC.jpg]

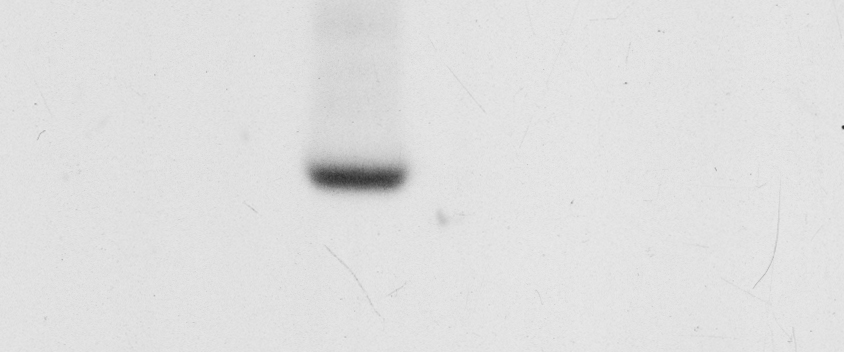

Supplement: Supplementary file 6 — Source data Fig. 4 [file 44318_2025_557_MOESM6_ESM.zip › Figure 4/4G/Figure 4G---Lysate-pSRCY419.jpg]

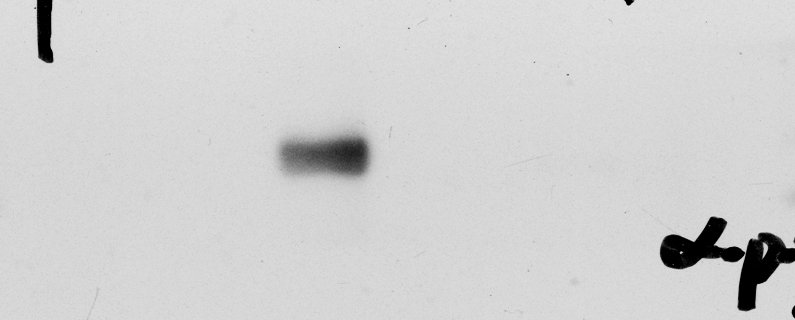

Supplement: Supplementary file 6 — Source data Fig. 4 [file 44318_2025_557_MOESM6_ESM.zip › Figure 4/4G/Figure 4G---IP-pGLDCY1008.jpg]

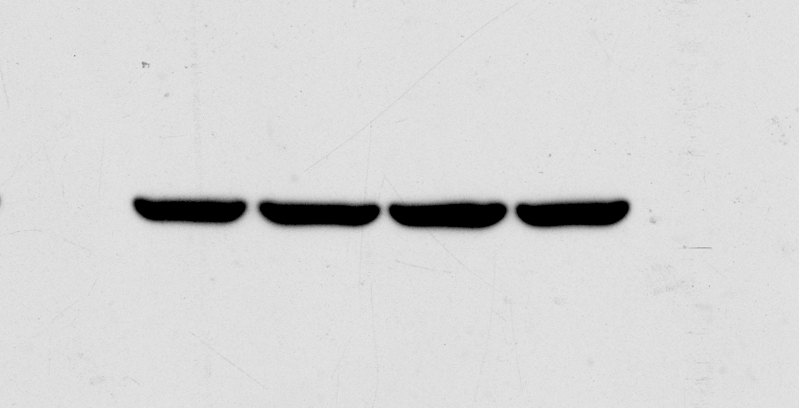

Supplement: Supplementary file 7 — Source data Fig. 5 [file 44318_2025_557_MOESM7_ESM.zip › Figure 5/5I/Figure 5I---Lysate-Sactin.jpg]

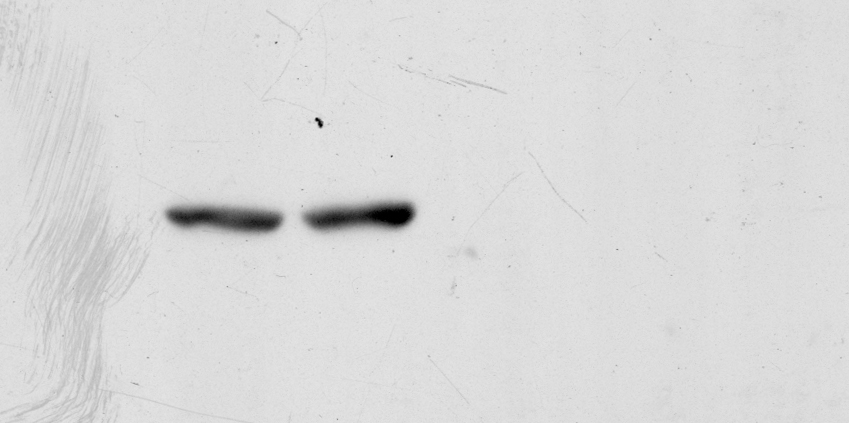

Supplement: Supplementary file 7 — Source data Fig. 5 [file 44318_2025_557_MOESM7_ESM.zip › Figure 5/5I/Figure 5I---Lysate-SMARCE1.jpg]

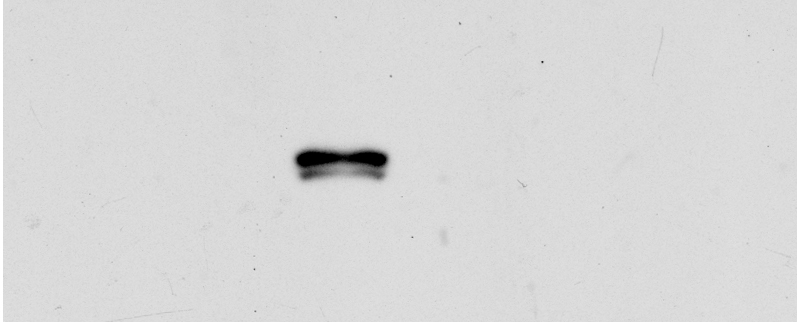

Supplement: Supplementary file 7 — Source data Fig. 5 [file 44318_2025_557_MOESM7_ESM.zip › Figure 5/5I/Figure 5I---IP-STAT1.jpg]

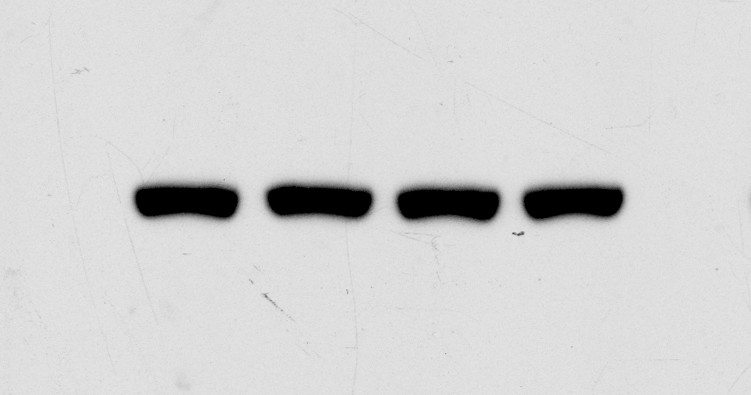

Supplement: Supplementary file 7 — Source data Fig. 5 [file 44318_2025_557_MOESM7_ESM.zip › Figure 5/5I/Figure 5I---Lysate-STAT1.jpg]

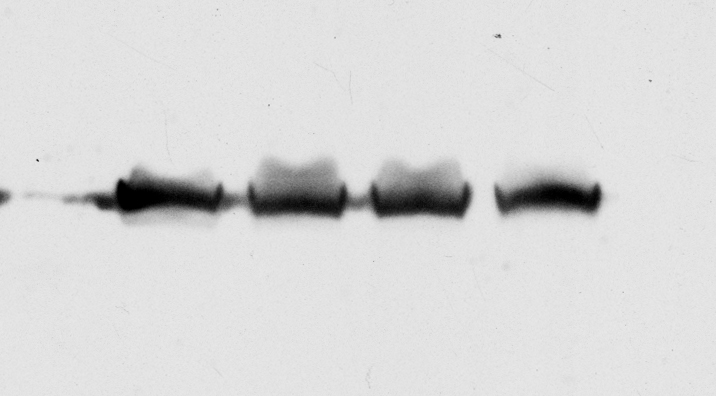

Supplement: Supplementary file 7 — Source data Fig. 5 [file 44318_2025_557_MOESM7_ESM.zip › Figure 5/5I/Figure 5I---Lysate-GLDC.jpg]

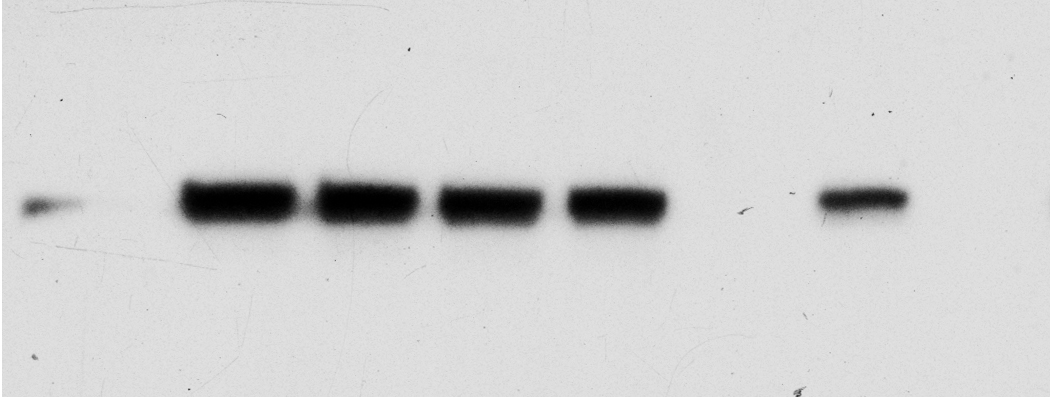

Supplement: Supplementary file 7 — Source data Fig. 5 [file 44318_2025_557_MOESM7_ESM.zip › Figure 5/5A/Figure 5A---Lysate-GLDC.jpg]

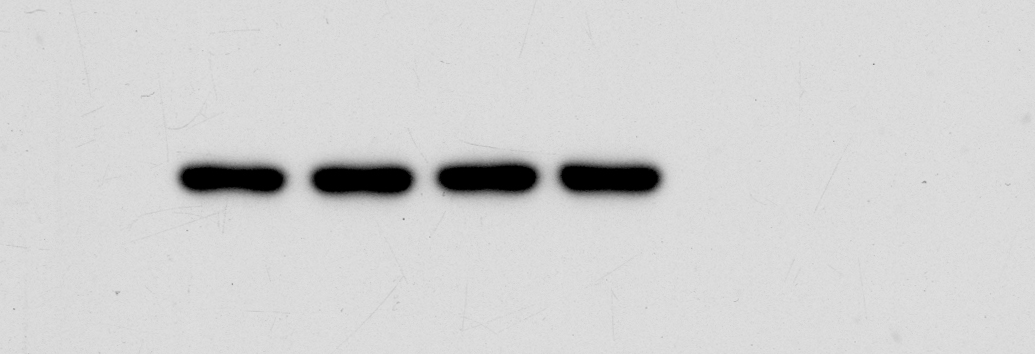

Supplement: Supplementary file 7 — Source data Fig. 5 [file 44318_2025_557_MOESM7_ESM.zip › Figure 5/5A/Figure 5A---Lysate-tubulin.jpg]

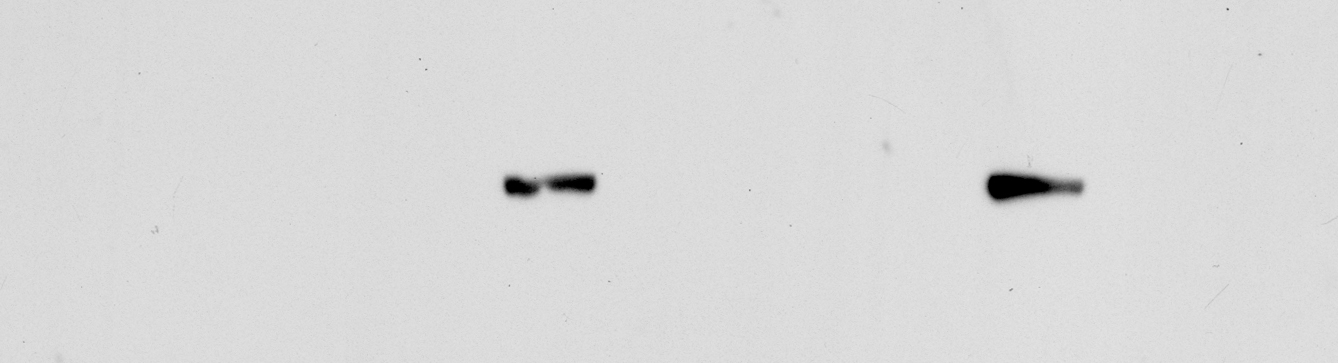

Supplement: Supplementary file 7 — Source data Fig. 5 [file 44318_2025_557_MOESM7_ESM.zip › Figure 5/5A/Figure 5A---IP-GLDC.jpg]

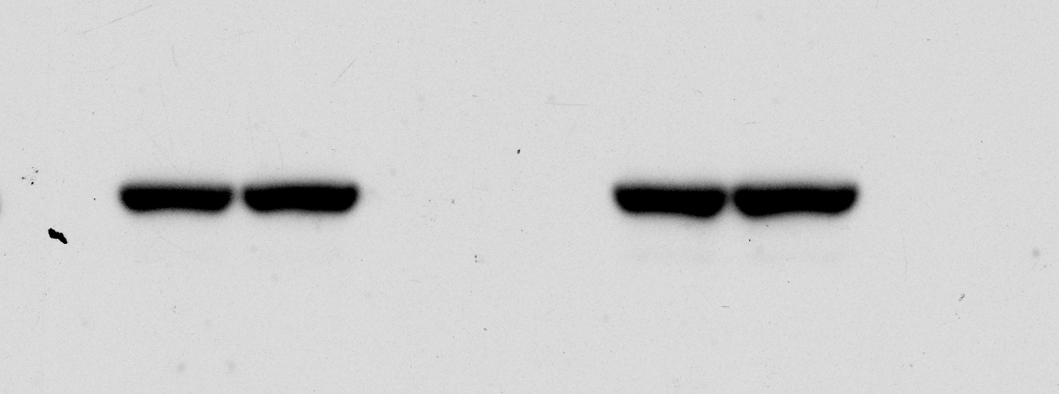

Supplement: Supplementary file 7 — Source data Fig. 5 [file 44318_2025_557_MOESM7_ESM.zip › Figure 5/5A/Figure 5A---Lysate-Lamin B1.jpg]

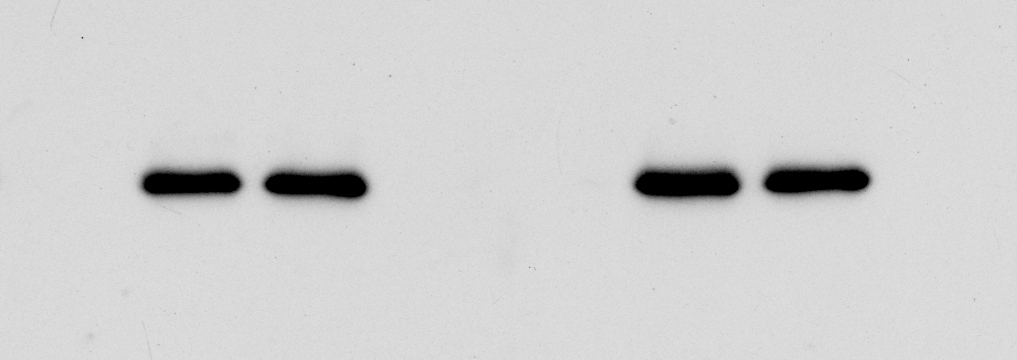

Supplement: Supplementary file 7 — Source data Fig. 5 [file 44318_2025_557_MOESM7_ESM.zip › Figure 5/5A/Figure 5A---Lysate-SMARCE1.jpg]

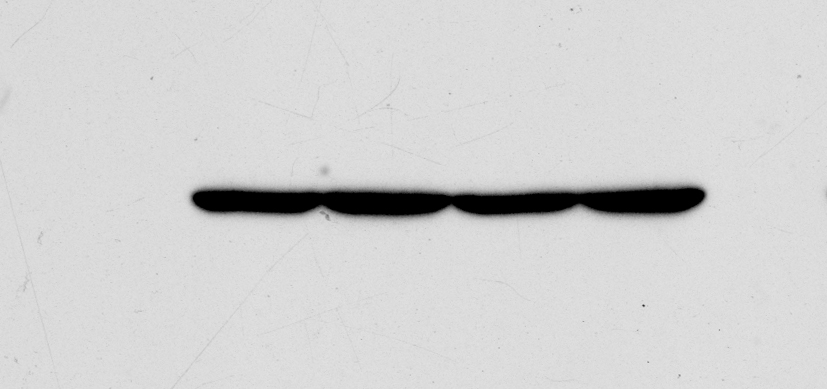

Supplement: Supplementary file 7 — Source data Fig. 5 [file 44318_2025_557_MOESM7_ESM.zip › Figure 5/5H/Figure 5H---Lysate-SMARCE1.jpg]

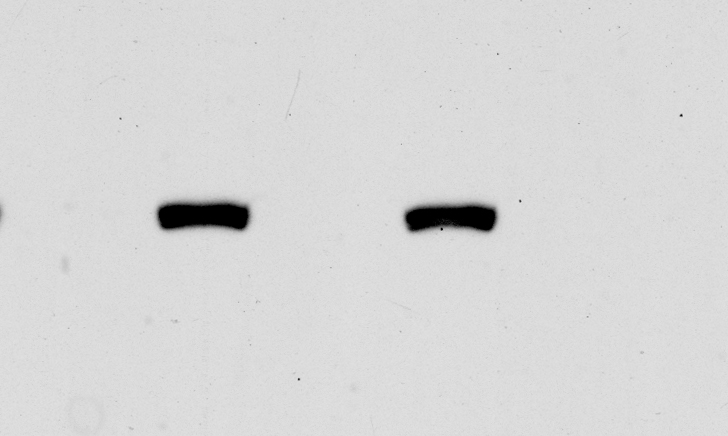

Supplement: Supplementary file 7 — Source data Fig. 5 [file 44318_2025_557_MOESM7_ESM.zip › Figure 5/5H/Figure 5H---IP-STAT1.jpg]

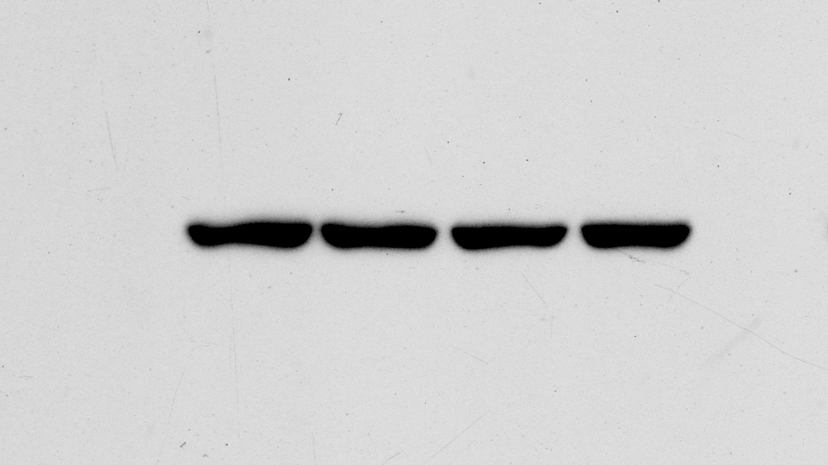

Supplement: Supplementary file 7 — Source data Fig. 5 [file 44318_2025_557_MOESM7_ESM.zip › Figure 5/5H/Figure 5H---Lysate-STAT1.jpg]

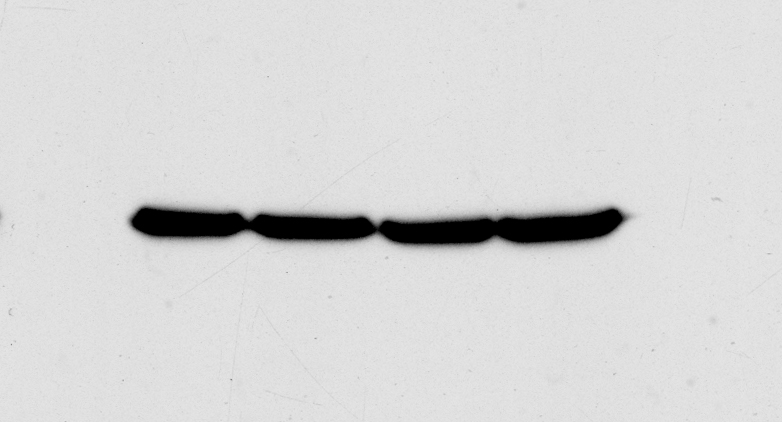

Supplement: Supplementary file 7 — Source data Fig. 5 [file 44318_2025_557_MOESM7_ESM.zip › Figure 5/5H/Figure 5H---Lysate-actin.jpg]

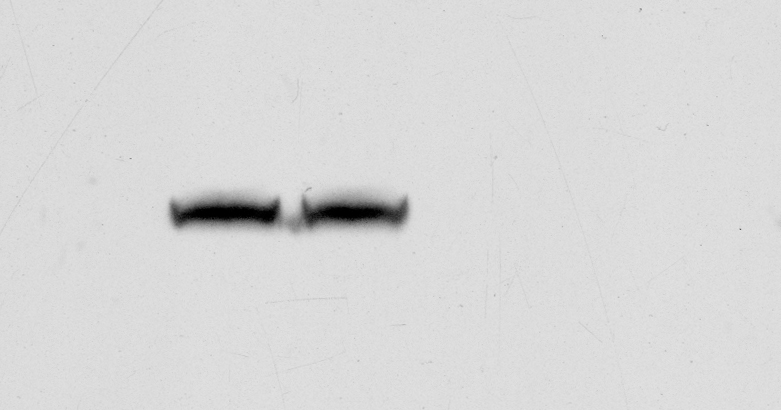

Supplement: Supplementary file 7 — Source data Fig. 5 [file 44318_2025_557_MOESM7_ESM.zip › Figure 5/5H/Figure 5H---Lysate-GLDC.jpg]

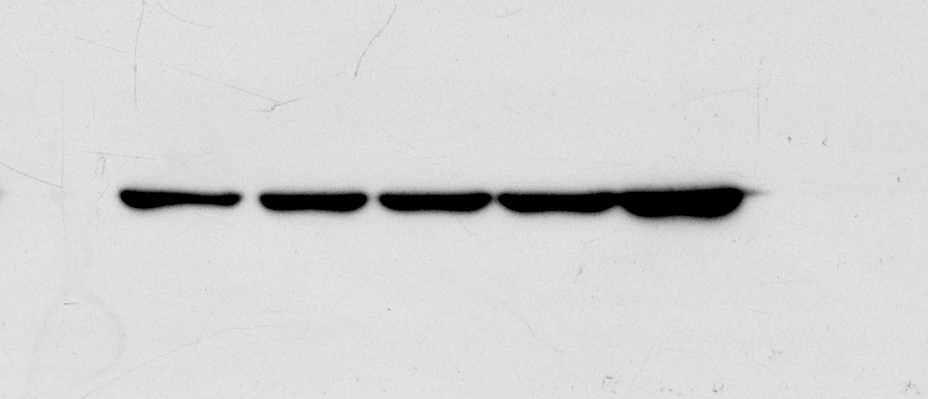

Supplement: Supplementary file 7 — Source data Fig. 5 [file 44318_2025_557_MOESM7_ESM.zip › Figure 5/5J/Figure 5J---Lysate-actin.jpg]
