# Supplementary figures and images for: Nuclear glycine decarboxylase suppresses STAT1-dependent MHC-I and promotes cancer immune evasion (part 2 of 2)
Source: EMBO J. 2025 Sep 8;44(20):5712–33. doi: 10.1038/s44318-025-00557-3 (PMC12528744; doi:10.1038/s44318-025-00557-3)

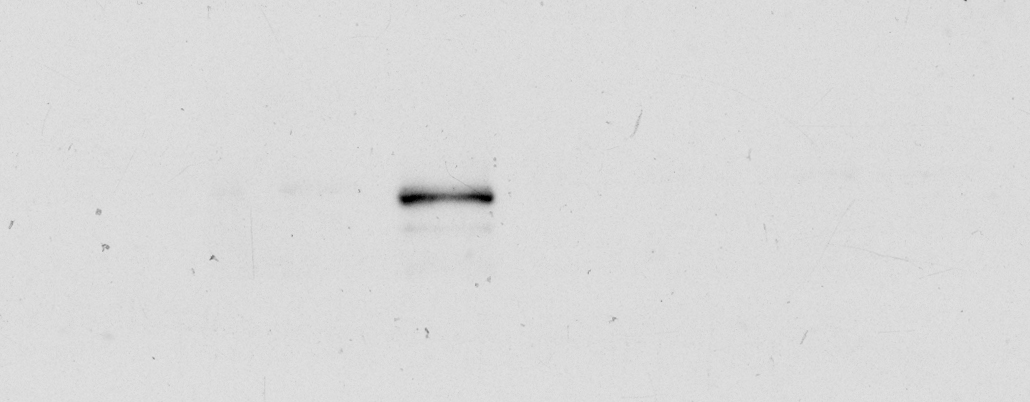

Supplement: Supplementary file 7 — Source data Fig. 5 [file 44318_2025_557_MOESM7_ESM.zip › Figure 5/5J/Figure 5J---IP-STAT1.jpg]

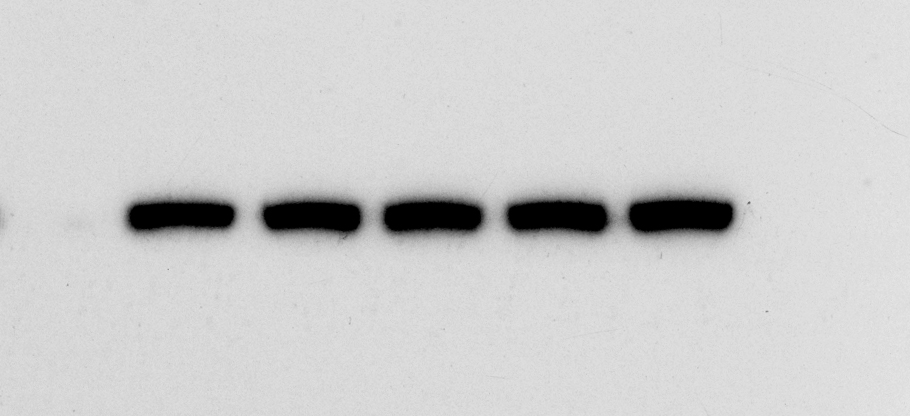

Supplement: Supplementary file 7 — Source data Fig. 5 [file 44318_2025_557_MOESM7_ESM.zip › Figure 5/5J/Figure 5J---Lysate-STAT1.jpg]

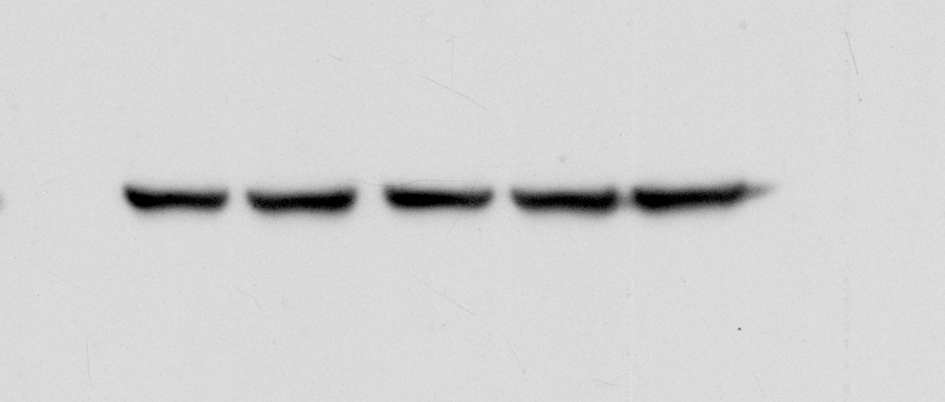

Supplement: Supplementary file 7 — Source data Fig. 5 [file 44318_2025_557_MOESM7_ESM.zip › Figure 5/5J/Figure 5J---Lysate-SMARCE1.jpg]

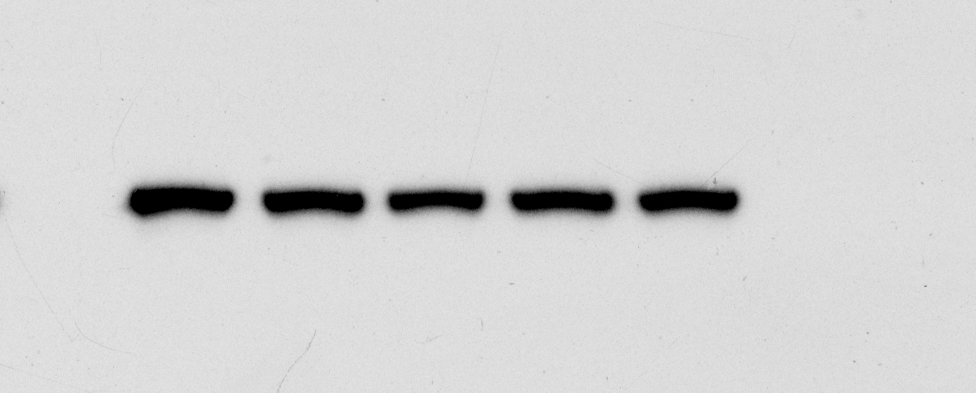

Supplement: Supplementary file 7 — Source data Fig. 5 [file 44318_2025_557_MOESM7_ESM.zip › Figure 5/5J/Figure 5J---Lysate-GLDC.jpg]

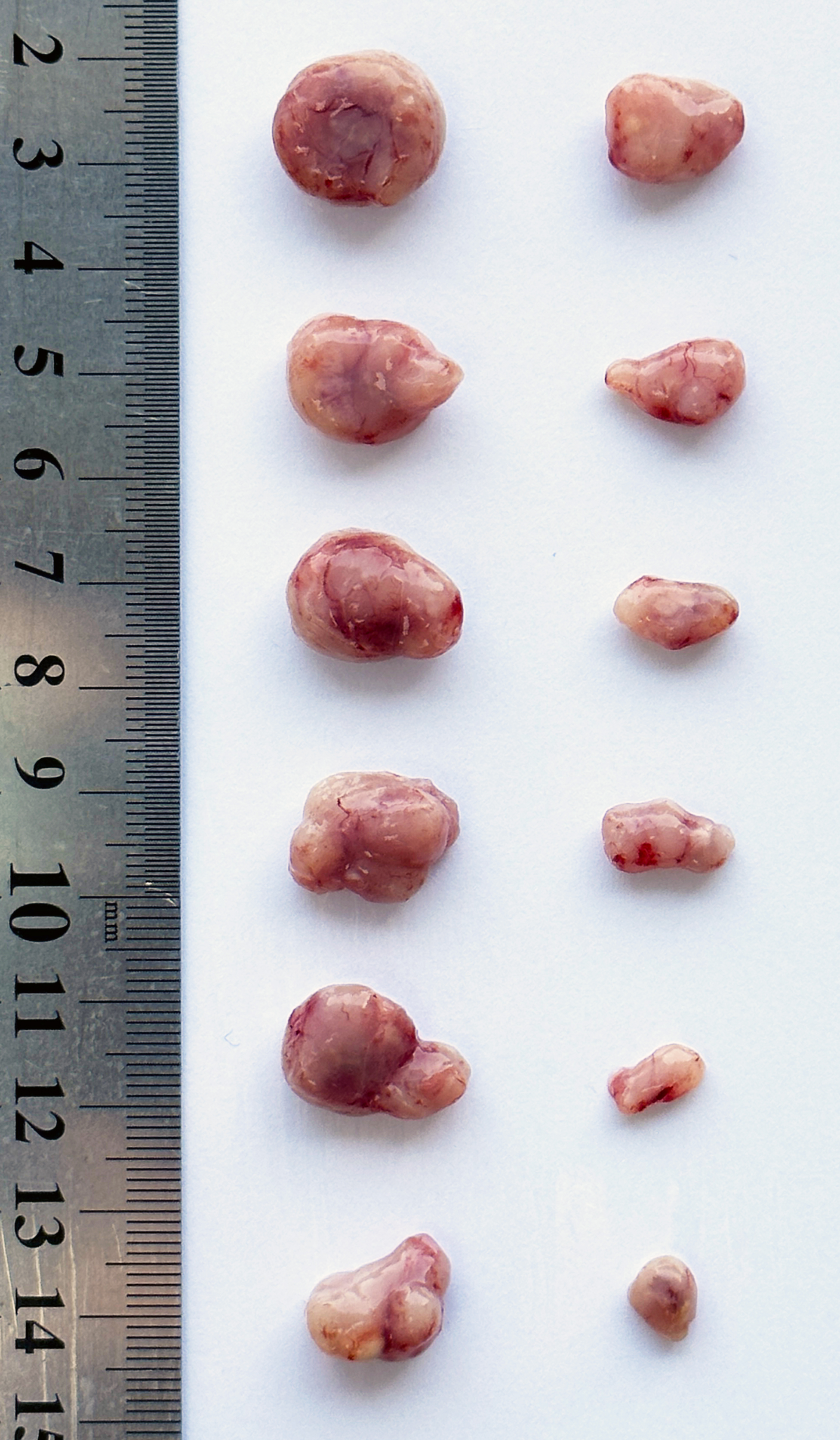

Supplement: Supplementary file 9 — Source data Fig. 7 [file 44318_2025_557_MOESM9_ESM.zip › Figure 7/7D/Figure 7D---Tumor image.jpg]

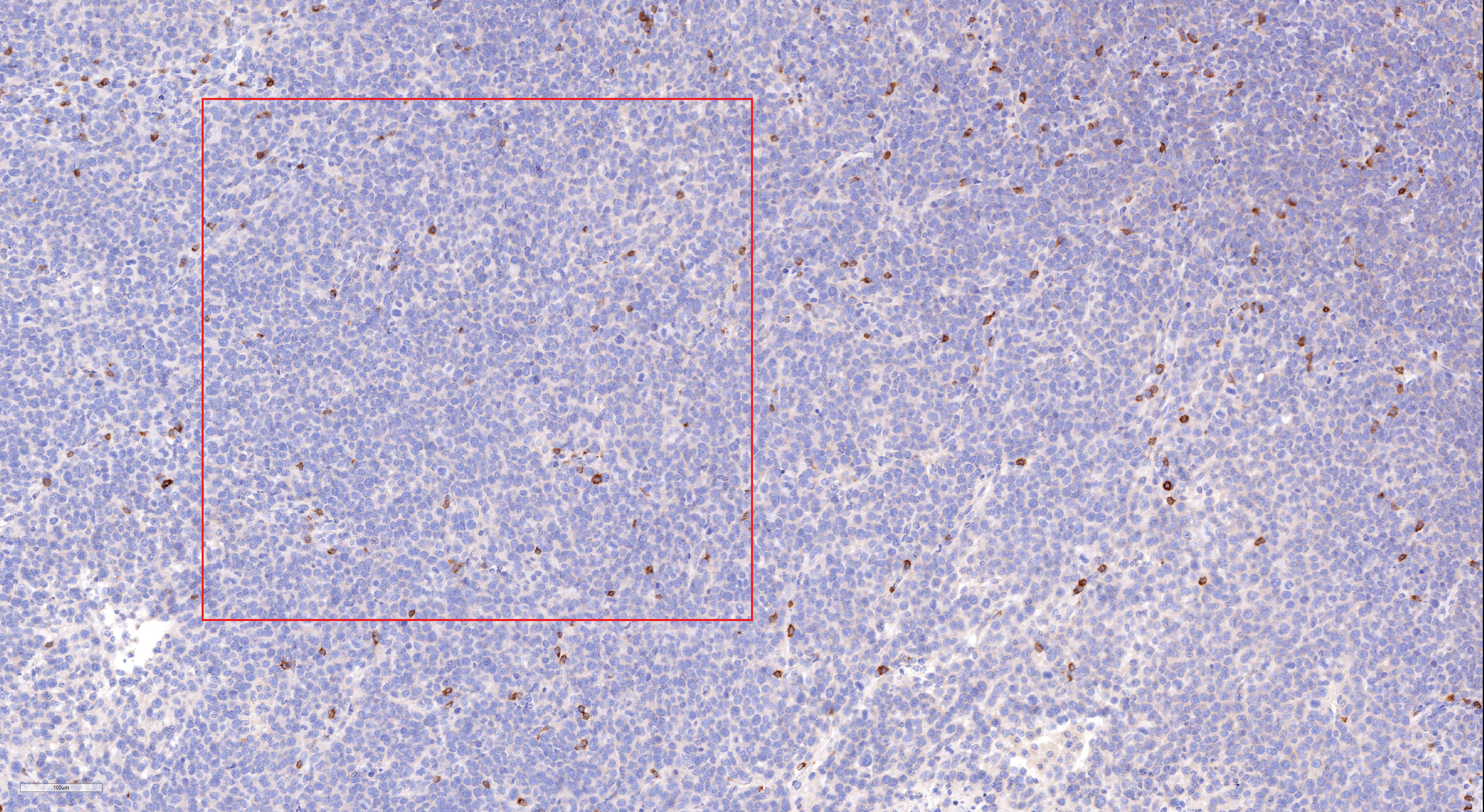

Supplement: Supplementary file 9 — Source data Fig. 7 [file 44318_2025_557_MOESM9_ESM.zip › Figure 7/7F/Figure 7F-IHC-CD8-Gldc.tif]

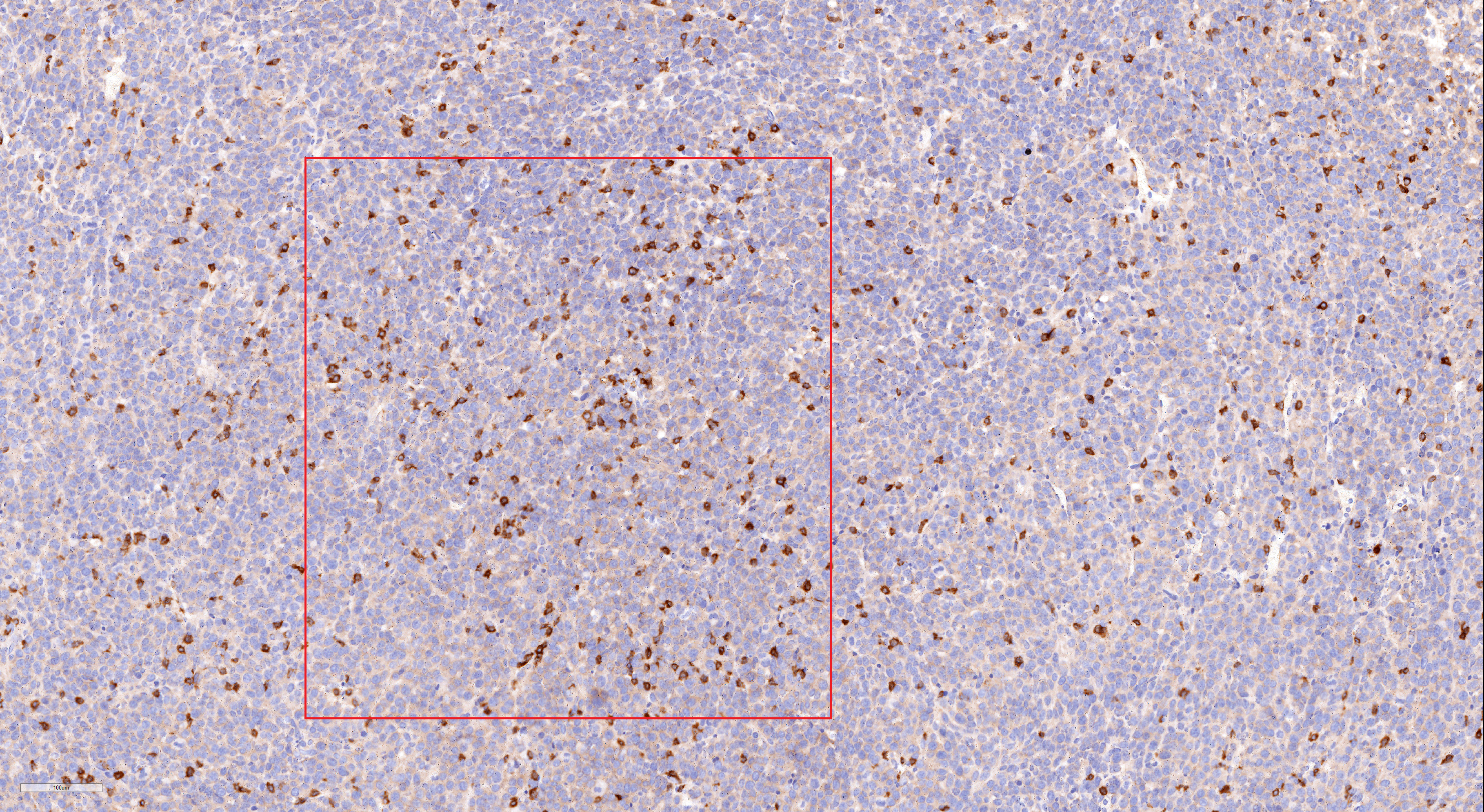

Supplement: Supplementary file 9 — Source data Fig. 7 [file 44318_2025_557_MOESM9_ESM.zip › Figure 7/7F/Figure 7F-IHC-CD8-GldcYF.tif]
