# Supplementary material for: Nuclear glycine decarboxylase suppresses STAT1-dependent MHC-I and promotes cancer immune evasion
Source: EMBO J. 2025 Sep 8;44(20):5712–33. doi: 10.1038/s44318-025-00557-3 (PMC12528744; doi:10.1038/s44318-025-00557-3)
Supplement: Supplementary file 10 — Expanded View Figures [file 44318_2025_557_MOESM10_ESM.pdf]

Expanded View Figure

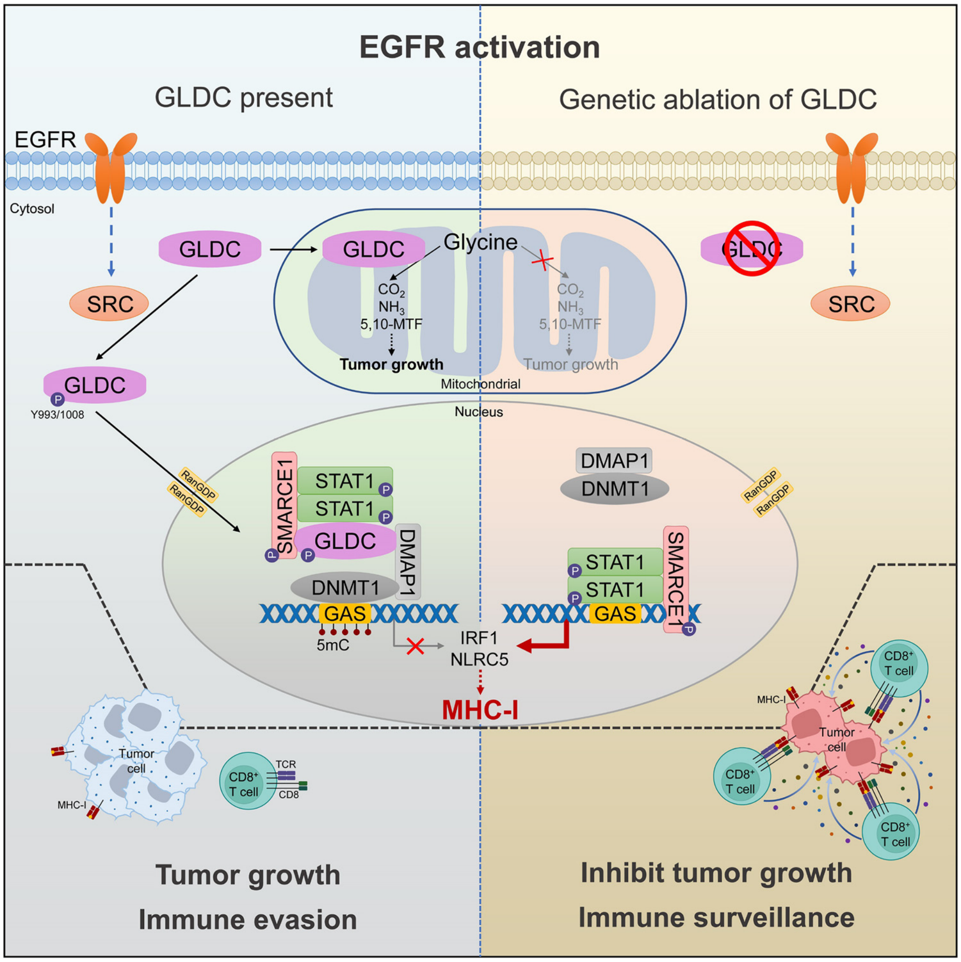

Figure EV1. A model on the regulatory of MHC-I antigen presentation by reprogramming GLDC.

 © The Author(s)
